# Preclinical identification of an oxadiazole-pyrazole lead targeting prostate cancer through integrated mechanistic evaluation

**DOI:** 10.3389/fonc.2026.1953079

**Published:** 2026-09-21

**Authors:** Vanktesh Kumar, Pankaj Wadhwa, Shubham Kumar

**Affiliations:** 1 Department of Pharmaceutical Chemistry, School of Pharmaceutical Sciences, Lovely Professional University, Phagwara, Punjab, India; 2 Department of Pharmaceutical Chemistry, Ummeed Institute of Pharmaceutical Sciences, Sri ganganagar, Rajasthan, India

**Keywords:** androgen receptor, cytotoxicity, molecular docking, oxadiazole, prostate cancer, pyrazole, reactive oxygen species

## Abstract

**Background:**

Prostate cancer remains a major cause of cancer-related mortality among men, while resistance to androgen deprivation therapy emphasizes the need for new therapeutic strategies. Molecular hybridization of pharmacologically relevant heterocycles offers a rational approach for developing novel anticancer agents.

**Methods:**

Twenty oxadiazole–pyrazole hybrids (VS-01–VS-20) were designed, synthesized, and evaluated using molecular docking against the androgen receptor (PDB ID: 1Z95), followed by *in vitro* antiproliferative assessment against androgen-dependent LNCaP and androgen-independent PC-3 prostate cancer cells. The lead compound was further investigated for its effects on androgen receptor expression, intracellular reactive oxygen species (ROS), cell-cycle progression, and apoptosis using flow cytometry and fluorescence microscopy. ADMET properties were additionally predicted computationally.

**Results:**

VS-04 demonstrated a favorable interaction profile within the androgen receptor ligand-binding pocket and exhibited potent antiproliferative activity, with IC_50_ values of 394.95 ± 3.03 nM against PC-3 and 464.85 ± 4.29 nM against LNCaP cells. Docking–activity correlation analysis revealed a moderate and significant association between docking affinity and PC-3 antiproliferative activity, whereas no significant correlation was observed for LNCaP cells. VS-04 treatment produced a concentration-dependent reduction in androgen receptor expression and increased intracellular ROS levels. Mechanistic studies further demonstrated pronounced S-phase accumulation and induction of apoptosis, with the total apoptotic population increasing from 7.45% in control cells to 47.83% following VS-04 treatment.

**Conclusion:**

The integrated computational and experimental evaluation identified VS-04 as a promising oxadiazole–pyrazole lead with potent antiproliferative activity against prostate cancer cells. Its activity was associated with AR modulation, oxidative stress, cell-cycle perturbation, and apoptotic cell death, supporting further structural optimization and preclinical investigation of this scaffold for prostate cancer therapy.

## 1 Introduction

Prostate cancer is one of the most frequently diagnosed malignancies and a leading cause of cancer-related mortality among men worldwide. Although advances in screening, diagnosis, surgery, radiotherapy, androgen deprivation therapy (ADT), chemotherapy, and targeted therapies have improved clinical outcomes, advanced and metastatic disease remains a major therapeutic challenge (1). Suppression of androgen receptor (AR) signaling remains the cornerstone of treatment; however, prolonged therapy frequently leads to resistance through alterations in AR signaling, activation of compensatory pathways, genetic mutations, and metabolic reprogramming. Consequently, many patients progress to castration-resistant prostate cancer (CRPC), an aggressive stage associated with poor prognosis, metastasis, and limited therapeutic options, emphasizing the need for novel therapeutic agents capable of overcoming therapeutic resistance (2, 3).

Among the emerging therapeutic strategies, the disruption of intracellular redox homeostasis has attracted considerable attention. Reactive oxygen species (ROS) regulate essential cellular processes, including proliferation, differentiation, and survival, whereas cancer cells maintain persistently elevated ROS levels owing to increased metabolic activity and oncogenic signaling (4). To tolerate this oxidative environment, tumor cells rely on efficient antioxidant defense systems to maintain redox balance. Excessive ROS accumulation beyond the cellular antioxidant capacity results in oxidative damage to proteins, lipids, and nucleic acids, ultimately impairing cellular integrity and promoting cancer cell death (5). Therefore, the selective induction of ROS-mediated oxidative stress has emerged as a promising therapeutic strategy for prostate cancer. Their favorable physicochemical properties and diverse biological activities, nitrogen-containing heterocyclic scaffolds, particularly 1,3,4-oxadiazole and pyrazole, are widely recognized as privileged structures in anticancer drug discovery (6). Oxadiazole derivatives have been reported to enhance metabolic stability, improve target interactions, and exhibit antiproliferative and kinase inhibitory activities. Likewise, pyrazole derivatives possess broad anticancer potential through the modulation of multiple signaling pathways involved in tumor growth and survival (7). The complementary pharmacological characteristics of these two heterocyclic systems make their molecular hybridization a rational strategy for developing multifunctional anticancer agents with enhanced therapeutic potential. Although oxadiazole-containing compounds have previously been investigated as androgen receptor modulators and pyrazole-based derivatives have been extensively explored for their anticancer properties, the integration of these two pharmacophores into a single hybrid framework for prostate cancer remains comparatively less explored. The present study was therefore designed to combine the 1,3,4-oxadiazole and pyrazole pharmacophores within a single molecular scaffold and investigate the effect of structural substitution on androgen receptor binding and antiproliferative activity. In addition to computational evaluation, the synthesized hybrids were examined against androgen-sensitive LNCaP and androgen-independent PC-3 prostate cancer cells, while the lead compound was further evaluated for modulation of androgen receptor expression and intracellular ROS generation. Thus, the present work addresses the limited exploration of oxadiazole–pyrazole hybrids as potential AR-targeting and oxidative-stress-modulating chemotypes for prostate cancer. Based on this rationale, a series of novel oxadiazole-pyrazole hybrids bearing structurally diverse electron-donating and electron-withdrawing substituents were designed and synthesized to investigate their antiproliferative potential against prostate cancer. The synthesized compounds were initially evaluated through molecular docking to predict their interactions with the androgen receptor, followed by *in vitro* cytotoxicity screening to identify the most active derivative (8, 9). The ability of the lead compound to inhibit androgen receptor expression and induce intracellular reactive oxygen species (ROS) generation was subsequently investigated, providing mechanistic insight into its antiproliferative activity. By integrating computational predictions with experimental validation, this study aimed to identify a promising oxadiazole-pyrazole lead compound for the development of new therapeutic agents against prostate cancer (**Figure 1**) (10, 11).

**Figure 1:**
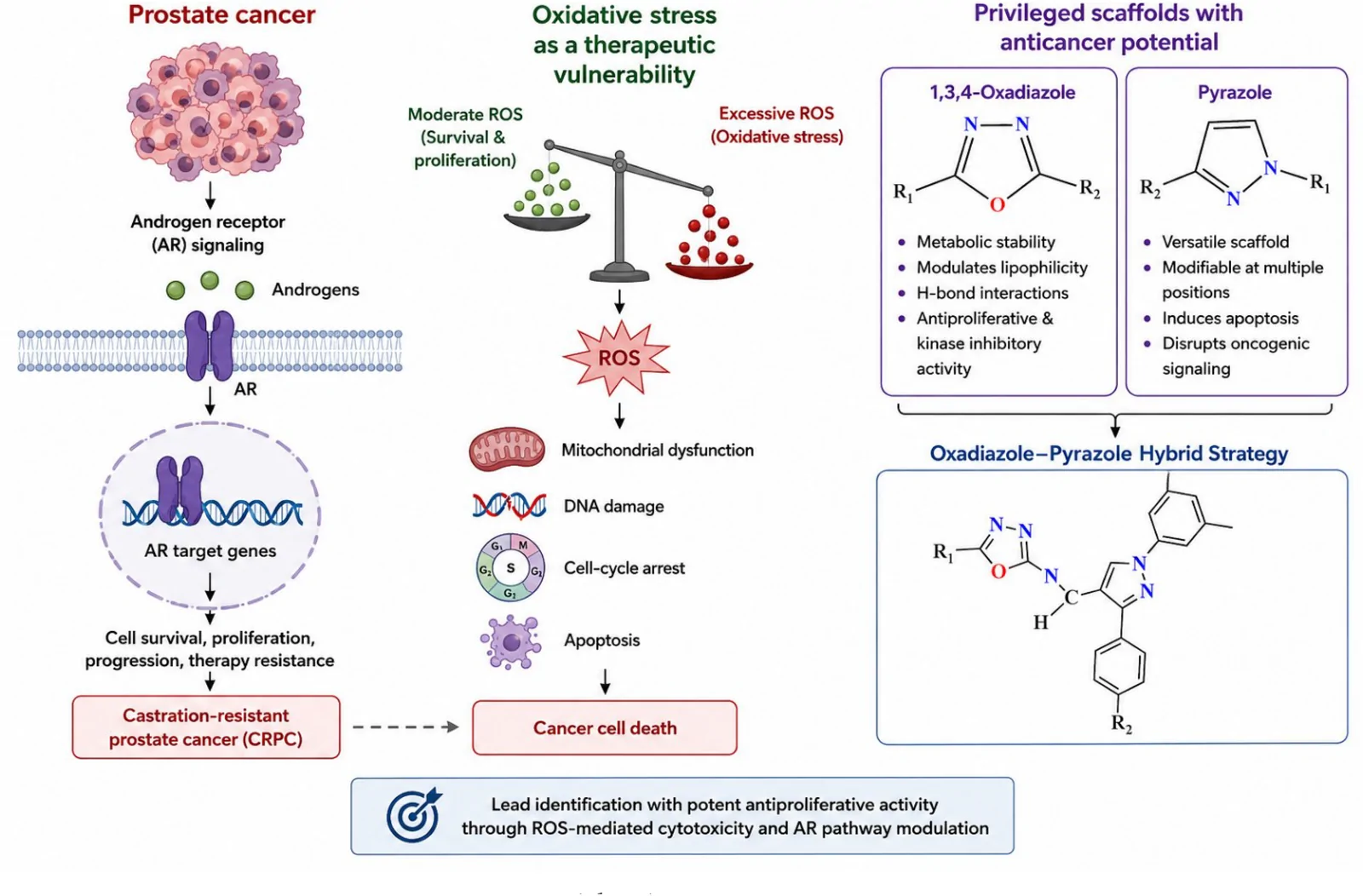
Rationale of oxadiazole hybrids as potential anti-prostate cancer agents.

### 1.1 Study rationale and objective

Prostate cancer is highly dependent on androgen receptor (AR) signaling, and the emergence of therapeutic resistance necessitates the development of novel agents capable of targeting complementary cellular vulnerabilities (12). On the basis of this rationale, the present study aimed to design and synthesize a series of novel oxadiazole-pyrazole hybrids and identify a lead compound with potent antiproliferative activity against prostate cancer cells. Following molecular docking, in silico drug-likeness evaluation, chemical synthesis, and *in vitro* cytotoxicity screening, the most active compound was selected for mechanistic evaluation through androgen receptor inhibition and generation of intracellular reactive oxygen species (ROS). By integrating computational predictions with biological validation, this study sought to establish the therapeutic potential of a novel oxadiazole-pyrazole derivative as a promising lead candidate for prostate cancer treatment. The present study represents a systematic extension of our previous investigation of oxadiazole-pyrazole-Schiff base derivatives, in which a subset of compounds from the broader synthesized series was previously reported for their antiproliferative and metabolic modulatory activities (13). In the present work, the oxadiazole-pyrazole scaffold was further investigated to establish a more comprehensive structure–activity relationship and to extend the biological evaluation toward androgen receptor (AR)-associated mechanisms relevant to prostate cancer. The broader synthetic program comprised 30 derivatives, of which 10 compounds were reported in our earlier study, while the present manuscript focuses on the remaining 20 derivatives (VS-01–VS-20). The compounds were designed using a focused molecular-hybridization strategy, with systematic variation of the R¹ and R² substituents to modulate the electronic, steric, and lipophilic properties of the scaffold. This approach enabled comparative assessment of the substitution pattern across the expanded series and facilitated identification of the most promising derivative for subsequent mechanistic investigation. Accordingly, the present study should be viewed as a complementary and mechanistically extended investigation of the oxadiazole–pyrazole chemotype rather than as an independent redesign of an unrelated compound series.

## 2 Materials and methods

### 2.1 Chemicals and reagents

All chemicals and reagents used in this study were of analytical grade and purchased from commercial suppliers without further purification unless otherwise stated. The solvents used for synthesis and purification were of synthetic grade. The reaction progress was monitored by thin-layer chromatography (TLC) using 60 F254 aluminum plates with silica gel. Column chromatography was performed on silica gel (100–200 mesh) for purification of the synthesized compounds. Melting points were determined using a digital melting point apparatus and were reported without correction. The synthesized oxadiazole–pyrazole derivatives (VS-01–VS-20) were characterized using Fourier transform infrared (FT-IR) spectroscopy, ^1H nuclear magnetic resonance (^1H NMR), ^13C nuclear magnetic resonance (^13C NMR), and high-resolution mass spectrometry (HRMS). The detailed synthetic procedures and complete spectral characterization data are provided in the **Supplementary Information** (14–16).

### 2.2 Molecular docking

The interactions of the synthesized compounds with the androgen receptor (AR) ligand-binding domain were investigated using molecular docking. The crystal structure of the human AR was obtained from the Protein Data Bank (PDB ID: 1Z95) (17, 18). Protein preparation involved the removal of water molecules and cocrystallized ligands, the addition of polar hydrogen atoms, and the assignment of Kollman charges using AutoDock Tools (19). The three-dimensional structures of the synthesized compounds were generated in ChemDraw, and the energy was minimized prior to docking. Molecular docking was performed using AutoDock Vina, and the binding affinities were expressed as kcal/mol. The docking protocol was validated through redocking of the native ligand. Binding interactions, including hydrogen bonding, hydrophobic contacts, π-π interactions, and π-alkyl interactions, were visualized using the BIOVIA Discovery Studio Visualizer (20, 21). The docking region was defined based on the experimentally characterized ligand-binding pocket of the AR crystal structure, corresponding to the spatial region occupied by the cocrystallized ligand in PDB ID 1Z95 (22). To further assess the appropriateness of the selected docking region, the same protein structure was subsequently analyzed using CASTp (23). CASTp identified a principal binding pocket (Pocket 1) with a surface area of 263.16 Å² and a volume of 141.15 Å³ (**Supplementary Figure S1**). The residue and atom composition of this pocket is provided in **Supplementary Table S1**. Notably, residues including Leu704, Leu707, Gly708, Leu741, Met742, and Met745 were identified within the CASTp-predicted pocket and were also represented in the binding environment obtained from our docking analysis. The agreement between the CASTp-defined pocket and the docking-derived binding environment provides additional structural support for the selection of the predefined AR ligand-binding region for molecular docking. The docking protocol was validated by redocking the co-crystallized ligand, which yielded an RMSD of 0.71 Å relative to its experimentally determined crystallographic pose. As this value is substantially below the generally accepted threshold of 2.0 Å, the docking protocol was considered reliable for reproducing the experimentally observed binding mode (24). The corresponding superimposition and RMSD analysis are presented in **Supplementary Figure S2**.

### 2.3 Cell culture

The human androgen-sensitive prostate cancer cell line LNCaP was obtained from a certified cell repository and cultured in RPMI-1640 medium supplemented with 10% fetal bovine serum (FBS), 100 U/mL penicillin, and 100 μg/mL streptomycin. The cells were maintained at 37 °C in a humidified incubator containing 5% CO_2_ and subcultured upon reaching approximately 80% confluence. All experiments were performed using cells in the logarithmic growth phase (25).

### 2.4 *In vitro* cytotoxicity assay

The antiproliferative activity of compounds VS01-VS20 was evaluated using the MTT colorimetric assay. LNCaP cells were seeded into 96-well plates at an optimized density and allowed to attach overnight. The cells were subsequently exposed to increasing concentrations of the synthesized compounds for 24 h, and bicalutamide served as the reference drug. Following treatment, MTT solution (5 mg/mL) was added to each well and incubated for 3 h at 37 °C. The resulting purple formazan crystals were dissolved in dimethyl sulfoxide (DMSO), and the absorbance was measured at 570 nm using a microplate reader. Cell viability was calculated relative to that of untreated control cells, and the IC_50_ values were determined using nonlinear regression analysis using GraphPad Prism (26, 27).

### 2.5 Androgen receptor inhibition assay

The effect of the lead compound on androgen receptor expression was determined using a flow cytometry-based immunostaining assay. LNCaP cells were seeded in 12-well plates and treated with different concentrations of the selected compound for 24 h. Bicalutamide was used as the positive control. Following treatment, the cells were harvested, washed with phosphate-buffered saline (PBS), and fixed with 4% paraformaldehyde. The cells were permeabilized with chilled methanol and incubated with a rabbit anti-AR primary antibody. After being washed, the cells were incubated with an Alexa Fluor 488-conjugated secondary antibody in the dark. The samples were analyzed using flow cytometry, and androgen receptor expression was quantified as the mean fluorescence intensity using FlowJo software.

### 2.6 Intracellular reactive oxygen species assay

Intracellular ROS generation was quantified using the fluorescent probe 2’,7’-dichlorodihydrofluorescein diacetate (DCFH-DA). LNCaP cells were seeded into 12-well plates and treated with the selected compound for 24 h. Following treatment, the cells were incubated with DCFH-DA working solution at 37 °C for 30 min in the dark. Excess probe was removed by washing with PBS, and the fluorescence intensity was measured using flow cytometry. The intracellular ROS level is expressed as the mean fluorescence intensity relative to that of untreated control cells.

### 2.7 Apoptosis

Apoptosis induction by VS-04 was evaluated using Annexin V–FITC/propidium iodide (PI) dual staining followed by flow-cytometric analysis. The assay was performed to determine whether the antiproliferative effect of VS-04 was associated with induction of programmed cell death. Annexin V staining was used to identify cells undergoing phosphatidylserine externalization, whereas PI staining was used to distinguish cells with compromised membrane integrity. Following treatment with VS-04, cells were stained with Annexin V and PI and analyzed by flow cytometry. A total of 10,000 cellular events were acquired for each sample, and the resulting populations were categorized into viable cells (Annexin V−/PI−), early apoptotic cells (Annexin V+/PI−), late apoptotic cells (Annexin V+/PI+), and necrotic cells (Annexin V−/PI+). Two flow-cytometric datasets were evaluated for the VS-04-treated group (B07 and B08) and two corresponding datasets were analyzed for the control group (D11 and D12), allowing comparison of the distribution of viable and apoptotic cell populations following VS-04 exposure (28).

### 2.8 Cell cycle analysis

Cell-cycle distribution following treatment with VS-04 was evaluated by flow-cytometric analysis based on cellular DNA content. The analysis was performed by measuring the fluorescence intensity of PI-A, which reflects the DNA content of individual cells and permits discrimination of cells according to their position within the cell cycle. A total of 10,000 events were acquired for both the untreated control (D11) and VS-04-treated sample (B07). The resulting DNA-content histograms were analyzed to determine the proportion of cells distributed in the G0/G1, S, and M phases of the cell cycle. The percentage of events within each phase was obtained from the respective gated regions of the flow-cytometric histograms. The untreated control and VS-04-treated populations were subsequently compared to determining treatment-associated alterations in cell-cycle progression and identifying the phase in which VS-04 preferentially accumulated the cells (29, 30).

## 3 Results

### 3.1 Synthesis and characterization

A series of 20 oxadiazole-pyrazole derivatives (VS01–VS20) were successfully synthesized as extension to our previous synthesized series and purified for subsequent biological evaluation. Structural confirmation of all the compounds was achieved using Fourier transform infrared (FT-IR) spectroscopy, ^1H nuclear magnetic resonance (^1H NMR), ^13C nuclear magnetic resonance (^13C NMR), and high-resolution mass spectrometry (HRMS), which verified the successful formation of the designed molecular framework. The synthesized derivatives were obtained in satisfactory yields and exhibited adequate purity for computational and *in vitro* studies. A summary of the physicochemical characteristics of the synthesized compounds, including their substituent patterns, reaction yields, and melting points, is presented in **Supplementary File**. The detailed synthetic procedure (**Supplementary Scheme S1**), together with complete spectroscopic characterization and analytical data for all the compounds, is provided in the **Supplementary Information**. Following structural confirmation, the synthesized compound library was subjected to molecular docking, in silico pharmacokinetic assessment, and biological screening to identify potential anti-prostate candidates.

### 3.2 Molecular docking analysis reveals potential AR inhibitors

Molecular docking was performed to evaluate the binding affinity of the synthesized oxadiazole–pyrazole derivatives (VS01–VS20) toward the androgen receptor (PDB ID: 1Z95) using AutoDock Vina. Docking calculations were carried out with a grid box centered at x = 27.744, y = 3.191, and z = 7.790 Å and dimensions of 40 × 40 × 40 Å³. The best-ranked binding pose was selected for interaction analysis.

The synthesized compounds presented docking scores ranging from −8.4 to −11.1 kcal/mol. VS04 showed the highest binding affinity (−11.1 kcal/mol), outperforming the reference drug bicalutamide (−10.8 kcal/mol). As VS04 also demonstrated superior biological activity, it was selected as the lead compound for subsequent mechanistic studies. Interaction analysis revealed that VS04 formed a conventional hydrogen bond with Lys808, together with carbon hydrogen bonding, π-cations, amide–π stacking, alkyl, π-alkyl, and van der Waals interactions involving Arg752, Glu681, Ala748, Pro682, Val715, Trp718, Leu744, and Met745, indicating stable accommodation within the AR binding pocket. In comparison, bicalutamide established multiple hydrogen bonds with Gln711, Arg752, Leu704, and Hiss874, along with hydrophobic interactions involving Leu701, Leu707, Leu741, Met742, Met745, Phe764, and Val746 (**Supplementary Figure S3**). Collectively, these findings indicate that VS04 has a favorable binding profile toward the AR and support its selection for further biological evaluation (**Supplementary Table S2**).

### 3.3 Cytotoxicity screening identifies the lead compound against LNCaP cells

The antiproliferative potential of the synthesized oxadiazole-pyrazole derivatives (VS-01–VS-20) was investigated against androgen-sensitive LNCaP and androgen-independent PC-3 prostate cancer cell lines using the MTT assay. All the compounds inhibited cell viability in a concentration-dependent manner, although their cytotoxic potencies varied considerably depending on the structural substitution pattern (**Table 1**). In the LNCaP cell line, the calculated IC_50_ values ranged from 464.85 to 1736.15 ± 1.41 nM. Among the synthesized derivatives, VS-04 demonstrated the strongest antiproliferative activity, with the lowest IC_50_ value (464.85 ± 4.29 nM), followed by VS-12 (529.32 ± 4.01 nM), VS-14 (558.08 ± 3.86 nM), VS-20 (575.51 ± 3.74 nM), and VS-10 (580.00 ± 3.70 nM). In contrast, compounds VS-03 (1736.15 ± 1.41 nM), VS-17 (1664.70 ± 1.49 nM), VS-02 (1359.63 ± 1.79 nM), and VS-13 (1121.44 ± 2.15 nM) exhibited comparatively weaker cytotoxic activity, indicating that structural modifications substantially influence their antiproliferative efficacy. A comparable trend was observed in the PC-3 cell line, where the IC_50_ values ranged from 394.95 ± 3.03 to 1077.41 ± 1.42 nM. VS-04 again emerged as the most active derivative, with the lowest IC_50_ value (394.95 ± 3.03 nM), followed by VS-13 (460.92 ± 2.82 nM), VS-02 (473.46 ± 2.77nM), and VS-18 (533.79 ± 2.47 nM). The least active compounds against PC-3 cells were VS-17 (1077.41 ± 1.42 nM), VS-16 (990.54 ± 1.53 nM), and VS-09 (910.18 ± 1.71 nM). Although the reference drug bicalutamide exhibited greater potency than most synthesized compounds did, the newly developed derivatives demonstrated measurable antiproliferative activity in both prostate cancer cell models (**Figure 2**; **Supplementary Figure S4**).

**Table 1:** IC_50_ of all synthesized compounds in two different cell lines.

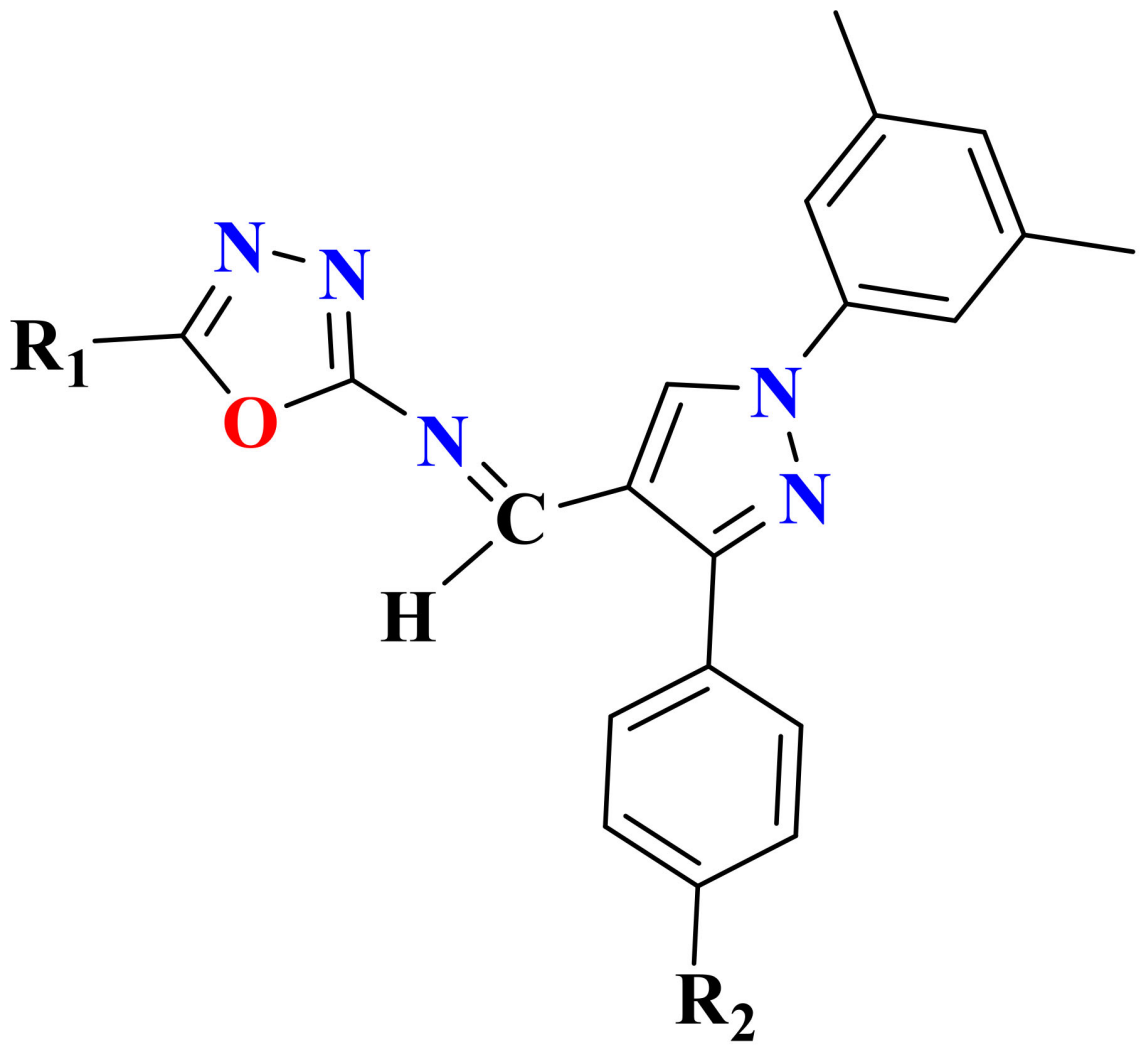					
S. no.	Compound	R_1_	R_2_	PC-3 IC_50_ ± SD (nM)	LNCaP IC_50_ ± SD (nM)
1	VS-01	H	H	909.75 ± 1.69	952.41 ± 2.41
2	VS-02	4-F	H	473.46 ± 2.77	1359.63 ± 1.79
3	VS-03	4-OCH_3_	H	616.42 ± 2.30	1736.15 ± 1.41
**4**	**VS-04**	**CF** **_3_**	**H**	**394.95** ± 3.03	**464.85** ± 4.29
5	VS-05	4-*tert*-butyl	H	635.87 ± 2.26	901.29 ± 2.58
6	VS-06	H	2,5-*dihydroxy*	561.2 ± 2.44	677.68 ± 3.33
7	VS-07	4-F	2,5-*dihydroxy*	810.63 ± 1.89	976.67 ± 2.40
8	VS-08	4-OCH_3_	2,5-*dihydroxy*	587.56 ± 2.39	698.17 ± 3.26
9	VS-09	CF_3_	2,5-*dihydroxy*	910.18 ± 1.71	752.35 ± 3.01
10	VS-10	4-*tert*-butyl	2,5-*dihydroxy*	580.02 ± 2.40	580.00 ± 3.70
11	VS-11	H	3-nitro	747.5 ± 2.02	795.88 ± 2.87
12	VS-12	4-F	3-nitro	854.62 ± 1.81	529.32 ± 4.01
13	VS-13	4-OCH_3_	3-nitro	460.92 ± 2.82	1121.44 ± 2.15
14	VS-14	CF_3_	3-nitro	769.63 ± 1.88	558.08 ± 3.86
15	VS-15	4-*tert*-butyl	3-nitro	749.65 ± 1.91	829.97 ± 2.78
16	VS-16	H	4-*hydroxy*	990.54 ± 1.53	691.29 ± 3.31
17	VS-17	4-F	4-*hydroxy*	1077.41 ± 1.42	1664.7 ± 1.49
18	VS-18	4-OCH_3_	4-*hydroxy*	533.79 ± 2.47	962.34 ± 2.47
19	VS-19	CF_3_	4-*hydroxy*	622.59 ± 2.24	784.25 ± 2.96
20	VS-20	4-*tert*-butyl	4-*hydroxy*	769.35 ± 1.90	575.51 ± 3.74
**Reference Drug**	**Bicalutamide**	**-**	**-**	**412.61** ± 3.05	**118.31** ± 4.54

The bold value corresponding to VS-04 indicates the compound with the lowest IC_50_ value among the tested compounds. Therefore, VS-04 was selected and carried forward for subsequent mechanistic studies.

**Figure 2:**
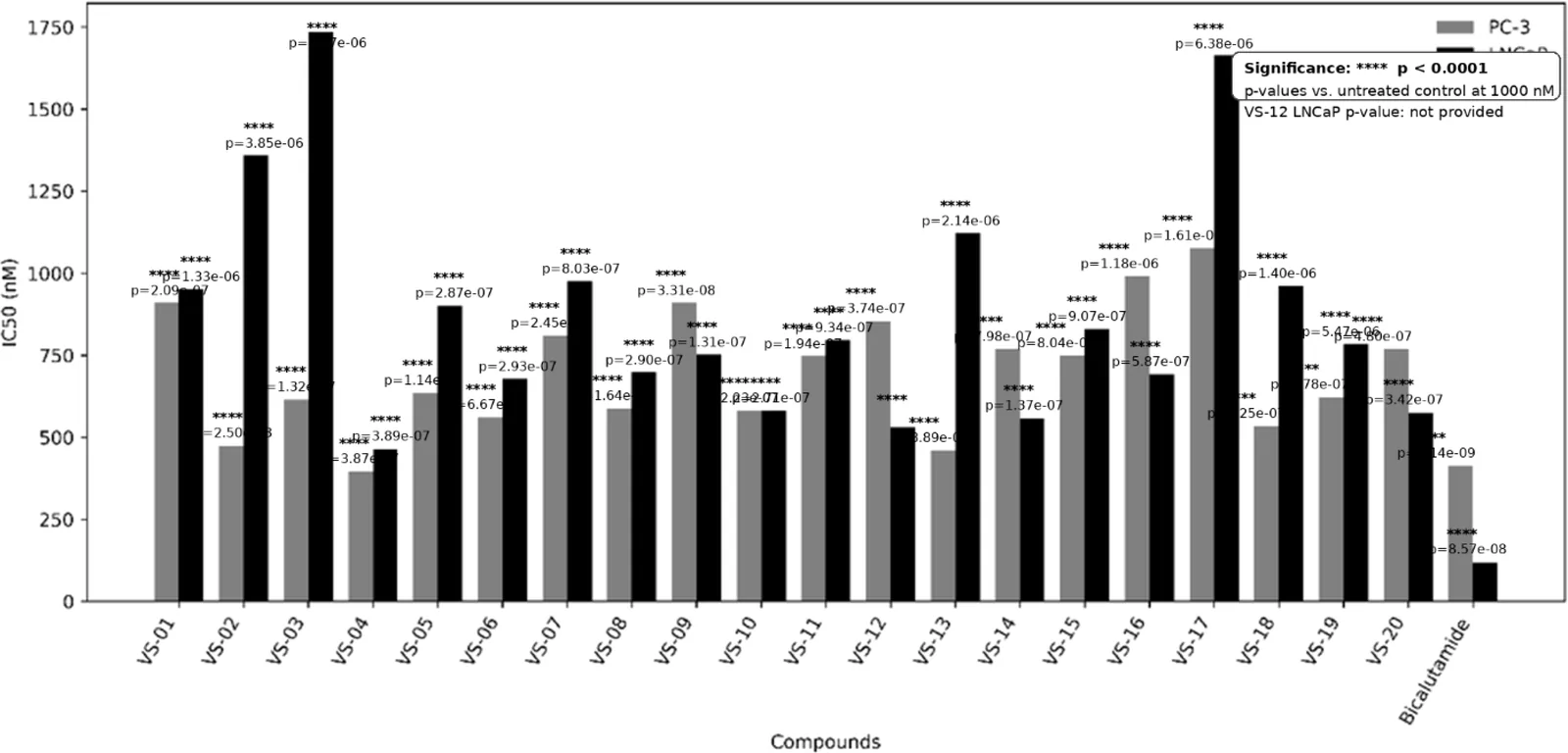
Comparative antiproliferative activity of the synthesized oxadiazole–pyrazole derivatives (VS-01–VS-20) against PC-3 and LNCaP prostate cancer cells, as determined by the MTT assay. Gray and black bars represent PC-3 and LNCaP cells, respectively. IC_50_ values are expressed as nM. VS-04 exhibited the greatest antiproliferative potency across both cell lines and was selected as the lead compound for subsequent mechanistic investigations.

### 3.4 Structure-activity relationship studies

Among the synthesized oxadiazole–pyrazole derivatives, VS-04 exhibited the highest antiproliferative activity against both PC-3 and LNCaP cells, with IC_50_ values of 394.95 ± 3.03 and 464.85 ± 4.29 nM, respectively. The SAR analysis indicated that activity was strongly influenced by the combined nature of the R¹ and R² substituents rather than by the electronic character of either substituent alone. Within the R² = H series (VS-01–VS-05), replacement of H at R¹ with 4-F markedly improved PC-3 activity, whereas incorporation of 4-OCH_3_ produced comparatively weak LNCaP activity. In contrast, the 4-CF_3_ analog VS-04 showed the most pronounced improvement in both cell lines, suggesting that this substitution provides a favorable balance of electronic and lipophilic properties. The 4-tert-butyl analog VS-05 retained moderate activity but was less potent than VS-04. Modification of R² substantially altered the activity profile. In the 2,5-dihydroxy series (VS-06–VS-10), 4-OCH_3_ (VS-08) and 4-tert-butyl (VS-10) provided relatively favorable activity, whereas the 4-CF_3_ analog VS-09 showed reduced potency, indicating that the effect of R¹ was dependent on the substitution at R². Similarly, the 3-nitrophenyl series (VS-11–VS-15) displayed differentiated activity between the two cell lines: VS-13 (4-OCH_3_) was most active against PC-3 cells (460.92 ± 2.82 nM), whereas VS-12 (4-F) and VS-14 (4-CF_3_) showed greater potency against LNCaP cells (529.32 and 558.08 ± 3.86 nM, respectively). In the 4-hydroxy series (VS-16–VS-20), VS-18 (4-OCH_3_) showed the highest PC-3 activity, while VS-20 (4-tert-butyl) was the most potent against LNCaP cells. Conversely, VS-17 bearing 4-F exhibited the weakest activity in this subset. these findings demonstrate a context-dependent SAR, in which modification at R¹ produces different effects depending on the physicochemical and hydrogen-bonding environment imposed by R². The superior activity of VS-04 was consistent with its favorable AR docking score (−11.1 kcal/mol) and its predicted interaction with Lys808 and several hydrophobic residues within the AR ligand-binding pocket. Thus, the 4-CF_3_/R²-H substitution pattern appears particularly favorable within the present series, although the divergent activity profiles across PC-3 and LNCaP cells indicate that cellular potency is influenced by factors beyond receptor binding alone. These observations provide a rational basis for further structural optimization of the oxadiazole–pyrazole scaffold (**Figure 3**).

**Figure 3:**
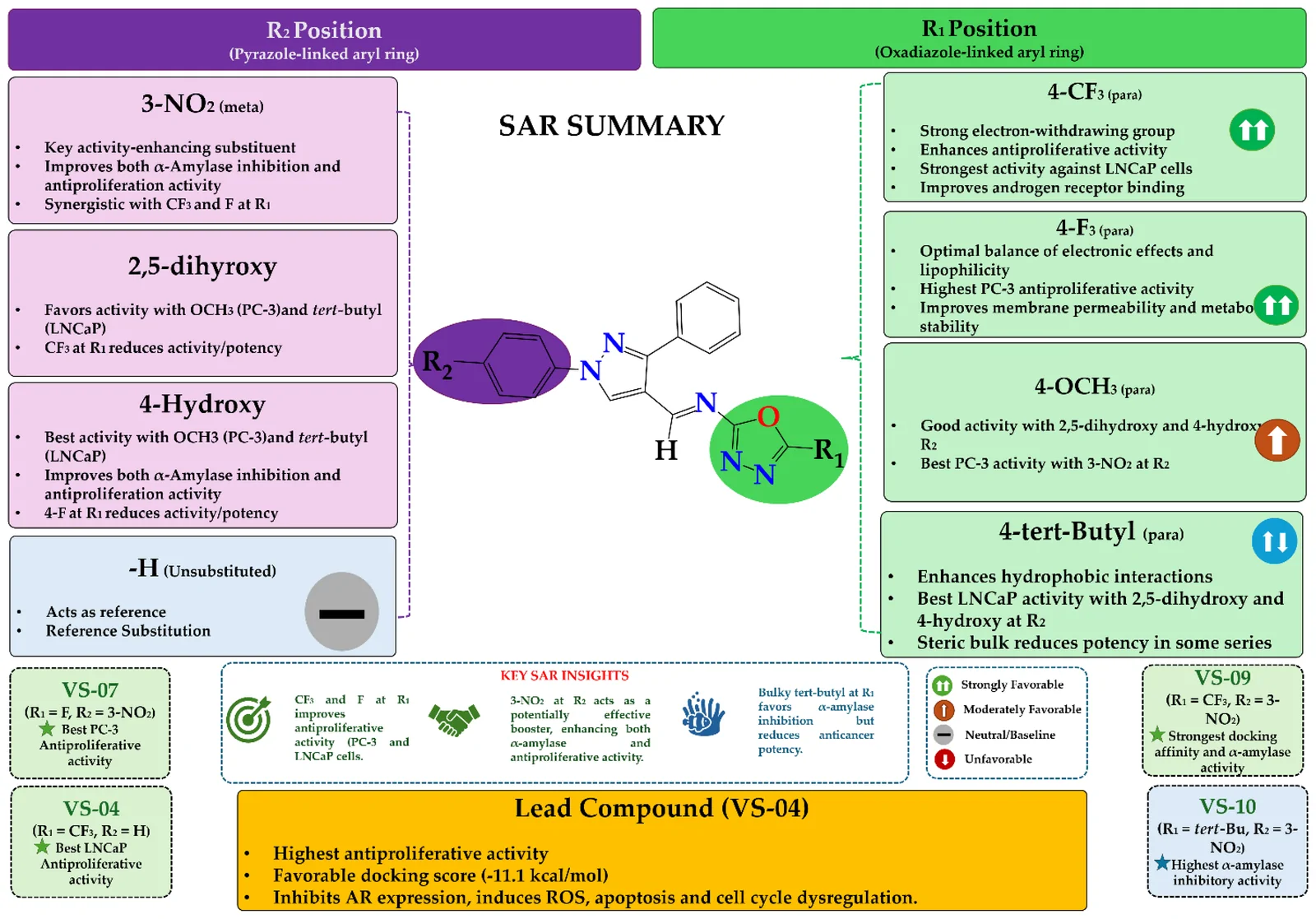
Structure–activity relationship (SAR) analysis of the synthesized oxadiazole-pyrazole derivatives (VS-01–VS-20).

### 3.5 Lead compounds inhibit androgen receptor expression in LNCaP cells

To analyze whether the antiproliferative activity of the lead compound was associated with modulation of androgen receptor (AR) expression, flow-cytometric analysis was performed in LNCaP cells following treatment with VS-04. AR-associated fluorescence was quantified using the Comp-FL1-A to mean fluorescence intensity (MFI) as the quantitative readout. As summarized in **Table 2**, the samples analyzed showed distinct fluorescence intensities, with Mean Comp-FL1-A values ranging from 3,137 to 24,021 across the evaluated populations. The corresponding analyzed cell counts ranged from 2,806 to 5,108 cells. These differences in fluorescence intensity indicate variation in the AR-associated signal among the evaluated samples. The quantitative flow-cytometry data therefore provide evidence of differential AR expression following treatment; however, interpretation of the concentration-dependent response requires assignment to their respective treatment conditions.

**Table 2:** Flow-cytometric quantification of AR-associated fluorescence in LNCaP cells.

Sample	Concentration (nM)	Cell count (n)	Mean AR-associated fluorescence intensity (Comp-FL1-A)
Control	–	2,795	3,251
VS-04	100	4,544	12,810
VS-04	200	5,125	20,741
Bicalutamide	200	5,057	24,121

### 3.6 Lead compounds induce intracellular reactive oxygen species generation

To analyze whether the antiproliferative activity of VS-04 was associated with oxidative stress, intracellular reactive oxygen species (ROS) generation was assessed in LNCaP cells using the DCFDA assay followed by flow cytometric analysis. The cells were treated with VS-04 (100 and 200 nM), while bicalutamide (200 nM) served as the reference drug. Representative flow cytometry dot plots are shown in **Figure 4A**. Untreated control cells exhibited minimal DCFDA fluorescence, indicating low basal ROS production. Compared with the control, treatment with VS-04 at 100 nM resulted in a marked increase in intracellular ROS levels. A further increase in DCFDA fluorescence was observed following treatment with VS-04 at 200 nM, demonstrating a concentration-dependent induction of oxidative stress. Bicalutamide (200 nM) also increased ROS production, resulting in the highest fluorescence intensity among all the treatment groups. These findings indicate that VS-04 promotes intracellular ROS accumulation, suggesting that oxidative stress contributes to its antiproliferative activity and may participate in the subsequent induction of cell cycle arrest and apoptosis.

**Figure 4:**
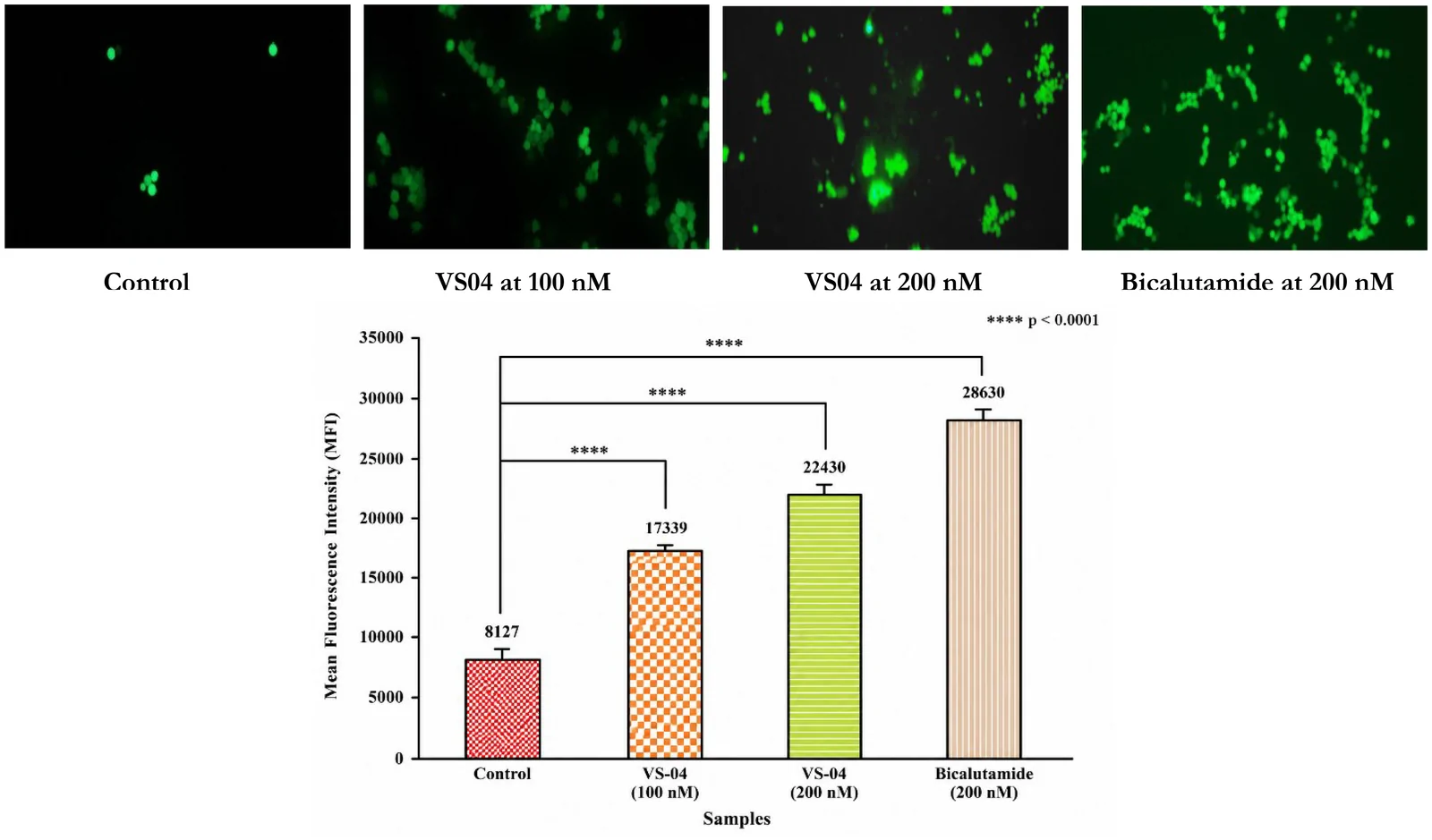
VS-04 induces intracellular reactive oxygen species (ROS) generation in LNCaP cells. Representative DCFDA fluorescence microscopy images of Control, VS-04 (100 nM), VS-04 (200 nM), and Bicalutamide (200 nM) demonstrate a concentration-dependent increase in intracellular ROS following VS-04 treatment. Quantitative analysis of mean fluorescence intensity (MFI) confirmed significantly elevated ROS levels in treated cells compared with the untreated control (8127.33 ± 41.53, 17339.33 ± 54.78, 22430.33 ± 114.07, and 28630.00 ± 65.57, respectively). Data are presented as the mean ± SD (n = 3). Statistical significance was determined by one-way ANOVA followed by Tukey’s multiple comparison test (****p < 0.0001 vs. Control).

The fluorescence microscopy images further confirmed the flow cytometric findings by providing qualitative evidence of ROS generation following VS-04 treatment (**Figure 4B**). Untreated control cells displayed only faint green DCFDA fluorescence, reflecting low basal oxidative stress. In contrast, treatment with VS-04 (100 nM) produced a noticeable increase in intracellular fluorescence, whereas VS-04 (200 nM) induced a more intense fluorescent signal, indicating increased ROS accumulation. The strongest fluorescence was observed in cells treated with bicalutamide (200 nM), confirming its potent ROS-inducing activity. The progressive increase in fluorescence intensity across the treatment groups visually demonstrated that VS-04 induced intracellular oxidative stress in a concentration-dependent manner.

Quantitative analysis of the mean fluorescence intensity (MFI) further supported these observations. The mean MFI increased significantly from 8127.33 ± 41.53 in untreated control cells to 17339.33 ± 54.78 following treatment with VS-04 (100 nM) and further increased to 22430.33 ± 114.07 after treatment with VS-04 (200 nM). The highest fluorescence intensity was observed in the bicalutamide (200 nM) group (28630.00 ± 65.57). Statistical analysis revealed that all the treatment groups presented significantly higher ROS levels than did the untreated control group (p < 0.0001). The fluorescence microscopy, flow cytometric analysis, and quantitative MFI measurements consistently demonstrated that VS-04 effectively elevated the intracellular ROS levels in LNCaP cells. These findings suggest that disruption of cellular redox homeostasis is a key component of the anticancer mechanism of VS-04 and provide a mechanistic basis for the subsequent induction of cell cycle arrest and apoptosis (**Figure 4**).

### 3.7 Apoptosis induction

The apoptotic effect of VS-04 was evaluated by Annexin V–FITC/PI flow-cytometric analysis and compared with the corresponding untreated control. As shown in **Table 3**, VS-04-treated sample, (B07) showed this clear drop in the living cell fraction, with only 51.73% of cells staying viable, while the control sample (D11) had 92.49% viability. For the VS-04-treated population itself, 14.13% of cells were put in the early apoptotic bucket and 33.70% were labeled late apoptotic, so the combined apoptotic population added up to 47.83%. Meanwhile, the untreated control had just 2.54% early apoptotic and 4.91% late apoptotic cells, giving a total apoptotic population of 7.45%. VS-04 treatment led to a strong rise in the entire apoptotic portion compared to the untreated group, and most of that shift seemed linked with the late-apoptotic subgroup. Also, the necrotic fraction stayed low in both groups, reaching 0.44% in the VS-04-treated cells and 0.06% in the control, which suggests the viable cell decrease was more tied to apoptosis rather than this kind of non- specific necrosis. In general, the Annexin V/PI results indicate that VS-04 strongly pushes apoptosis, taking the total apoptotic population from 7.45% in the control up to 47.83% after VS-04. That roughly 6.4-fold increase in apoptosis gives some experimental backing that apoptosis is an important intracellular process that underlies the antiproliferative effect of VS-04 (**Figure 5**).

**Table 3:** Effect of VS-04 on apoptotic cell populations.

Treatment	Live cells (%)	Early apoptosis (%)	Late apoptosis (%)	Total apoptosis (%)	Necrosis (%)
Control (D11)	92.49	2.54	4.91	7.45	0.06
VS-04 (B07)	51.73	14.13	33.70	47.83	0.44

Total apoptosis (%) = Early apoptosis (%) + Late apoptosis (%).

**Figure 5:**
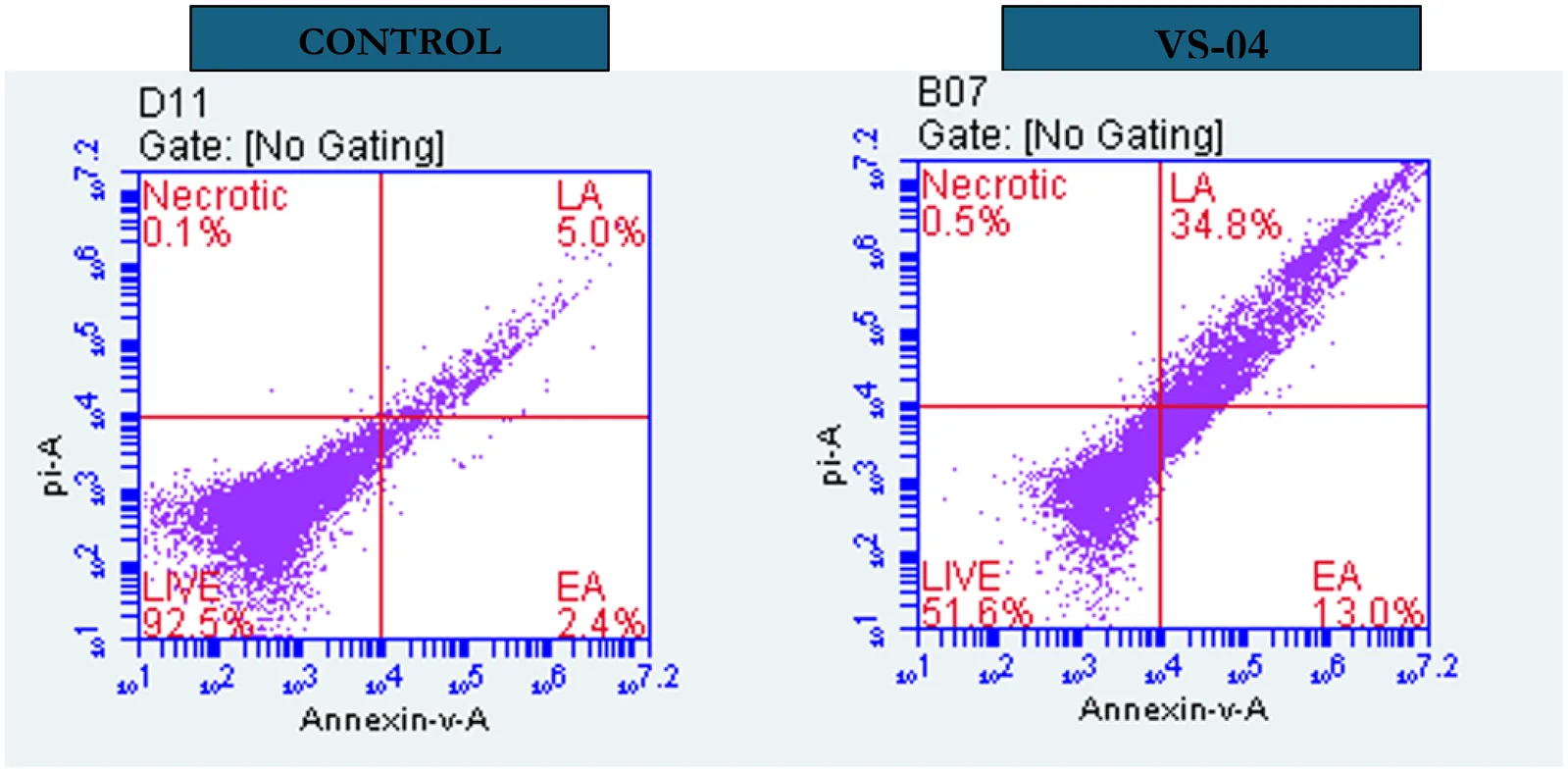
Annexin V–FITC/PI flow-cytometric analysis of apoptosis induced by VS-04 in prostate cancer.

### 3.8 Cell-cycle analysis

During cell-cycle analysis, treating the cells with VS-04 has shown alteration in cell-cycle distribution during flow-cytometric analysis while comparing it with untreated control. The control sample (D11) has highest cells distributed in G0/G1 phase (69.70%), where as 28.47% cells are in S phase. Only 1.83% of cells are present in M phase. Furthermore, treatment with VS-04 (B07) produced a drastic distribution change of cell population. This mark decreased in G0/G1 phase to 12.84% and the cells in S phase were seen increased which is up to 69.40%. This S phase increase shows the predominant cell-cycle population following VS-04 treatment. Another increase in mark was observed in M phase which is from 1.83% to 17.76% after VS-04 treatment (**Table 4**). Thus VS-04 treatment resulted in a pronounced depletion of G0/G1 population along with accumulation of cells predominantly in S phase and to a lesser extent, in the M phase. The S phase population increase has shown that VS-04 primarily interferes with normal cell cycle progression during DNA synthesis, which results in substantial accumulation of cells in S phase. The M phase shows concurrent increase which represents that the portion of treated cells progressed through DNA synthesis but subsequently accumulated at a later stage of the cell cycle. The markup distribution from G0/G1 toward S and M phase describes that VS-04 induces significant cell-cycle perturbation, with the predominant effect being S-phase accumulation, which may add to the antiproliferative activity observed for the synthesized compound. These observations associated with apoptotic nature and antiproliferative response of the VS-04 treatment supports the possibility of disruption of cell-cycle progression with cellular response to VS-04 (**Figure 6**).

**Table 4:** Effect of VS-04 treatment on cell-cycle distribution.

Treatment	G0/G1 phase (%)	S phase (%)	M phase (%)
Control (D11)	69.70	28.47	1.83
VS-04 (B07)	12.84	69.40	17.76

**Figure 6:**
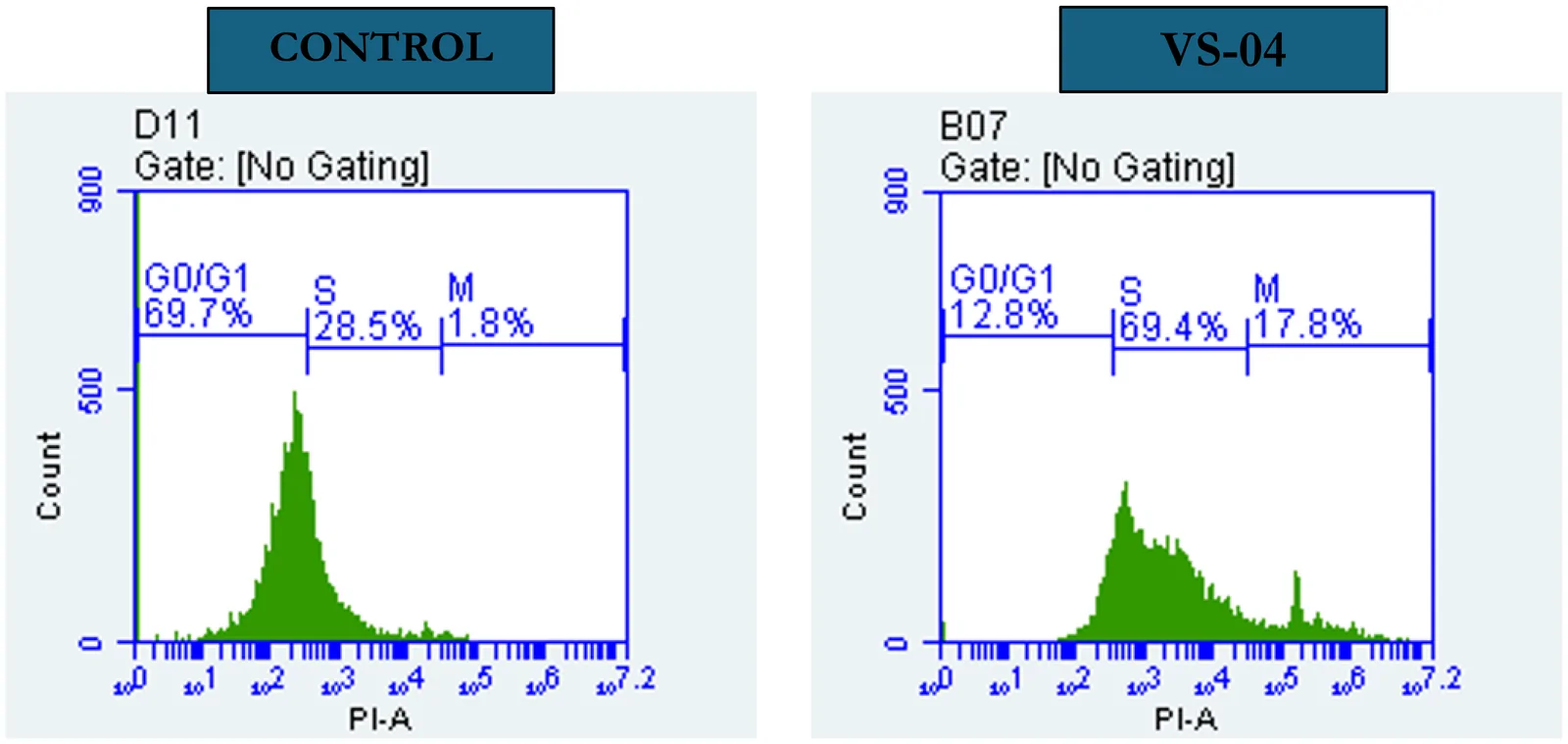
Flow-cytometric analysis of the cell-cycle distribution in control and VS-04 treated cells.

## 4 Discussion

Prostate cancer remains a major cause of cancer-related mortality, while resistance to androgen deprivation therapy continues to compromise the long-term efficacy of current treatment strategies. In this context, simultaneous modulation of androgen receptor (AR) signaling and complementary cellular vulnerabilities represents a rational approach for the development of new therapeutic candidates. In the present study, a series of oxadiazole–pyrazole hybrids was designed, synthesized, and evaluated through an integrated computational and biological approach. Among the synthesized derivatives, VS-04 emerged as the lead compound, exhibiting potent antiproliferative activity against both PC-3 (394.95 ± 3.03 nM) and LNCaP (464.85 ± 4.29 nM) cells. Molecular docking indicated that VS-04 was favorably accommodated within the AR ligand-binding pocket through a combination of non-covalent interactions, including hydrogen bonding, π-mediated contacts, hydrophobic interactions, and van der Waals forces. Consistent with these computational observations, flow-cytometric analysis demonstrated a concentration-dependent reduction in AR expression following VS-04 treatment, supporting the involvement of AR signaling modulation in its antiproliferative activity.

To further assess the relationship between predicted AR-binding affinity and cellular antiproliferative activity, docking scores were correlated with the corresponding IC_50_ values of VS-01–VS-20 against PC-3 and LNCaP cells. A moderate and statistically significant inverse correlation was observed between docking score and PC-3 IC_50_ values, indicating that more favorable docking scores were generally associated with lower IC_50_ values in PC-3 cells. In contrast, no significant correlation was observed between docking score and LNCaP IC_50_ values. Spearman’s rank analysis showed a comparable trend, with a significant association for PC-3 (ρ = −0.475, p = 0.035) but not for LNCaP cells (ρ = −0.027, p = 0.910). These findings suggest that AR binding may contribute to the antiproliferative activity of the compounds, particularly in PC-3 cells, although docking affinity alone does not account for the complete cellular response. The absence of a significant correlation in LNCaP cells further indicates that cellular potency is influenced by additional determinants, including intracellular exposure, membrane permeability, target dependence, and downstream signaling. Accordingly, molecular docking was considered supportive of the biological findings rather than a standalone predictor of cellular potency.

The biological effects of VS-04 were further associated with disruption of intracellular redox homeostasis. Treatment with VS-04 produced a concentration-dependent increase in intracellular ROS levels, as demonstrated by flow cytometry and DCFDA fluorescence microscopy. The elevation of ROS suggests that VS-04 perturbs the redox balance of prostate cancer cells, potentially increasing oxidative burden beyond the capacity of endogenous antioxidant defenses. Such redox imbalance can compromise cellular homeostasis and promote downstream events associated with loss of viability. The concurrent reduction in AR expression and elevation of ROS therefore suggest that the antiproliferative activity of VS-04 may involve complementary modulation of AR signaling and oxidative stress. This biological profile is consistent with the molecular-hybridization strategy employed to combine 1,3,4-oxadiazole and pyrazole pharmacophores within a common scaffold.

The predicted ADMET profile of VS-04 indicated a molecular weight of 449.514 Da, high GI absorption, and a consensus LogP of 5.965, reflecting its relatively lipophilic nature. The compound was predicted to be non-BBB permeant, with one Lipinski violation and a bioavailability score of 0.55. Regarding toxicity, VS-04 showed no predicted hERG I inhibition but was predicted to inhibit hERG II and exhibit hepatotoxicity, highlighting potential liabilities that require experimental validation. The predicted ADMET profile, together with its potent antiproliferative and mechanistic activity, supports VS-04 as a promising lead while indicating areas requiring further optimization and safety assessment.

The mechanistic relevance of these observations was further supported by the effects of VS-04 on cell-cycle progression and apoptotic cell death. Flow-cytometric cell-cycle analysis demonstrated a pronounced redistribution of the cellular population following VS-04 treatment. The G0/G1 population decreased from 69.70% in control cells to 12.84%, whereas the S-phase population increased from 28.47% to 69.40%, accompanied by an increase in the G2/M population from 1.83% to 17.76%. The marked enrichment of cells in the S phase indicates substantial perturbation of normal cell-cycle progression, with S-phase accumulation representing the predominant response to VS-04 exposure. In parallel, Annexin V-FITC/PI analysis demonstrated a pronounced increase in apoptosis. The total apoptotic population increased from 7.45% in untreated cells to 47.83% following VS-04 treatment, predominantly owing to an increase in late apoptosis from 4.91% to 33.70%. The necrotic fraction remained low in both groups (0.06% in control versus 0.44% following treatment), supporting apoptosis as the predominant form of cell death detected under the experimental conditions. Collectively, these findings indicate that VS-04 associated loss of cellular viability is accompanied by substantial cell-cycle perturbation and apoptotic induction. The SAR analysis further highlighted the importance of substitution pattern within the oxadiazole-pyrazole framework. VS-04, bearing a 4-CF_3_ substituent at R¹ and an unsubstituted R² position, displayed a favorable overall antiproliferative profile across both prostate cancer cell models. The differential activity observed among the remaining analogues indicated that modification at R¹ did not exert a uniform effect and was dependent on the chemical environment imposed by R². This context-dependent behavior suggests that electronic, steric, and lipophilic properties collectively influence biological activity and receptor recognition within the series. The combination of favorable AR interactions and potent cellular activity observed for VS-04 may therefore reflect an appropriate balance of these structural properties. Nevertheless, the distinct activity profiles observed between PC-3 and LNCaP cells, together with the weak docking–IC_50_ relationship in LNCaP cells, indicate that antiproliferative potency cannot be attributed exclusively to AR binding and may additionally depend on cellular uptake, intracellular distribution, target dependence, and downstream signaling mechanisms.

Combining all the findings support a complex cellular response to VS-04 involving AR modulation, oxidative stress, cell-cycle perturbation, and apoptosis. The concentration-dependent reduction in AR expression and elevation of ROS, together with S-phase accumulation and enhanced Annexin V-positive populations, provide convergent evidence that VS-04 affects multiple biological processes associated with prostate cancer cell survival. However, these observations should be interpreted as mechanistically associated events rather than evidence of a definitive linear signaling cascade, because direct causal relationships between AR modulation, ROS generation, cell-cycle disruption, and apoptosis were not established in the present study. Further target-validation experiments, including evaluation of AR-regulated signaling, apoptosis-associated proteins, cell-cycle regulatory proteins, mitochondrial function, and ROS-dependent rescue experiments, would be valuable for defining the precise molecular sequence underlying VS-04 activity.

the integration of molecular docking, docking–activity correlation, chemical synthesis, SAR analysis, antiproliferative evaluation, AR-expression analysis, ROS assessment, cell-cycle profiling, and apoptosis analysis provides a comprehensive characterization of VS-04. The combined computational and experimental findings identify VS-04 as a promising oxadiazole–pyrazole lead whose antiproliferative activity is associated with AR modulation and oxidative stress and is accompanied by pronounced S-phase accumulation and apoptotic cell death. These findings provide a rational foundation for further structural optimization and mechanistic validation of this scaffold as a potential lead for prostate cancer drug discovery.

### 4.1 Limitations and future directions

Although VS-04 demonstrated promising anticancer activity, several limitations remain. The biological evaluation was restricted to *in vitro* prostate cancer models, and therefore its pharmacokinetic behavior, systemic safety, and *in vivo* therapeutic efficacy remain to be established. Although AR modulation, ROS generation, S-phase accumulation, and apoptosis were observed, their direct causal relationships were not established. Molecular docking provided structural insight into AR–ligand interactions but cannot fully account for cellular permeability, intracellular exposure, metabolism, or downstream signaling. The predicted ADMET profile also requires experimental validation, particularly regarding the predicted hERG II inhibition and hepatotoxicity. Future studies should therefore focus on target-validation and pathway-specific investigations, including AR-regulated signaling, apoptosis- and cell-cycle-associated proteins, mitochondrial function, and oxidative stress pathways. *In vivo* efficacy, pharmacokinetic, and toxicity studies, together with further structural optimization guided by the observed SAR and ADMET profiles, will be important for determining the therapeutic potential of VS-04.

## 5 Conclusion

In the present study, a series of oxadiazole–pyrazole derivatives was designed, synthesized, and systematically evaluated using an integrated computational and experimental approach to identify potential anti-prostate cancer agents. Among the synthesized compounds, VS-04 emerged as the lead molecule, demonstrating potent antiproliferative activity against both androgen-dependent LNCaP and androgen-independent PC-3 cells, together with favorable AR-binding interactions and an overall supportive in silico ADMET profile. Molecular docking and docking–activity correlation analysis provided evidence for a contribution of AR binding to the observed cellular activity, while the lack of a strong correlation in LNCaP cells indicated that factors beyond receptor affinity also contribute to cellular potency.

Mechanistic evaluation further demonstrated that VS-04 reduced AR expression and increased intracellular ROS levels in a concentration-dependent manner. In addition, VS-04 induced pronounced S-phase accumulation and cell-cycle perturbation, accompanied by a substantial increase in Annexin V-positive apoptotic cells, indicating that its antiproliferative effect is associated with both cell-cycle dysregulation and apoptotic cell death. The combined findings support a complex biological response involving AR modulation, oxidative stress, cell-cycle perturbation, and apoptosis, although the causal relationship among these events requires further validation. The integration of molecular docking, SAR analysis, antiproliferative evaluation, AR-expression analysis, ROS assessment, cell-cycle profiling, apoptosis studies, and ADMET prediction provides a comprehensive preclinical characterization of VS-04. These findings establish VS-04 as a promising oxadiazole–pyrazole lead scaffold and provide a rational basis for further structural optimization, target-validation studies, pharmacokinetic and toxicity evaluation, and *in vivo* investigation toward the development of potential prostate cancer therapeutics.

## Data Availability

The original contributions presented in the study are included in the article/**Supplementary Material**. Further inquiries can be directed to the corresponding author.
