# Supplementary material for: Preclinical identification of an oxadiazole-pyrazole lead targeting prostate cancer through integrated mechanistic evaluation
Source: Front Oncol. 2026 Sep 21;16:1953079. doi: 10.3389/fonc.2026.1953079 (PMC13636019; doi:10.3389/fonc.2026.1953079)
Supplement: Supplementary file 1 [file DataSheet1.pdf]

Preclinical Identification of an *Oxadiazole-Pyrazole* Lead Targeting Prostate Cancer through Integrated Mechanistic Evaluation

Vanktesh Kumar[1*], Pankaj Wadhwa[2] Shubham Kumar[1*]

[1]*Department of Pharmaceutical Chemistry, School of Pharmaceutical Sciences, Lovely Professional University, Phagwara, Punjab, India* 144411

[2]*Department of Pharmaceutical Chemistry, Ummeed Institute of Pharmaceutical Sciences, Sri ganganagar, Rajasthan 333501, India*

***Corresponding Author:** Shubham Kumar, PhD (Shubhamm771@gmail.com)

SUPPLEMENTARY INFORMATION

Supplementary Methods

General Synthetic Procedure

The target oxadiazole–pyrazole derivatives (VS01–VS20) were synthesized through a multistep synthetic strategy (Scheme S1). Initially, substituted aromatic hydrazines were condensed with substituted acetophenones in ethanol containing a catalytic amount of glacial acetic acid under reflux for 3-4 h to afford the corresponding hydrazone intermediates. Cyclization of the hydrazones was subsequently carried out using phosphorus oxychloride ($POCl_3$) in dimethylformamide (DMF) under reflux at 80°C for 8 h to obtain the corresponding pyrazole aldehyde intermediates.

In parallel, substituted acid hydrazides reacted with cyanogen bromide (CNBr) in methanol under reflux at 70°C to synthesize the corresponding 2-amino-1,3,4-oxadiazole derivatives. The final oxadiazole-pyrazole hybrids (VS01–VS30) were obtained through Schiff base condensation between the pyrazole aldehydes and the amino-substituted oxadiazoles under optimized reaction conditions. The crude products were purified by recrystallization or column chromatography and characterized using FT-IR, ^1H NMR, ^13C NMR, and HRMS. The detailed synthetic procedures, purification methods, and spectroscopic characterization data for all compounds are provided in the Supplementary Information **(Scheme S1).**

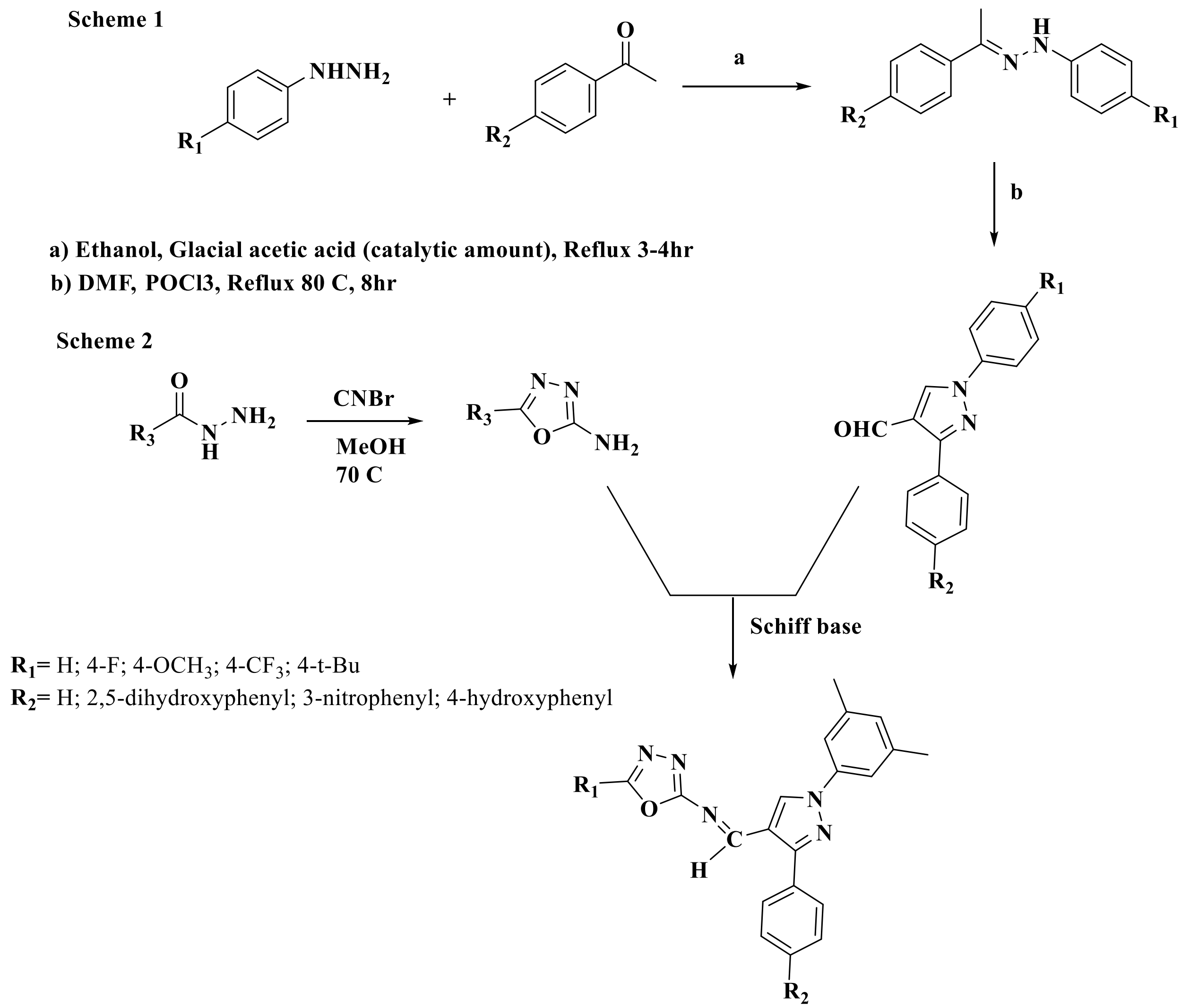

Scheme S1: Synthetic scheme for synthesizing compounds (VS01-VS20).

Spectroscopic data

1-(1-(3,5-dimethylphenyl)-3-phenyl-1H-pyrazol-4-yl)-N-(5-phenyl-1,3,4-oxadiazol-2-yl)methanimine **(VS01).** Yield: 72%; m.p.: 255–257 °C; yellow crystalline solid; Rf = 0.72 (ethyl acetate: hexane, 3:7); ^{1}H NMR (600 MHz, CDCL$_3$) δ 9.42 (s, 1H, CH=N), 8.35 (s, 1H, Ar-H), 7.70-7.66 (d, 2H, Ar-H), 7.51-7.55 (m, 4H, Ar-H), 7.40-7.37 (m 2H, Ar-H), 7.34-7.33 (d 1H, Ar-H), 6.87 (s, 1H, Ar-H), 2.25 (s, 6H, 2 × CH$_3$); **^{13}C NMR** (100 MHz, CDCl$_3$) δ 168.31, 161.33, 159.34, 150.20, 138.51, 138.33, 134.75 (d, J = 7.7 Hz), 131.83, 129.52, 129.10, 128.93, 128.55, 127.81, 127.23, 125.26, 120.18, 117.05, 21.16; **(ESI)** calc **m/z [M+H]$^+$** found 420.8011.

1-(1-(3,5-dimethylphenyl)-3-phenyl-1H-pyrazol-4-yl)-N-(5-(4-fluorophenyl)-1,3,4-oxadiazol-2-yl)methanimine **(VS02)** Yield: 59%; m.p.: 275-278 °C; Brownish solid; Rf = 0.81 (ethyl acetate: hexane, 3:7); ^{1}H NMR (600 MHz, CDCL$_3$) δ 9.34 (s, 1H CH=N), 8.35 (s, 1H Ar-H), 8.12 (dd, *J* = 8.5, 3.7 Hz, 2H Ar-H), 7.69 (dd, *J* = 8.6, 1.4 Hz, 2H Ar-H), 7.56–7.47 (m, 2H Ar-H), 7.43-7.25 (m, 5H Ar-H), 6.87 (t, *J* = 2.3 Hz, 1H Ar-H), 2.25 (s, 6H, 2 × CH$_3$) ; **^{13}C NMR** (100 MHz, CDCl$_3$) δ 168.31, 166.05, 164.41, 161.28, 159.34, 150.20, 138.51, 138.33, 134.75 (d, J = 7.7 Hz), 129.71–129.44 (m), 129.10, 128.55, 127.81, 120.51 (d, J = 3.7 Hz), 120.18, 117.05, 116.12, 115.97, 21.16; **(ESI)** calc. **m/z [M+H]$^+$** found 438.1257.

1-(1-(3,5-dimethylphenyl)-3-phenyl-1H-pyrazol-4-yl)-N-(5-(4-methoxyphenyl)-1,3,4-oxadiazol-2-yl)methanimine **(VS03).** Yield: 77; m.p.: 250-252; Yellowish brown solid; *R*f = 0.77 (ethyl acetate: hexane, 3:7); ^{1}H NMR (600 MHz, $CDCL_3$) δ 9.34 (s, 1H, CH=N), 8.35 (s, 1H, Ar-H), 7.90–7.87 (m, 2H, Ar-H), 7.71–7.67 (m, 2H, Ar-H), 7.54–7.49 (m, 2H, Ar-H), 7.41-7.33 (m, 6H, Ar-H), 7.00–6.97 (m, 2H, Ar-H), 6.87 (s, 1H, Ar-H), 3.80 (s, 3H, OCH_3), 2.25 (s, 6H, 2 xCH_3); **^{13}C NMR** (100 MHz, $CDCl_3$) δ 168.32, 162.83, 161.04, 159.34, 150.20, 138.42 (d, *J* = 17.7 Hz), 134.75 (d, *J* = 5.2 Hz), 129.52, 129.10, 128.55, 127.81, 127.29, 120.18, 118.30, 117.05, 114.89, 55.34, 21.16; **(ESI) *m*/*z*** calc **[M+H]$^+$** found 450.1986.

1-(1-(3,5-Dimethylphenyl)-3-phenyl-1H-pyrazol-4-yl)-N-(5-(4-(trifluoromethyl)phenyl)-1,3,4-oxadiazol-2-yl)methanimine **(VS04).** Yield: **88%**; m.p.: **270–272 °C**; deep yellow solid; *R*f: **0.69** (ethyl acetate:hexane, 3:7); **^{1}H NMR** (600 MHz, $CDCL_3$) δ 9.45 (s, 1H, CH=N), 8.35 (s, 1H, Ar-H), 8.11-8.08 (m, 2H, Ar-H), 7.79-7.76 (m, 2H, Ar-H), 7.70-7.68 (m, 2H, Ar-H), 7.54-7.50 (m, 2H, Ar-H), 7.40-7.33 (m, 6H, Ar-H), 6.87 (s, 1H, Ar-H), 2.25 (s, 6H, 2 xCH_3); **^{13}C** NMR (100 MHz, $CDCl_3$) δ 168.31, 160.90, 159.34, 150.20, 138.42 (d, *J* = 17.7 Hz), 134.75 (d, *J* = 5.2 Hz), 131.94, 131.61, 129.52, 129.10, 128.55, 128.10-127.71 (m), 127.09, 126.21 (q, *J* = 4.7 Hz), 125.22, 122.50, 120.18, 117.05, 21.16; **(ESI)** *m*/*z* calcd **[M+H]$^+$** 487.1698, found 488.2210.

N-(5-(4-(tert-butyl)phenyl)-1,3,4-oxadiazol-2-yl)-1-(1-(3,5-dimethylphenyl)-3-phenyl-1H-pyrazol-4-yl)methanimine **(VS05).** Yield: **78%**; m.p.: **300–302 °C**; off-white solid; *R*f: **0.77** (ethyl acetate, 3:7); **^{1}H NMR** (600 MHz, $CDCL_3$) δ 9.77 (s, 1H, CH=N), 8.35 (s, 1H, Ar-H), 7.98-7.95 (m, 2H, Ar-H), 7.71-7.67 (m, 2H, Ar-H), 7.54-7.50 (m, 2H, Ar-H), 7.44–7.33 (m, 8H, Ar-H), 6.87 (s, 1H, Ar-H), 2.05 (d, 6H, 2 x CH_3), 2.04 (s, 9H, *tert*-butyl); **^{13}C NMR** (100 MHz, $CDCl_3$) δ 168.31, 160.81, 159.34, 155.36, 150.20, 138.42 (d, *J* = 17.7 Hz), 134.75 (d, *J* = 5.2 Hz), 129.52, 129.10, 128.55, 127.81, 127.41, 126.00, 123.62, 120.18, 117.05, 34.89, 31.18, 21.16; **(ESI)** *m*/*z* calcd **[M+H]$^+$** found 476.2499.

2-(1-(3,5-Dimethylphenyl)-4-(((5-phenyl-1,3,4-oxadiazol-2-yl)imino)methyl)-1H-pyrazol-3-yl)benzene-1,4-diol **(VS06).** Yield: **55%**; m.p.: **290-292 °C**; **dark brownish-red solid**; *R*f: **0.74** (ethyl acetate, 3:7); **^{1}H NMR** (600 MHz, $CDCL_3$) δ 11.75 (s, 1H, OH), 9.40 (s, 1H, CH=N), 8.72 (s, 1H, H), 8.30 (s, 1H, OH), 8.07-8.03 (m, 2H, Ar-H), 7.56-7.51 (m, 2H, Ar-H), 7.45-7.34 (m, 5H, Ar-H), 7.05 (d, *J* ≈ 8.0 Hz, 1H, Ar-H), 6.88-6.85 (m, 1H, Ar-H), 6.74-6.71 (m, 1H, Ar-H), 2.25 (s, 6H, 2 x CH_3); **^{13}C NMR** (100 MHz, $CDCl_3$) δ 168.19, 161.33, 158.99, 150.17, 149.64, 148.45, 138.66, 138.33, 135.07, 131.83, 128.93, 127.81, 127.23, 125.26, 121.84, 120.10, 119.78, 119.36, 116.78 (d, *J* = 2.3 Hz), 21.16; **(ESI)** *m*/*z* calcd for [M+H]$^+$ found **452.1688**.

2-(1-(3,5-Dimethylphenyl)-4-(((5-(4-fluorophenyl)-1,3,4-oxadiazol-2-yl)imino)methyl)-1H-pyrazol-3-yl)benzene-1,4-diol **(VS07).** Yield: **66%**; m.p.: **330-332 °C**; dark brownish-black solid; *R*f: **0.68** (ethyl acetate:hexane, 3:7); **^{1}H NMR** (600 MHz, $CDCL_3$) δ 11.47 (s, 1H, OH), 9.43 (s, 1H, CH=N), 9.35 (s, 1H, H), 8.58 (s, 1H, OH), 8.13-8.11 (m, 2H, Ar-H), 7.34-7.28 (m, 2H, Ar-H), 7.05 (d, *J* ≈ 8.0 Hz, 1H, Ar-H), 6.87-6.86 (m, 1H, Ar-H), 6.73-6.71 (m, 1H, Ar-H), 2.25 (s, 6H, 2 x CH_3); **^{13}C NMR** (100 MHz, $CDCl_3$) δ 168.20, 166.47, 164.00, 161.28, 158.99, 150.17, 149.64, 148.45, 138.66, 138.33, 135.07, 129.60 (d, *J* = 8.9 Hz), 127.81, 121.84, 120.51 (d, *J* = 3.7 Hz), 120.10, 119.78, 119.36, 116.78 (d, *J* = 2.3 Hz), 116.16, 115.93, 21.16; **(ESI)** *m*/*z* calcd for [M+H]$^+$ found 470.1597.

2-(1-(3,5-Dimethylphenyl)-4-(((5-(4-methoxyphenyl)-1,3,4-oxadiazol-2-yl)imino)methyl)-1H-pyrazol-3-yl)benzene-1,4-diol **(VS08).** Yield: **82%**; m.p.: **317–320 °C**; dark brownish-black solid; *R*f: **0.70** (ethyl acetate:hexane, 3:7); **^{1}H NMR** (600 MHz, $CDCL_3$) δ 11.73 (s, 1H, OH), 9.67 (s, 1H, CH=N), 8.72 (s, 1H, H), 8.30 (s, 1H, OH), 7.89–7.87 (m, 2H, Ar-H), 7.34 (d, *J* ≈ 8.2 Hz, 2H, Ar-H), 7.05 (d, *J* ≈ 8.1 Hz, 1H, Ar-H), 6.99-6.97 (m, 2H, Ar-H), 6.87–6.86 (m, 1H, Ar-H), 6.73–6.71 (m, 1H, Ar-H), 3.80 (s, 3H, OCH_3), 2.25 (s, 6H, 2 xCH_3); **^{13}C NMR** (100 MHz, $CDCl_3$) δ 168.09, 162.83, 161.04, 158.99, 150.17, 149.64, 148.45, 138.66, 138.33, 135.07, 127.81, 127.29, 121.84, 120.10, 119.78, 119.36, 118.30, 116.78 (d, *J* = 2.3 Hz), 114.89, 55.34, 21.16; **(ESI)** *m*/*z* calcd **[M+H]$^+$** found 482.1822.

2-(1-(3,5-Dimethylphenyl)-4-(((5-(4-(trifluoromethyl)phenyl)-1,3,4-oxadiazol-2-yl)imino)methyl)-1H-pyrazol-3-yl)benzene-1,4-diol **(VS09).** Yield: **87%**; m.p.: **308-310 °C**; **dark brownish-black solid**; *R*f: **0.75** (ethyl acetate:hexane, 3:7); **^{1}H NMR** (600 MHz, $CDCL_3$) δ 11.06 (s, 1H, OH), 9.20 (s, 1H, CH=N), 8.84 (s, 1H, OH), 8.30-8.08 (m, 2H, Ar-H), 7.79-7.76 (m, 2H, Ar-H), 7.34 (d, *J* ≈ 8.2 Hz, 2H, Ar-H), 7.05 (d, *J* ≈ 8.0 Hz, 1H, Ar-H), 6.87-6.86 (m, 1H, Ar-H), 6.73-6.71 (m, 1H, Ar-H), 2.25 (s, 6H, 2 xCH_3); **^{13}C NMR** (100 MHz, $CDCl_3$) δ 168.19, 160.90, 158.99, 150.17, 149.64, 148.45, 138.66, 138.33, 135.07, 131.94, 131.61, 128.10-127.71 (m), 127.09, 126.21 (q, *J* = 4.7 Hz), 125.22, 122.50, 121.84, 120.10, 119.78, 119.36, 116.78 (d, *J* = 2.3 Hz), 21.16; **(ESI)** *m*/*z* calcd **$[M+H]^+$** found 520.1607.

2-(1-(3,5-Dimethylphenyl)-4-(((5-(4-(tert-butyl)phenyl)-1,3,4-oxadiazol-2-yl)imino)methyl)-1H-pyrazol-3-yl)benzene-1,4-diol **(VS10).** Yield: **71%**; m.p.: **305–307 °C**; dark brownish-red solid; *R*f: **0.68** (ethyl acetate:hexane, 3:7); **^{1}H NMR** (600 MHz, $CDCL_3$) δ 12.15 (s, 1H, OH), 9.20 (s, 1H, CH=N), 8.67 (s, 1H, OH), 8.43 (s, 1H, Ar-H), 8.00–7.99 (m, 2H, Ar-H), 7.86–7.80 (m, 2H, Ar-H), 7.17–7.16 (m, 2H, Ar-H), 7.03-6.99 (m, 1H, Ar-H), 6.90–6.89 (m, 1H, Ar-H), 6.82–6.70 (m, 2H, Ar-H), 2.23 (s, 9H, *tert*-butyl); **^{13}C NMR** (100 MHz, $CDCl_3$) δ 168.19, 160.81, 158.99, 155.36, 150.17, 149.64, 148.45, 138.66, 138.33, 135.07, 127.81, 127.41, 126.00, 123.62, 121.84, 120.10, 119.78, 119.36, 116.78 (d, *J* = 2.3 Hz), 34.89, 31.18, 21.16; **(ESI)** *m*/*z* calcd $[M+H]^+$ found 508.2344.

1-(1-(3,5-Dimethylphenyl)-3-(3-nitrophenyl)-1H-pyrazol-4-yl)-N-(5-phenyl-1,3,4-oxadiazol-2-yl)methanimine **(VS11).** Yield: **66%**; m.p.: **245–247 °C**; light yellow solid; *R*f: **0.68** (ethyl acetate:hexane, 3:7); **^{1}H NMR** (600 MHz, $CDCL_3$) δ 9.36 (s, 1H, CH=N), 8.63 (s, 1H, Ar-H), 8.20–8.18 (m, 2H, Ar-H), 8.07–7.97 (m, 3H, Ar-H), 7.72–7.70 (m, 2H, Ar-H), 7.55–7.52 (m, 4H, Ar-H), 7.44-7.33 (m, 3H, Ar-H), 6.87 (s, 1H, Ar-H), 2.25 (s, 6H, 2 xCH_3); **^{13}C NMR** (100 MHz, $CDCl_3$) δ 168.31, 161.33, 159.07, 148.58, 147.44, 138.42 (d, *J* = 17.5 Hz), 135.54, 135.21, 134.87, 131.83, 129.92, 128.93, 127.81, 127.23, 125.97, 125.26, 124.24, 120.18, 118.55, 21.16; **(ESI)** *m*/*z* calcd **$[M+H]^+$** found 465.1670.

1-(1-(3,5-Dimethylphenyl)-3-(3-nitrophenyl)-1H-pyrazol-4-yl)-N-(5-(4-fluorophenyl)-1,3,4-oxadiazol-2-yl)methanimine **(VS12).** Yield: **85%**; m.p.: **240-242 °C**; light yellow solid; *R*f: **0.65** (ethyl acetate:hexane, 3:7); **^{1}H NMR** (600 MHz, $CDCL_3$) δ 9.78 (s, 1H, CH=N), 8.63 (s, 1H, Ar-H), 8.20–8.18 (m, 2H, Ar-H), 8.13–8.11 (m, 2H, Ar-H), 8.00–7.97 (m, 2H, Ar-H), 7.72–7.70 (m, 2H, Ar-H), 7.34–7.28 (m, 2H, Ar-H), 6.87 (s, 1H, Ar-H), 2.25 (s, 6H, 2 xCH_3); **^{13}C NMR** (100 MHz, $CDCl_3$) δ 168.31, 166.47, 164.00, 161.28, 159.07, 148.58, 147.44, 138.42 (d, *J* = 17.5 Hz), 135.54, 135.21, 134.87, 129.92, 129.60 (d, *J* = 8.9 Hz), 127.81, 125.97, 124.24, 120.51 (d, *J* = 3.7 Hz), 120.18, 118.55, 116.16, 115.93, 21.16; **(ESI)** *m*/*z* calcd **$[M+H]^+$** 482.1503, found 483.0981.

1-(1-(3,5-Dimethylphenyl)-3-(3-nitrophenyl)-1H-pyrazol-4-yl)-N-(5-(4-methoxyphenyl)-1,3,4-oxadiazol-2-yl)methanimine **(VS13).** Yield: **55%**; m.p.: **240-242 °C**; **off-white solid**; *R*f: **0.72** (ethyl acetate:hexane, 3:7); **^{1}H NMR** (600 MHz, $CDCL_3$) δ 9.69 (s, 1H, CH=N), 8.63 (s, 1H, Ar-H), 8.20–8.18 (m, 2H, Ar-H), 7.99-7.97 (m, 2H, Ar-H), 7.89-7.87 (m, 2H, Ar-H), 7.72–7.70 (m, 2H, Ar-H), 7.34 (d, *J* ≈ 8.2 Hz, 2H, Ar-H), 6.99-6.97 (m, 2H, Ar-H), 6.87 (s, 1H, Ar-H), 3.86 (s, 3H, OCH_3), 2.25 (s, 6H, 2 xCH_3); **^{13}C NMR** (100 MHz, $CDCl_3$) δ 168.32, 162.83, 161.04, 159.07, 148.58, 147.44, 138.42 (d, *J* = 17.5 Hz), 135.54, 135.21, 134.87, 129.92, 127.81, 127.29, 125.97, 124.24, 120.18, 118.55, 118.30, 114.89, 55.34, 21.16; **(ESI)** *m*/*z* calcd **$[M+H]^+$** found 495.1804.

1-(1-(3,5-Dimethylphenyl)-3-(3-nitrophenyl)-1H-pyrazol-4-yl)-N-(5-(4-(trifluoromethyl)phenyl)-1,3,4-oxadiazol-2-yl)methanimine **(VS14).** Yield: **79%**; m.p.: **272-274 °C**; off-white solid; *R*f: **0.76** (ethyl acetate:hexane, 3:7); **^{1}H NMR** (600 MHz, $CDCL_3$) δ 9.64 (s, 1H, CH=N), 8.63 (s, 1H, Ar-H), 8.20-8.18 (m, 2H, Ar-H), 8.11-8.08 (m, 2H, Ar-H), 7.99-7.97 (m, 2H, Ar-H), 7.79-7.76 (m, 2H, Ar-H), 7.72-7.70 (m, 2H, Ar-H), 7.34 (d, *J* ≈ 8.2 Hz, 2H, Ar-H), 6.87 (s, 1H, Ar-H), (s, 1H, H), 2.25 (s, 6H, 2 xCH_3); **^{13}C NMR** (100 MHz, $CDCl_3$) δ 168.31, 160.90, 159.07, 148.58, 147.44, 138.42 (d, *J* = 17.5 Hz), 135.54, 135.21, 134.87, 131.94, 131.61, 129.92,

128.10–127.71 (m), 127.09, 126.42–125.87 (m), 125.22, 124.24, 122.50, 120.18, 118.55, 21.16; **(ESI)** *m/z* calcd **[M+H]+** found 533.1693.

N-(5-(4-(tert-butyl)phenyl)-1,3,4-oxadiazol-2-yl)-1-(1-(3,5-dimethylphenyl)-3-(3-nitrophenyl)-1H-pyrazol-4-yl)methanimine **(VS15).** Yield: **84%**; m.p.: **265–268 °C**; **off-white solid**; *R*f: **0.81** (ethyl acetate:hexane, 3:7); **^{1}H NMR** (600 MHz, CDCL$_3$) δ 9.39 (s, 1H, CH=N), 8.63 (s, 1H, Ar-H), 8.20-8.18 (m, 2H, Ar-H), 7.99-7.96 (m, 2H, Ar-H), 7.72-7.70 (m, 2H, Ar-H), 7.44-7.42 (m, 2H, Ar-H), 7.34-7.33 (m, 2H, Ar-H), 6.87 (s, 1H, Ar-H), 2.30 (s, 9H, *tert*-butyl); **^{13}C NMR** (100 MHz, CDCl$_3$) δ 168.31, 160.81, 159.07, 155.36, 148.58, 147.44, 138.42 (d, *J* = 17.5 Hz), 135.54, 135.21, 134.87, 129.92, 127.81, 127.41, 125.99 (d, *J* = 3.0 Hz), 124.24, 123.62, 120.18, 118.55, 34.89, 31.18, 21.16; **(ESI)** *m/z* calcd **[M+H]+** found 521.1358.

4-(1-(3,5-Dimethylphenyl)-4-(((5-phenyl-1,3,4-oxadiazol-2-yl)imino)methyl)-1H-pyrazol-3-yl)phenol **(VS16).** Yield: **89%**; m.p.: **291-293 °C**; reddish-brown solid; *R*f: **0.75** (ethyl acetate:hexane, 3:7); **^{1}H NMR** (600 MHz, CDCL$_3$) δ 9.40 (s, 1H, CH=N), 8.35 (s, 1H, Ar-H), 8.06–8.04 (m, 2H, Ar-H), 7.67–7.65 (m, 2H, Ar-H), 7.55-7.52 (m, 2H, Ar-H), 7.44-7.41 (m, 4H, Ar-H), 7.34–7.33 (m, 2H, Ar-H), 6.87-6.85 (m, 2H, Ar-H), 5.26 (br s, 1H, OH), 2.16 (s, 6H, 2 xCH$_3$); **^{13}C NMR** (100 MHz, CDCl$_3$) δ 168.31, 161.33, 159.33, 159.12, 149.42, 138.42 (d, *J* = 17.7 Hz), 134.76, 131.83, 129.80, 129.01 (d, *J* = 16.3 Hz), 127.81, 127.23, 125.26, 120.18, 117.30, 116.09, 21.16; **(ESI)** *m/z* calcd **[M+H]+** found 436.1752

4-(1-(3,5-Dimethylphenyl)-4-(((5-(4-fluorophenyl)-1,3,4-oxadiazol-2-yl)imino)methyl)-1H-pyrazol-3-yl)phenol **(VS17). Yield: 59%; m.p.: 295–297 °C;** reddish-brown solid**; *R*f: 0.**68 (ethyl acetate: hexane, 3:7); **^{1}H NMR** (600 MHz, CDCL$_3$) δ 9.73 (s, 1H, CH=N), 8.35 (s, 1H, Ar-H), 8.13–8.11 (m, 2H, Ar-H), 7.67–7.65 (m, 2H, Ar-H), 7.34–7.28 (m, 5H, Ar-H), 6.87–6.85 (m, 2H, Ar-H), 4.73 (s, 1H, OH), 2.25 (s, 6H, 2 xCH$_3$); **^{13}C NMR** (100 MHz, CDCl$_3$) δ 168.31, 166.47, 164.00, 161.28, 159.33, 159.12, 149.42, 138.42 (d, *J* = 17.7 Hz), 134.76, 129.91–129.45 (m), 129.09, 127.81, 120.51 (d, *J* = 3.7 Hz), 120.18, 117.30, 116.26–115.82 (m), 21.16; **(ESI) *m/z* calc [M+H]+** found 454.1708.

4-(1-(3,5-Dimethylphenyl)-4-(((5-(4-methoxyphenyl)-1,3,4-oxadiazol-2-yl)imino)methyl)-1H-pyrazol-3-yl)phenol **(VS18).** Yield: **66%**; m.p.: **293–295 °C**; reddish-brown powder; *R*f: **0.77** (ethyl acetate:hexane, 3:7); **^{1}H NMR** (600 MHz, CDCL$_3$) δ 9.64 (s, 1H, CH=N), 8.35 (s, 1H, Ar-H), 7.89-7.87 (m, 2H, Ar-H), 7.67-7.65 (m, 2H, Ar-H), 7.34-7.28 (m, 2H, Ar-H), 6.99-6.97 (m, 2H, Ar-H), 6.87-6.85 (m, 3H, Ar-H), 5.02 (s, 1H, OH), 3.80 (s, 3H, OCH$_3$), 2.25 (s, 6H, 2 xCH$_3$); **^{13}C NMR** (100 MHz, CDCl$_3$) δ 168.32, 162.83, 161.04, 159.33, 159.12, 149.42, 138.42 (d, *J* = 17.7 Hz), 134.76, 129.80, 129.09, 127.81, 127.29, 120.18, 118.30, 117.30, 116.09, 114.89, 55.34, 21.16; **(ESI)** *m/z* calc **[M+H]+** found 466.1981.

4-(1-(3,5-Dimethylphenyl)-4-(((5-(4-(trifluoromethyl)phenyl)-1,3,4-oxadiazol-2-yl)imino)methyl)-1H-pyrazol-3-yl)phenol **(VS19).** Yield: **71%**; m.p.: **298–300 °C**; reddish-brown powder; *R*f: **0.74** (ethyl acetate:hexane, 3:7); **^{1}H NMR** (600 MHz, CDCL$_3$) δ 9.34 (s, 1H, CH=N), 8.35 (s, 1H, Ar-H), 8.14–8.05 (m, 2H, Ar-H), 7.78 (dq, *J* = 11.2, 1.5 Hz, 2H, Ar-H), 7.70–7.62 (m, 2H, Ar-H), 7.34 (d, *J* = 2.1 Hz, 2H, Ar-H), 6.90–6.82 (m, 3H, Ar-H); **^{13}C NMR** (100 MHz, CDCl$_3$) δ 168.31, 160.90, 159.33, 159.12, 149.42, 138.42 (d, *J* = 17.7 Hz), 134.76, 131.94, 131.61, 129.80, 129.09, 128.10–127.71 (m), 127.09, 126.21 (q, *J* = 4.7 Hz), 125.22, 122.50, 120.18, 117.30, 116.09, 21.**16**; **(ESI)** *m/z* calc **[M+H]+** found 504.1499

4-(4-(((5-(4-(tert-butyl)phenyl)-1,3,4-oxadiazol-2-yl)imino)methyl)-1-(3,5-dimethylphenyl)-1H-pyrazol-3-yl)phenol **(VS20).** Yield: **78%**; m.p.: **280-282 °C**; reddish-brown powder; *R*f: **0.67** (ethyl acetate:hexane, 3:7); **^{1}H NMR** (600 MHz, CDCL$_3$) δ 9.34 (s, 1H, CH=N), 8.35 (s, 1H, Ar-H), 7.99-7.94 (m, 2H, Ar-H), 7.69-7.63 (m, 2H, Ar-H), 7.45-7.40 (m, 2H, Ar-H), 7.34 (d, *J* = 2.2 Hz, 2H, Ar-H), 6.89–6.84 (m, 3H, Ar-H), 1.34 (s, 9H, *tert*-butyl); **^{13}C NMR** (100 MHz, CDCl$_3$) δ 168.31, 160.81, 159.33, 159.12, 155.36, 149.42, 138.51, 138.33, 134.76, 129.80, 129.09, 127.81, 127.41, 126.00, 123.62, 120.18, 117.30, 116.09, 34.89, 31.18, 21.16; **(ESI)** *m/z* calcd **[M+H]**+ found 492.2377.

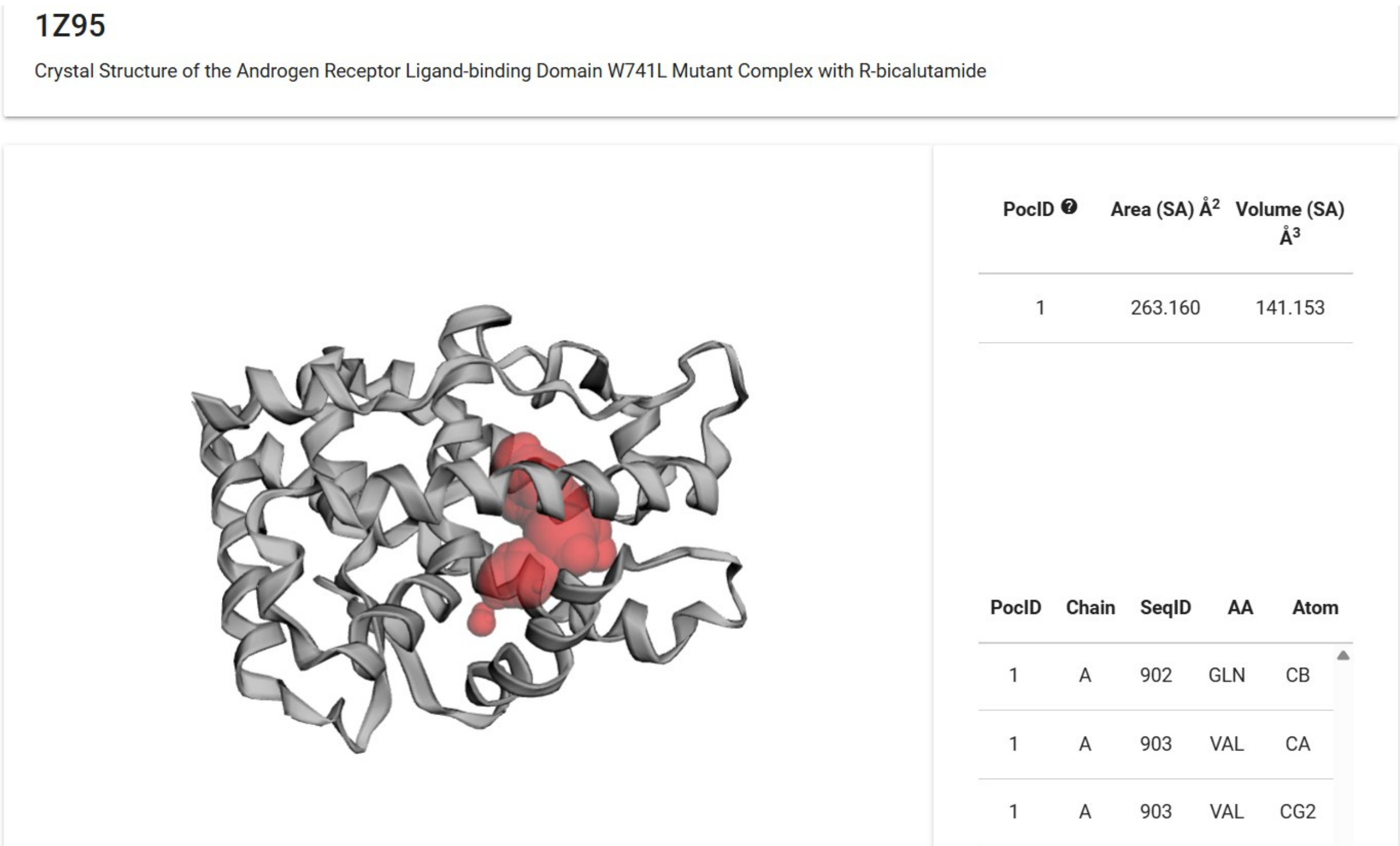

Figure S1: CASTp analysis of the androgen receptor ligand-binding domain (PDB ID: 1Z95). The identified binding pocket (Pocket 1) is highlighted in red and exhibits a surface area of 263.16 Å^2 and a volume of 141.15 Å^3. The corresponding pocket residues and atoms are shown in the accompanying CASTp output, supporting the identification of the ligand-binding region used for molecular docking.

Table S1: Comparison of CASTp-identified binding-pocket residues with docking results for the androgen receptor (PDB ID: 1Z95)

S. No.	Residue	CASTp pocket	Molecular docking protocol
1	Leu704	✓	✓
2	Leu707	✓	✓
3	Gly708	✓	✓
4	Leu741	✓	✓
5	Met742	✓	✓
6	Met745	✓	✓

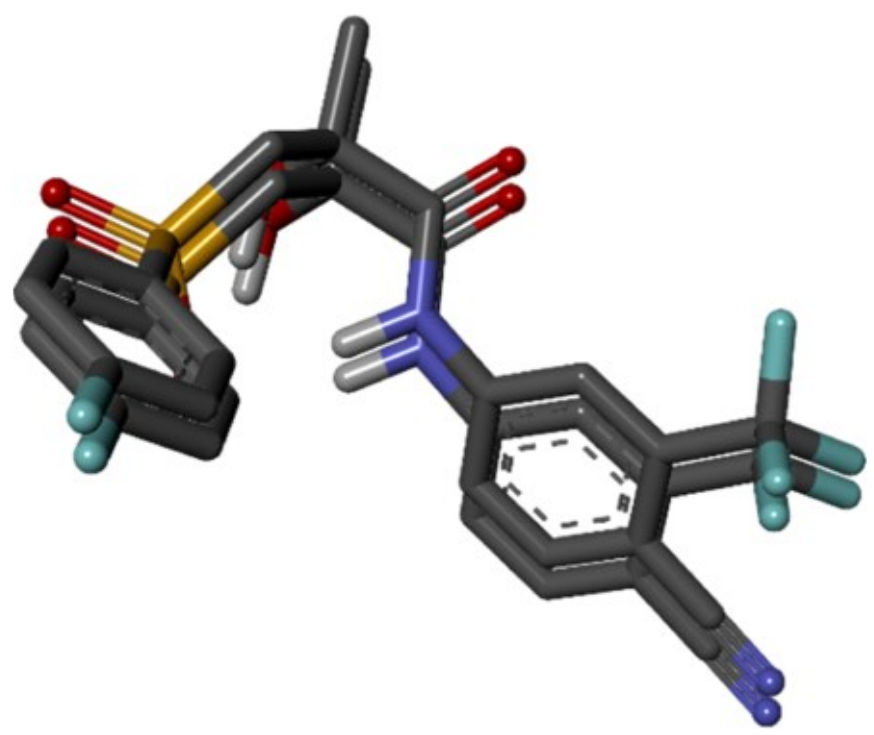

Figure S2 : Redocked and superimposed co-crystalline ligand of 1Z95.

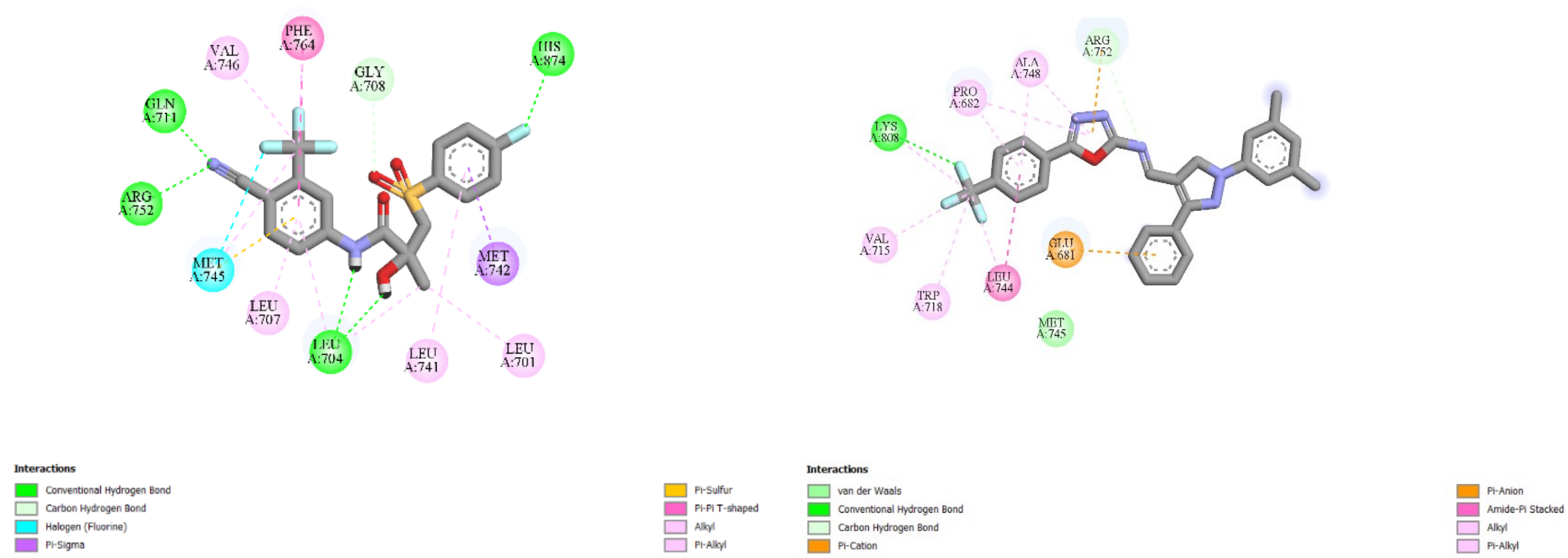

Figure S3: 2D interactions of VS04 **(right)** compared with Bicalutamide **(left).**

Table S2: Docking score and 2D interactions of the designed molecules along with Bicalutamide used as reference.

Molecule Names	Docking Score/Binding Affinity (Kcal/Mol)	H-Bond	Π–Π /T-Shaped	Π–Cation	Π–Σ	Alkyl / Π-Alkyl
**Bicalutamide**	**-10.8**	Arg A:752, Pro A:682, Glu A:681	Trp A:718	Lys A:808	-	Val A:715, Tyr A:763, Ala A:748, Pro A:766, Val A:684, Leu A:744
**VS-01**	**-10.7**	Arg A752, Glu A;681, Trp A:751	Trp A:718	-	Ala A:748, Val A:715	Val A:684, Leu A:805, Pro A:801, Pro A:682, Leu A:744, Lys A:808
**VS-02**	**10.9**	Tyr A:781	Phe A:876	Glu A:872	-	Arg A:779, His A:776

**VS-03**	**-8.8**	Arg A:871, Glu A:872	-	Lys A:883	-	Arg A:779, His A:776, Leu A:880
**VS-04**	**-11.1**	-	-	Arg A:752	Pro A:682, Thr A:755	Ala A:748, Val A:715, Leu A:805, Trp A:751, Pro A:801
**VS-05**	**-10.0**	-	-	Arg A:752	Pro A:682, Thr A:755	Ala A:748, Val A:715, Leu A:805, Trp A:751, Pro A:801
**VS-06**	**-10.3**	Arg A:752, Pro A:682, Glu A:681, Trp A:751	Trp A:718	Lys A:808	Val A:715, Ala A:748	Leu A:744, Val A:684, Leu A:806, Pro A:801
**VS-07**	**-10.2**	Asn A:756, Pro A:682	Trp A:718	Arg A:752, Lys A:808	-	Ala A:748, Leu A:744, Val A:715, Tyr A:763, Val A:684, Pro A:766
**VS-08**	**-9.4**	Asn A:756, Met A:745	Tyr A:751, Leu A:744	-	-	Ala A:748, Pro A:682, Leu A:805, Trp A:718, Lys A:808. Val A:715, Val A:684, Pro A: 801
**VS-09**	**-10.2**	-	Tyr A:915	Arg A:871, Glu A:872		Lys A:912, Pro A:913, Pro A:868
**VS-10**	**-8.4**	-	Tyr A:915	Arg A:871, Glu A:872	Glu A:875	Lys A:912, Pro A:913, Pro A:868
**VS-11**	**-10.6**	Arg A:752, Pro A:682, Glu A:681	TRP A:718	Lys A:808	Val A:715	Ala A:748, Tyr A:763, Pro A:766, Val A: 684, Leu A:744
**VS-12**	**-10.9**	Glu A:681,Pro A:682	Trp A:718	Arg A:752	-	Tyr A:763, Val A:684, Pro A:766, Ala A:748, Lys A:808, Leu A:744, Val A:715
**VS-13**	**-9.9**	Arg A:752	-	Glu A:681	Trp A:718	Val A:715, Lys A:808, Leu A:744, Pro A:682, Ala A:748
**VS-14**	**-9.0**	Lys A:808, Met A:745	Leu A:744	Glu A:681, Arg A:752	-	Ala A:748, Pro A:682, Val A:715, Trp A:718, Leu A:805
**VS-15**	**-9.9**	-	-	Arg A:752	Thr A:755	Ala A:748, Pro A:682, Val A:715, Leu A:805
**VS-16**	**-10.6**	Arg A:752, Glu A:681, Trp A: 751	Trp A:718	Lys A:808	Ala A:748, Val A:715	Leu A:744, Pro A:682, Leu A:805, Val A:684, Pro A:801
**VS-17**	**-10.9**	Arg A:752, Trp A:751, Glu A:681	Trp A:718	Lys A:808	Ala A:748, Val A:715	

**VS-18**	**-9.8**	Arg A:752	Trp A:751	-	Trp A:718	Ala A:748, Pro A:682, Leu A:744, Val A:715, Lys A:808
**VS-19**	**-9.5**	Lys A:808	Trp A:751	Arg A:752	-	Pro A:682, Ala A:748, Trp A:718, Val A:684, Leu A:744, Val A:715
**VS-20**	**-9.8**	Asn A:756, Trp A:751	-	Arg A:752	-	Ala A:748, Pro A:682, Val A:715, Lys A:808, Trp A:718, Leu A:744, Pro A:801, Val A:684

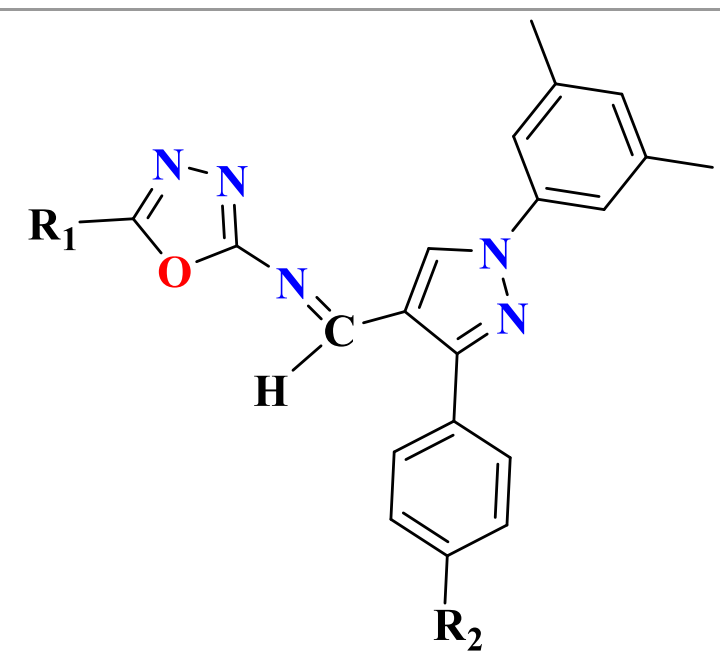

Table S3: IC_{50} of all synthesized compounds in two different cell lines.

S. No.	Compound	R₁	R₂	PC-3 IC_{50} ± SD (nM)	LNCaP IC_{50} ± SD (nM)
**1**	VS-01	H	H	909.75 ± 1.69	952.41 ± 2.41
**2**	VS-02	4-F	H	473.46 ± 2.77	1359.63 ± 1.79
**3**	VS-03	4-OCH_3	H	616.42 ± 2.30	1736.15 ± 1.41
**4**	**VS-04**	**CF_3**	**H**	**394.95** ± 3.03	**464.85** ± 4.29
**5**	VS-05	4-*tert*-butyl	H	635.87 ± 2.26	901.29 ± 2.58
**6**	VS-06	H	2,5-*dihydroxy*	561.2 ± 2.44	677.68 ± 3.33
**7**	VS-07	4-F	2,5-*dihydroxy*	810.63 ± 1.89	976.67 ± 2.40
**8**	VS-08	4-OCH_3	2,5-*dihydroxy*	587.56 ± 2.39	698.17 ± 3.26
**9**	VS-09	CF_3	2,5-*dihydroxy*	910.18 ± 1.71	752.35 ± 3.01
**10**	VS-10	4-*tert*-butyl	2,5-*dihydroxy*	580.02 ± 2.40	580.00 ± 3.70
**11**	VS-11	H	3-nitro	747.5 ± 2.02	795.88 ± 2.87
**12**	VS-12	4-F	3-nitro	854.62 ± 1.81	529.32 ± 4.01
**13**	VS-13	4-OCH_3	3-nitro	460.92 ± 2.82	1121.44 ± 2.15
**14**	VS-14	CF_3	3-nitro	769.63 ± 1.88	558.08 ± 3.86
**15**	VS-15	4-*tert*-butyl	3-nitro	749.65 ± 1.91	829.97 ± 2.78
**16**	VS-16	H	4-*hydroxy*	990.54 ± 1.53	691.29 ± 3.31
**17**	VS-17	4-F	4-*hydroxy*	1077.41 ± 1.42	1664.7 ± 1.49
**18**	VS-18	4-OCH_3	4-*hydroxy*	533.79 ± 2.47	962.34 ± 2.47
**19**	VS-19	CF_3	4-*hydroxy*	622.59 ± 2.24	784.25 ± 2.96
**20**	VS-20	4-*tert*-butyl	4-*hydroxy*	769.35 ± 1.90	575.51 ± 3.74
**Reference Drug**	**Bicalutamide**	**-**	**-**	**412.61** ± 3.05	**118.31** ± 4.54

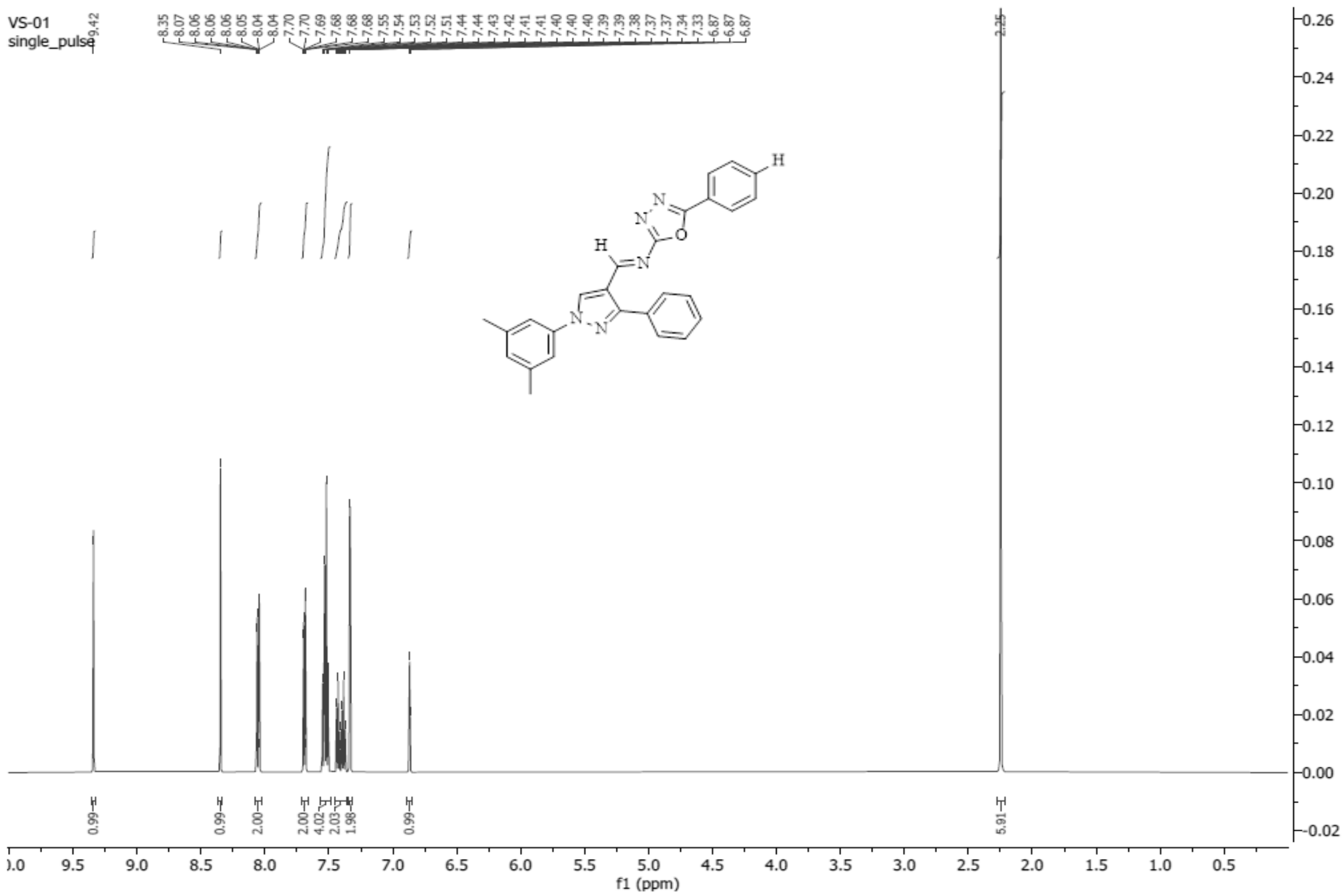

Spectrum 1: Proton spectrum of **VS-01**

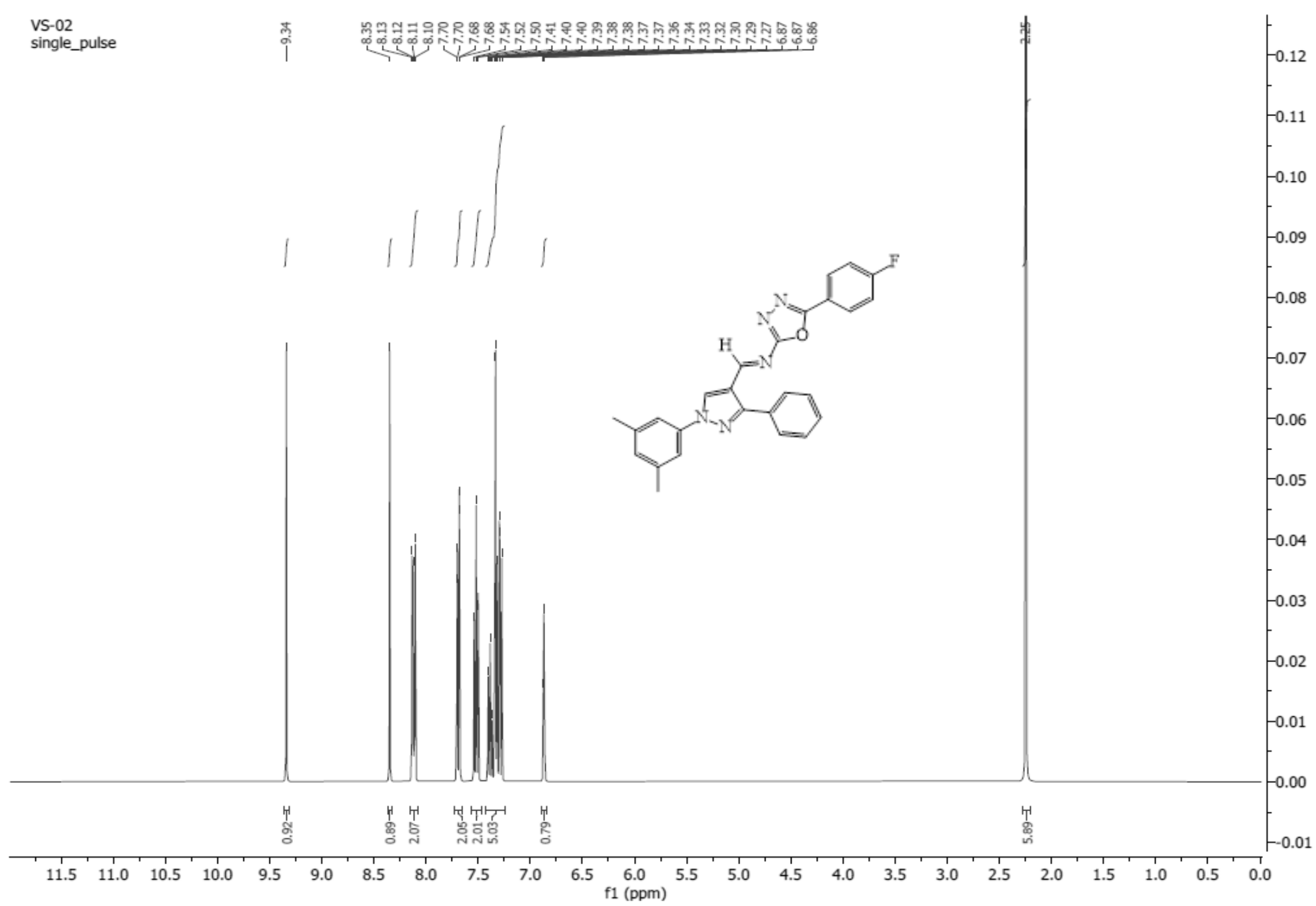

Spectrum 2: Proton spectrum of **VS-02**

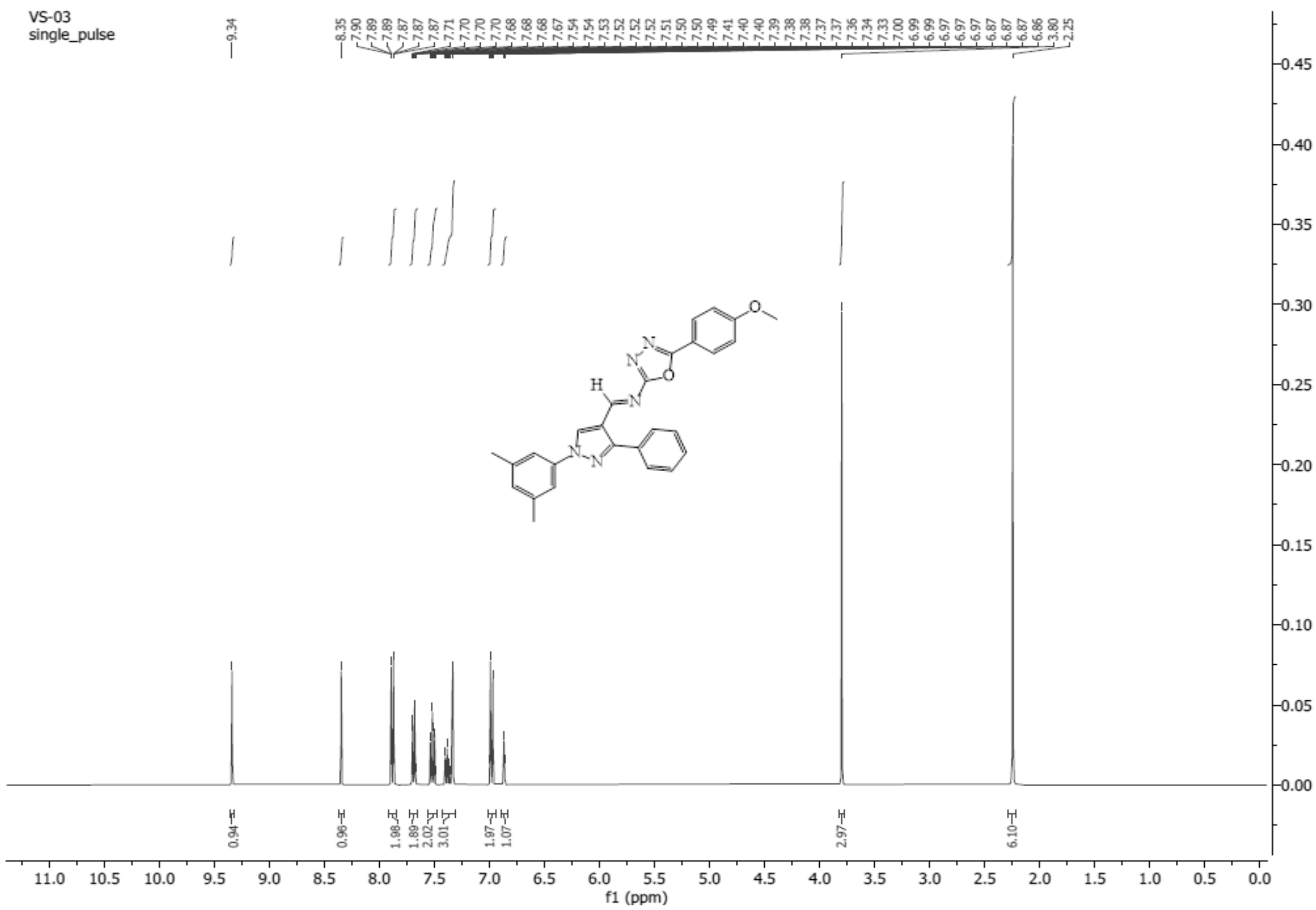

Spectrum 3: Proton spectrum of **VS-03**

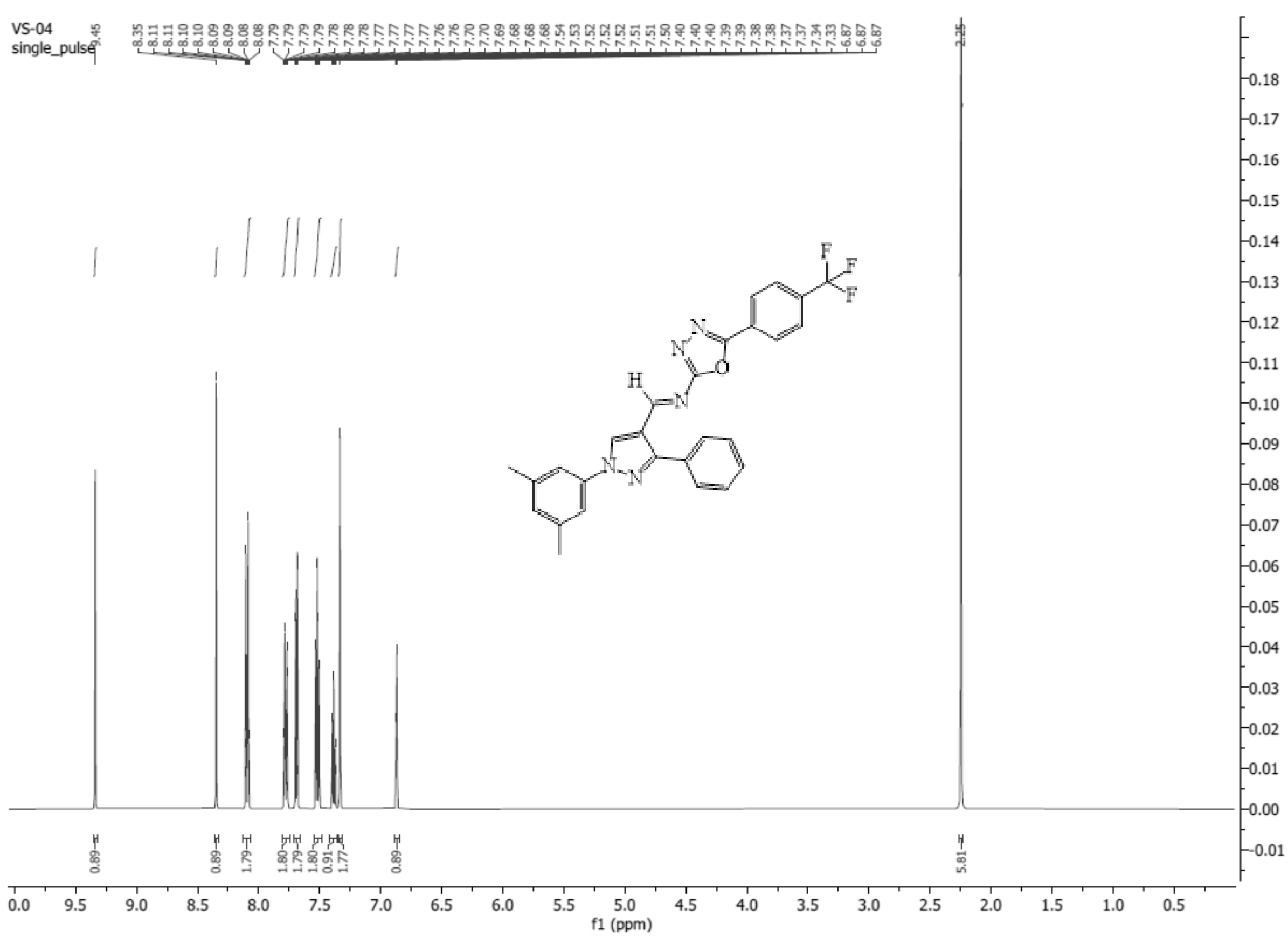

Spectrum 4: Proton spectrum of **VS-04**

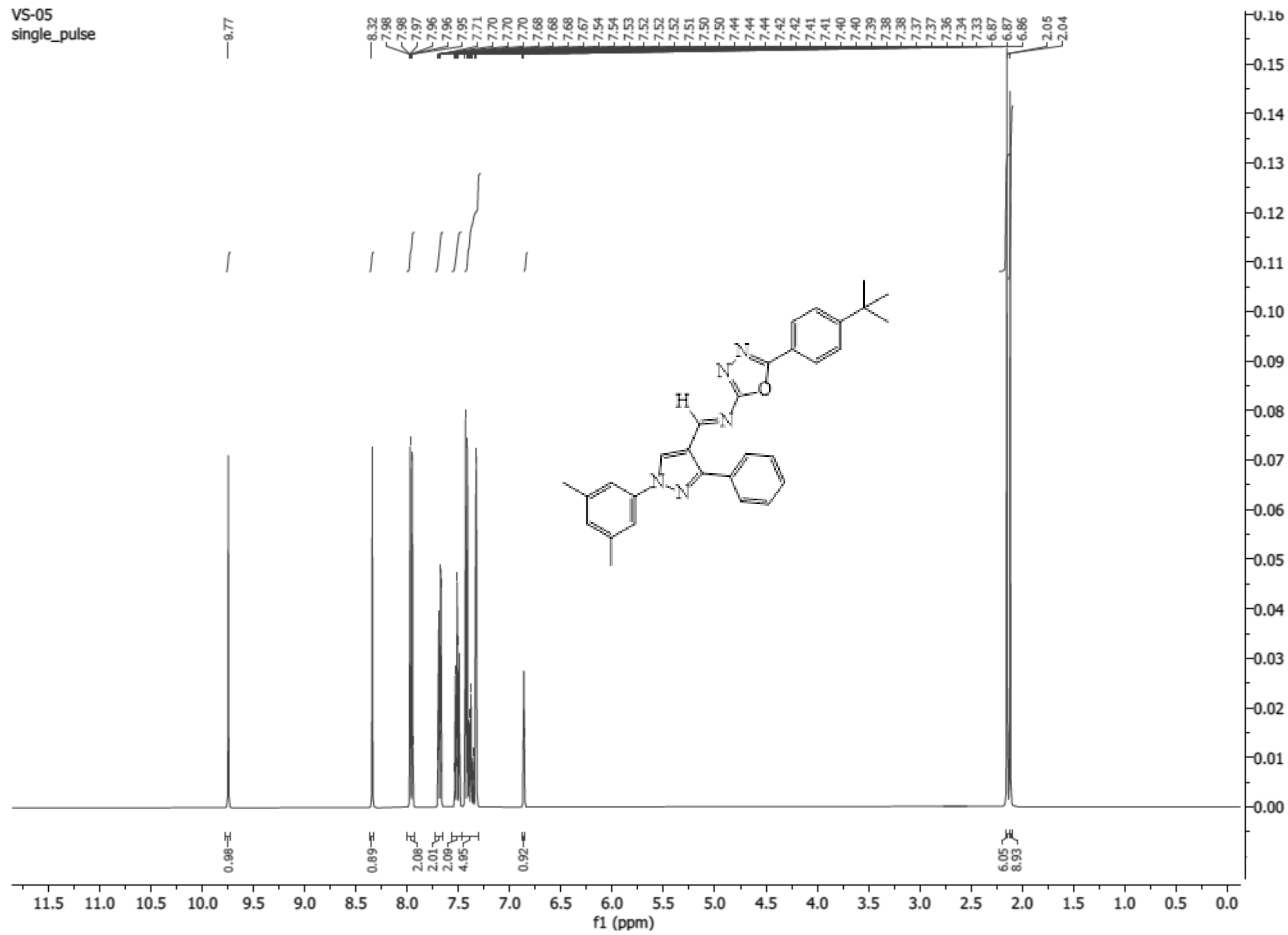

Spectrum 5: Proton spectrum of **VS-05**

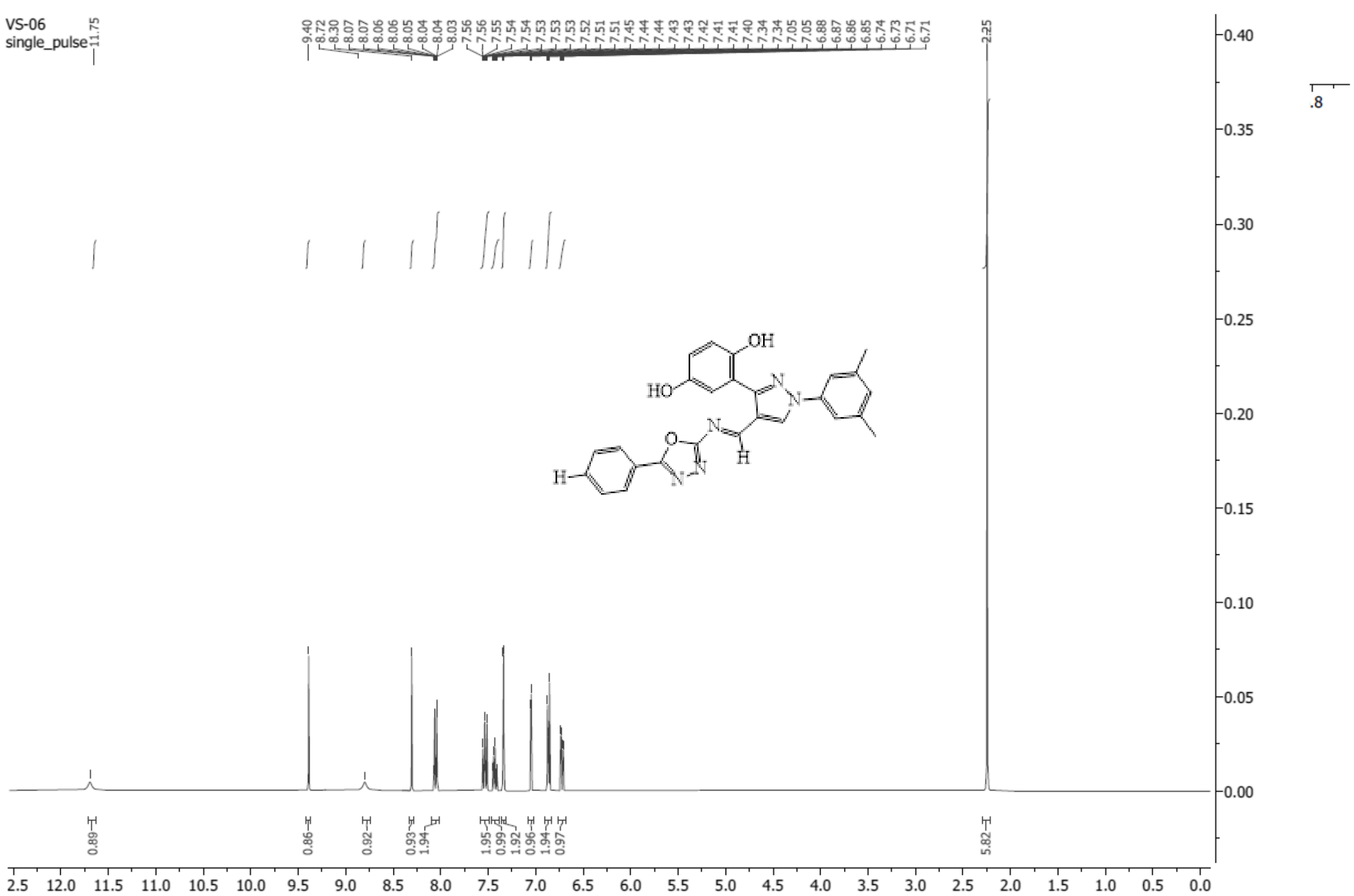

Spectrum 6: Proton spectrum of **VS-06**

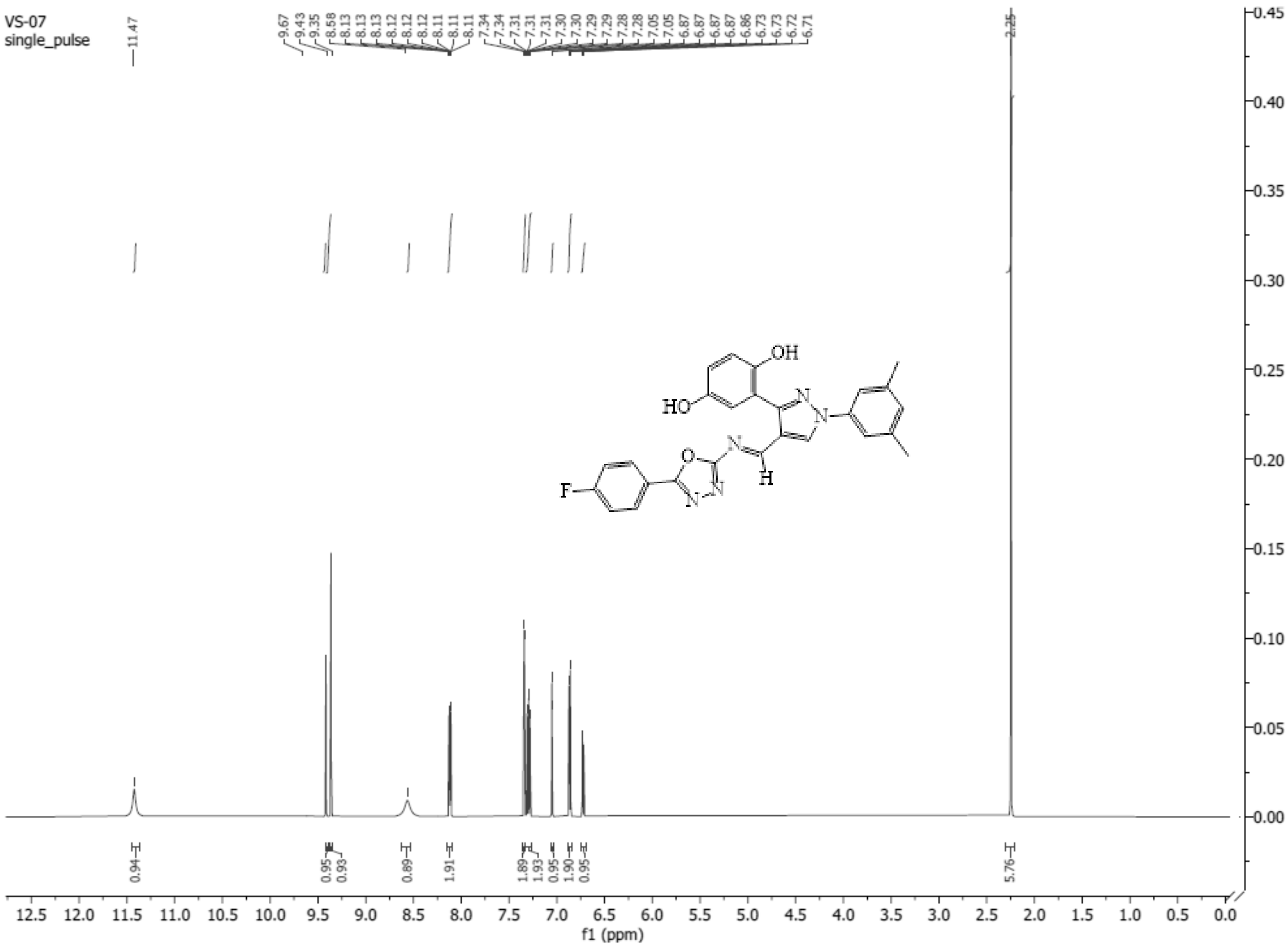

Spectrum 7: Proton spectrum of **VS-07**

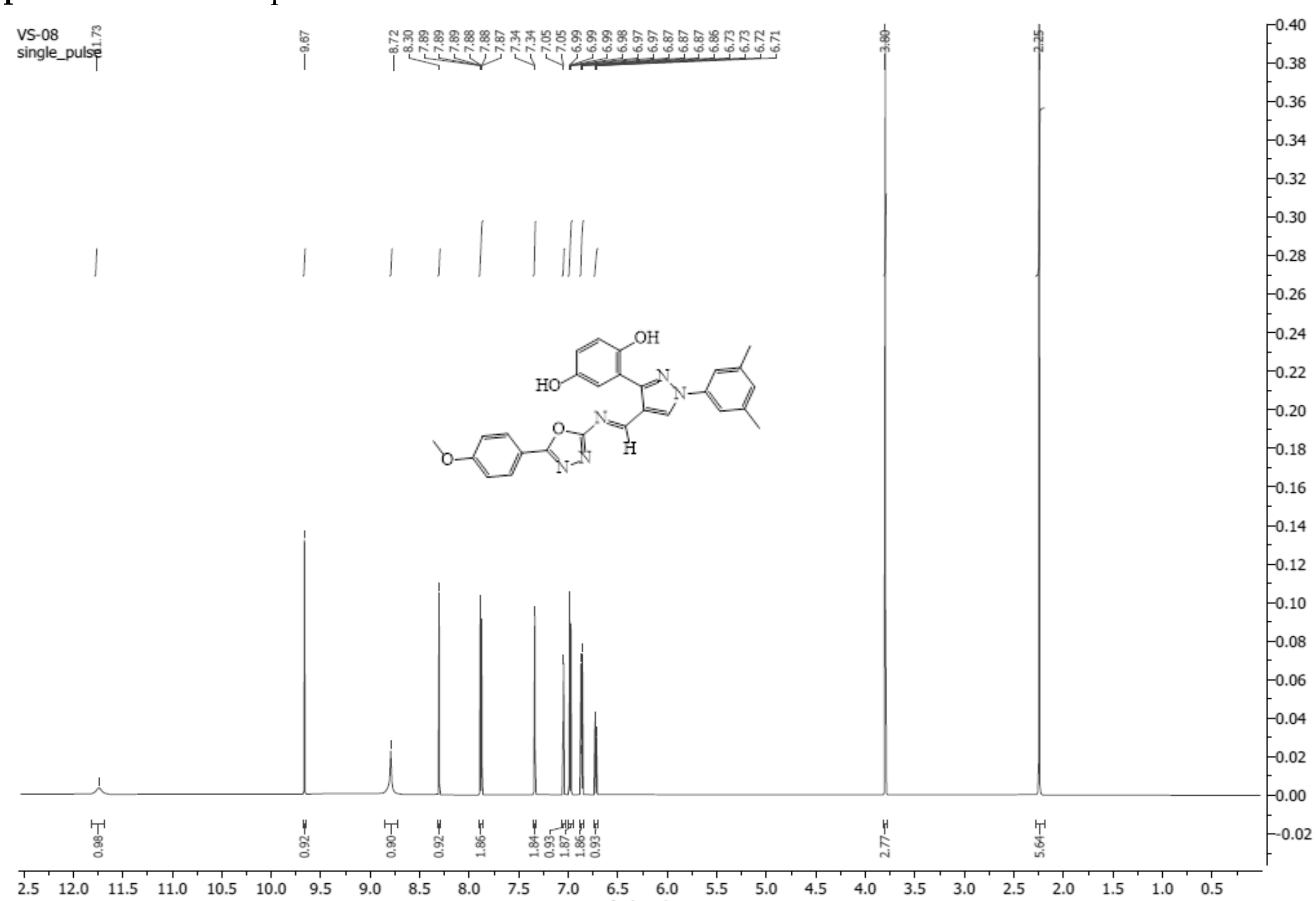

Spectrum 8: Proton spectrum of **VS-08**

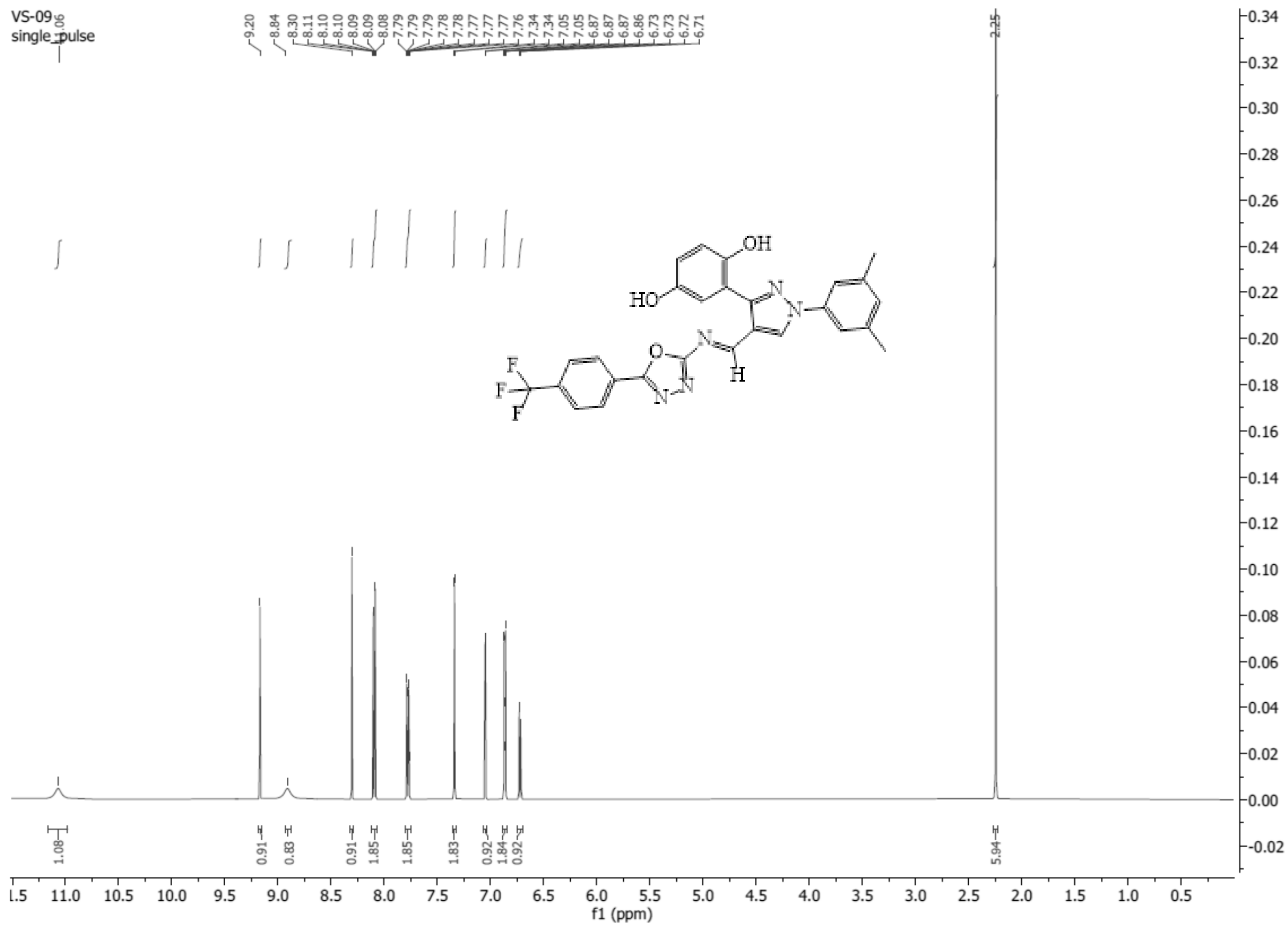

Spectrum 9: Proton spectrum of **VS-09**

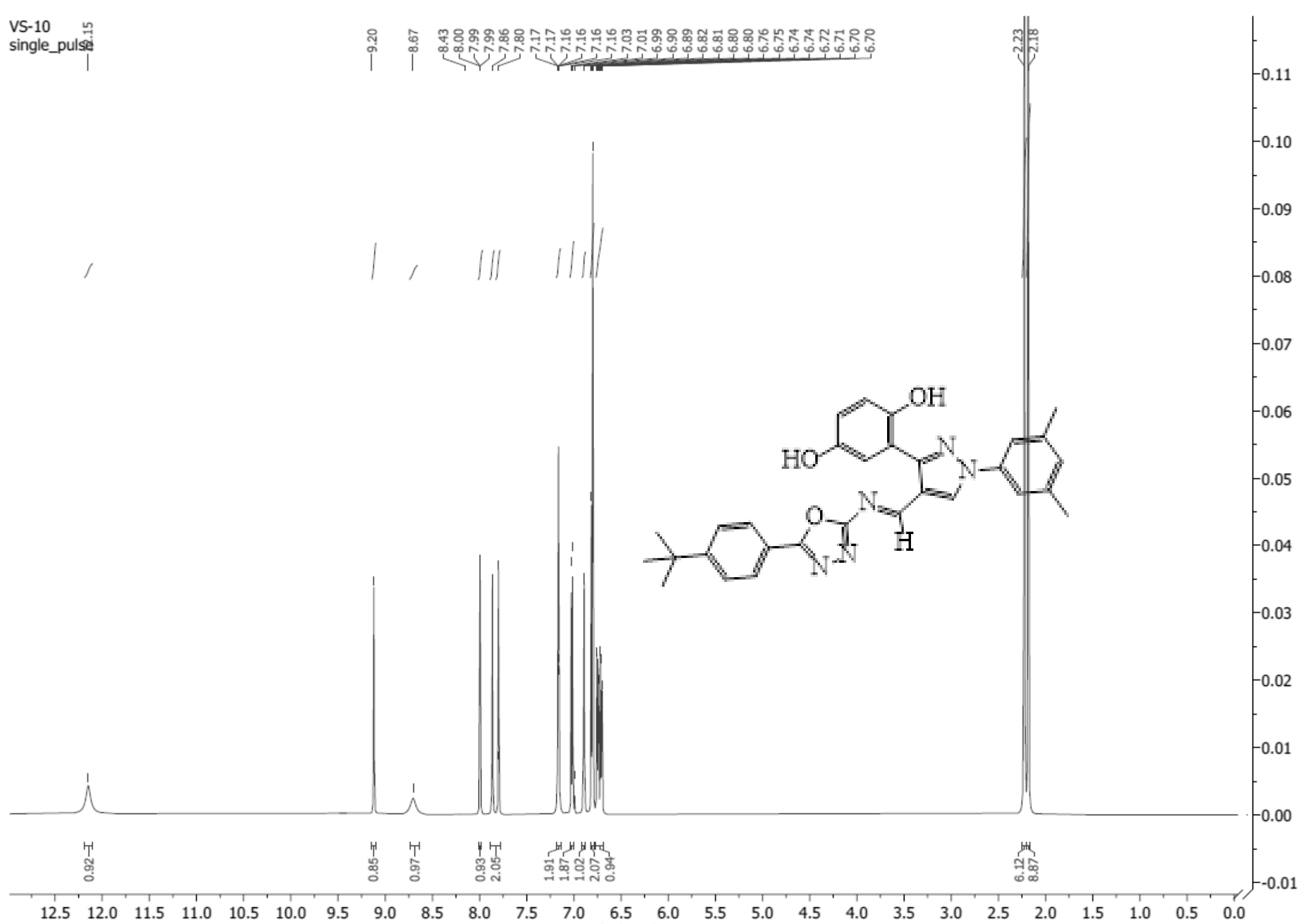

Spectrum 10: Proton spectrum of **VS-10**

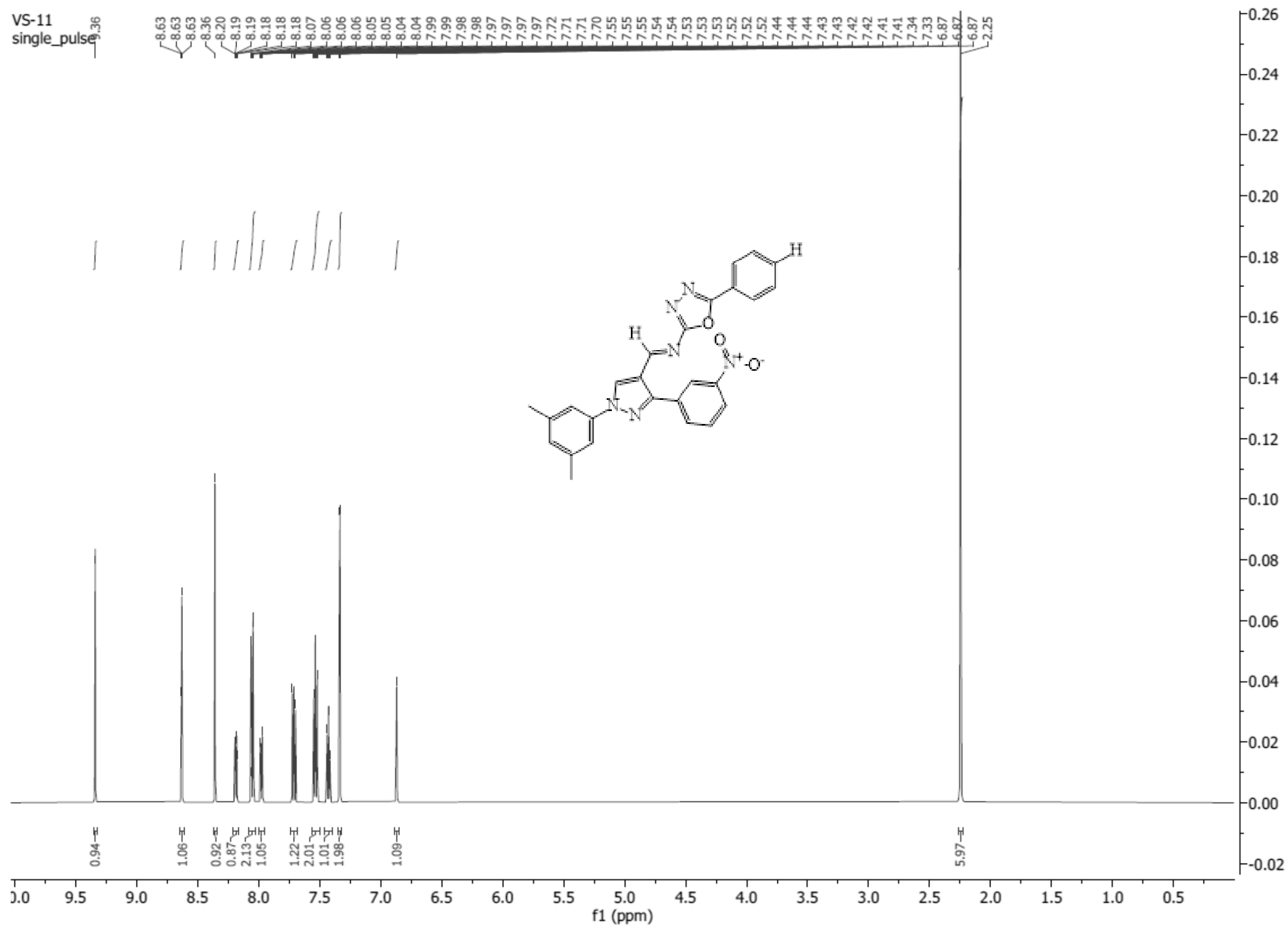

Spectrum 11: Proton spectrum of **VS-11**

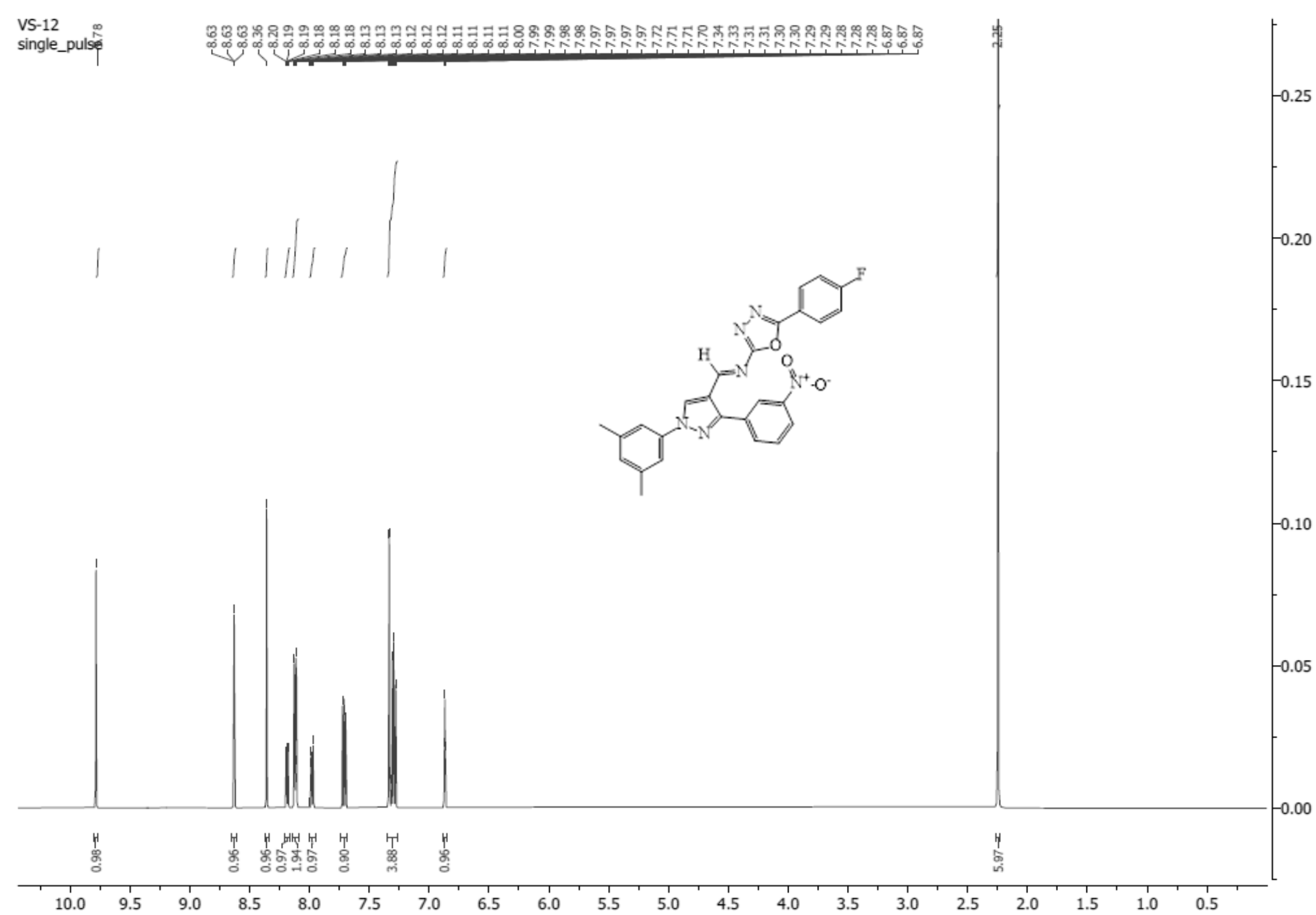

Spectrum 12: Proton spectrum of **VS-12**

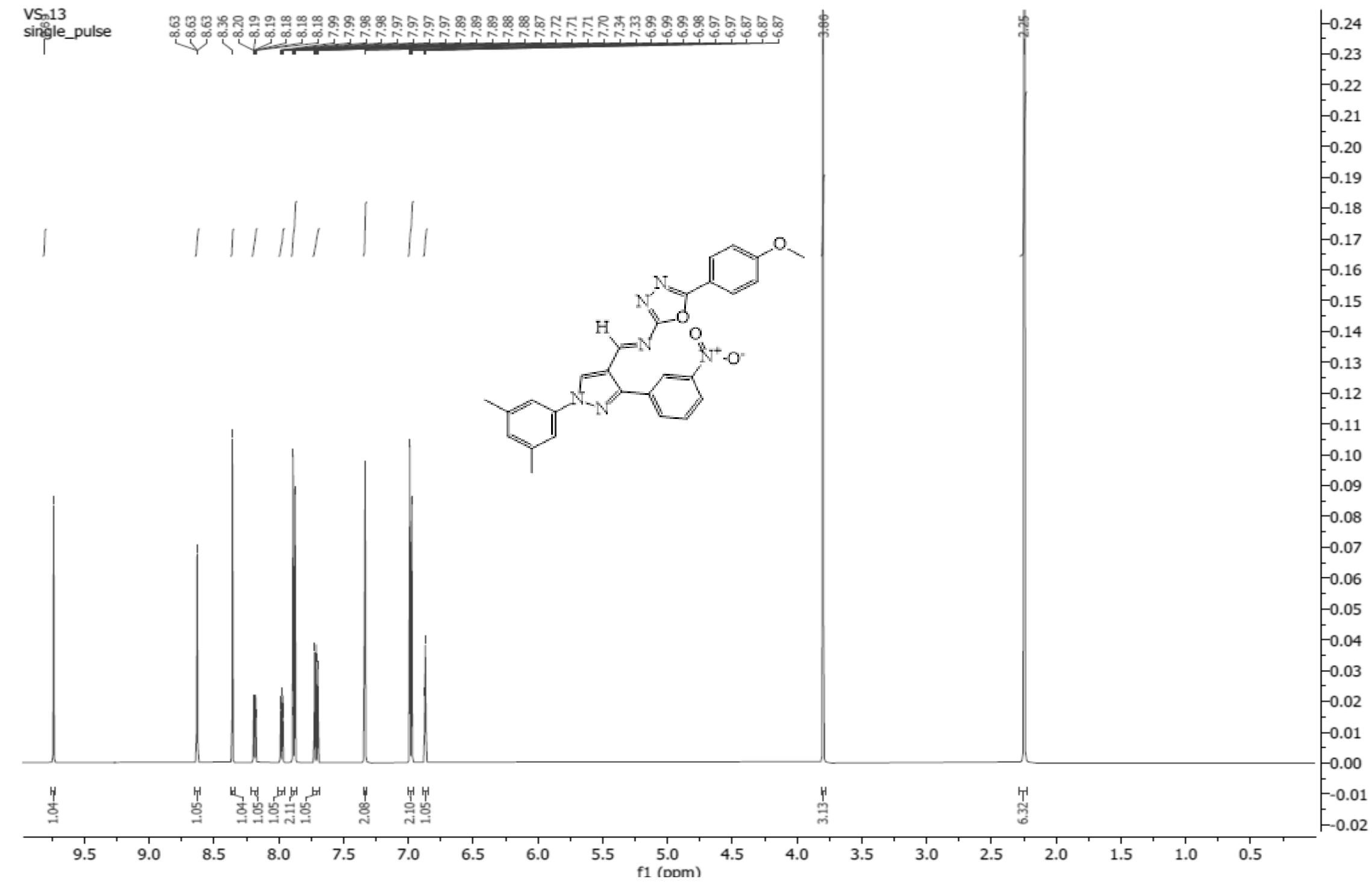

Spectrum 13: Proton spectrum of **VS-13**

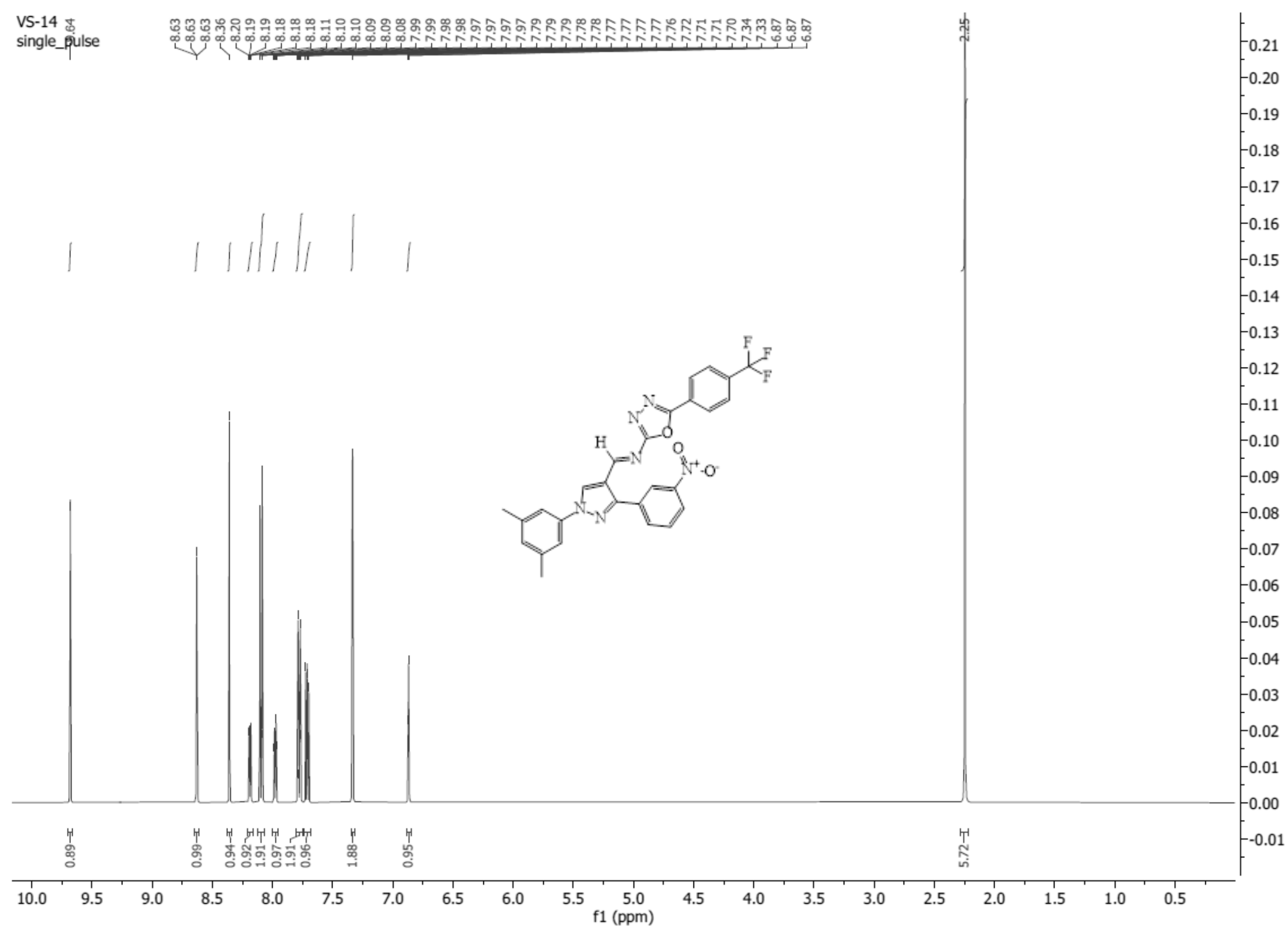

Spectrum 14: Proton spectrum of **VS-14**

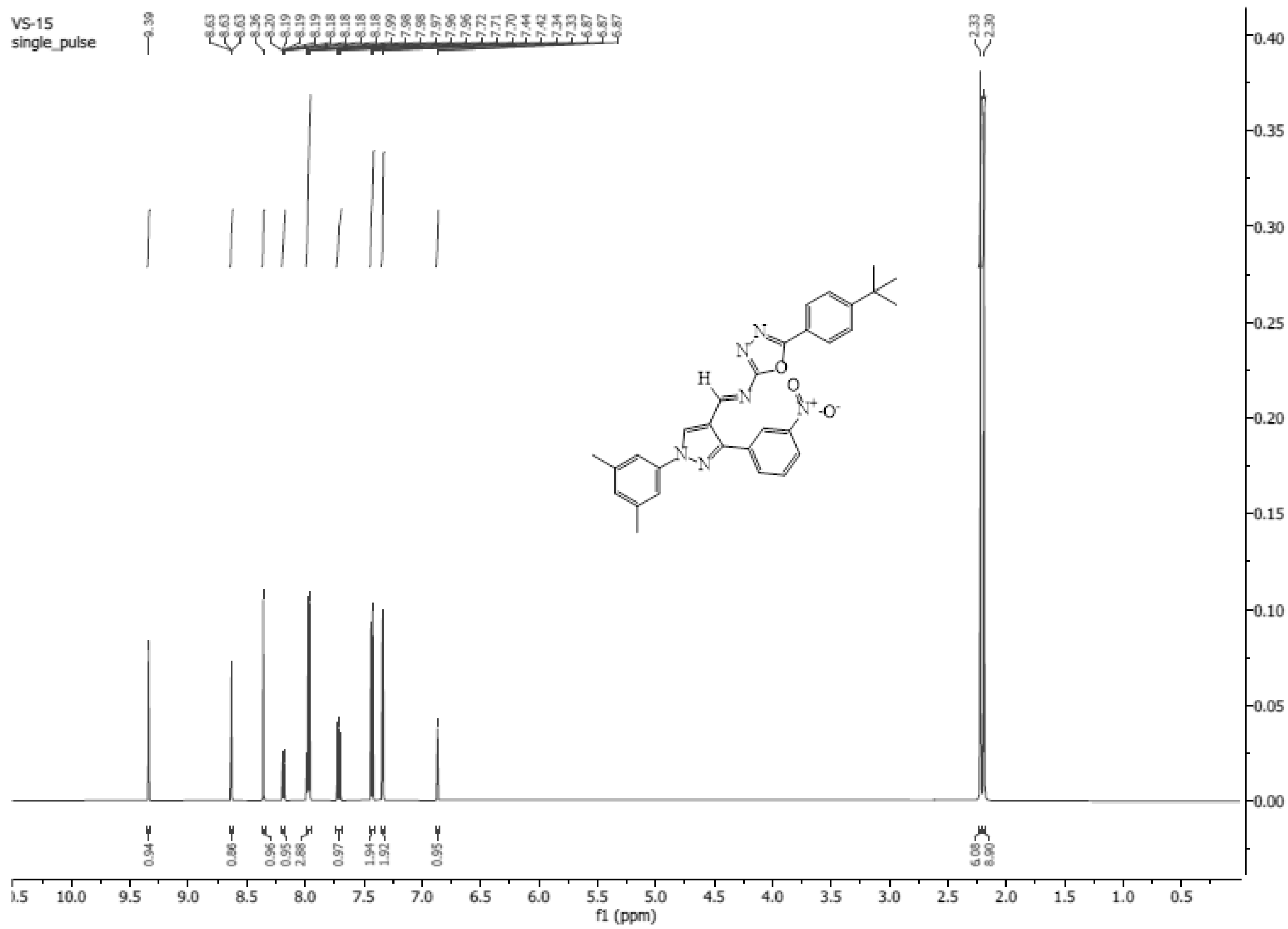

Spectrum 15: Proton spectrum of **VS-15**

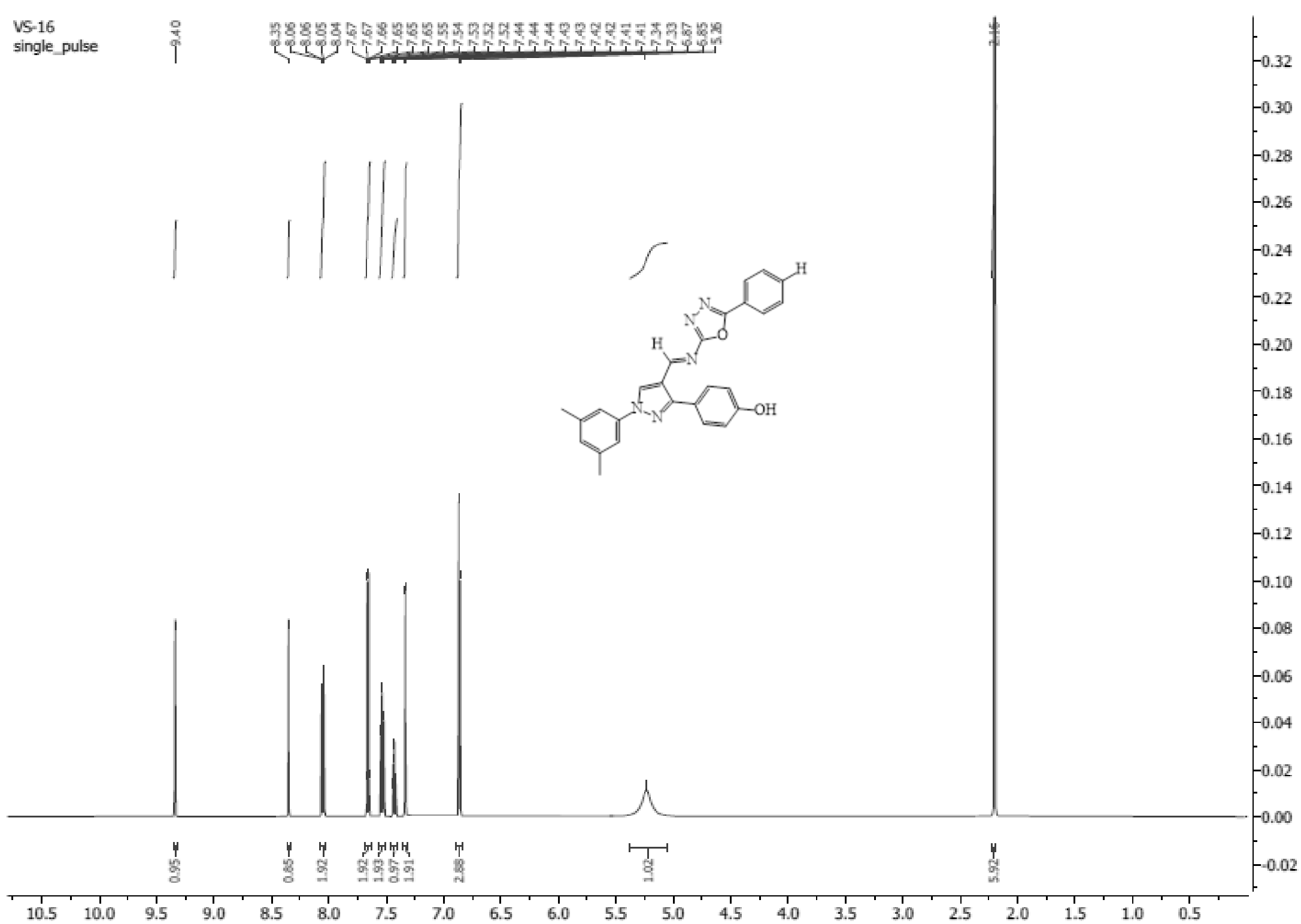

Spectrum 16: Proton spectrum of **VS-16**

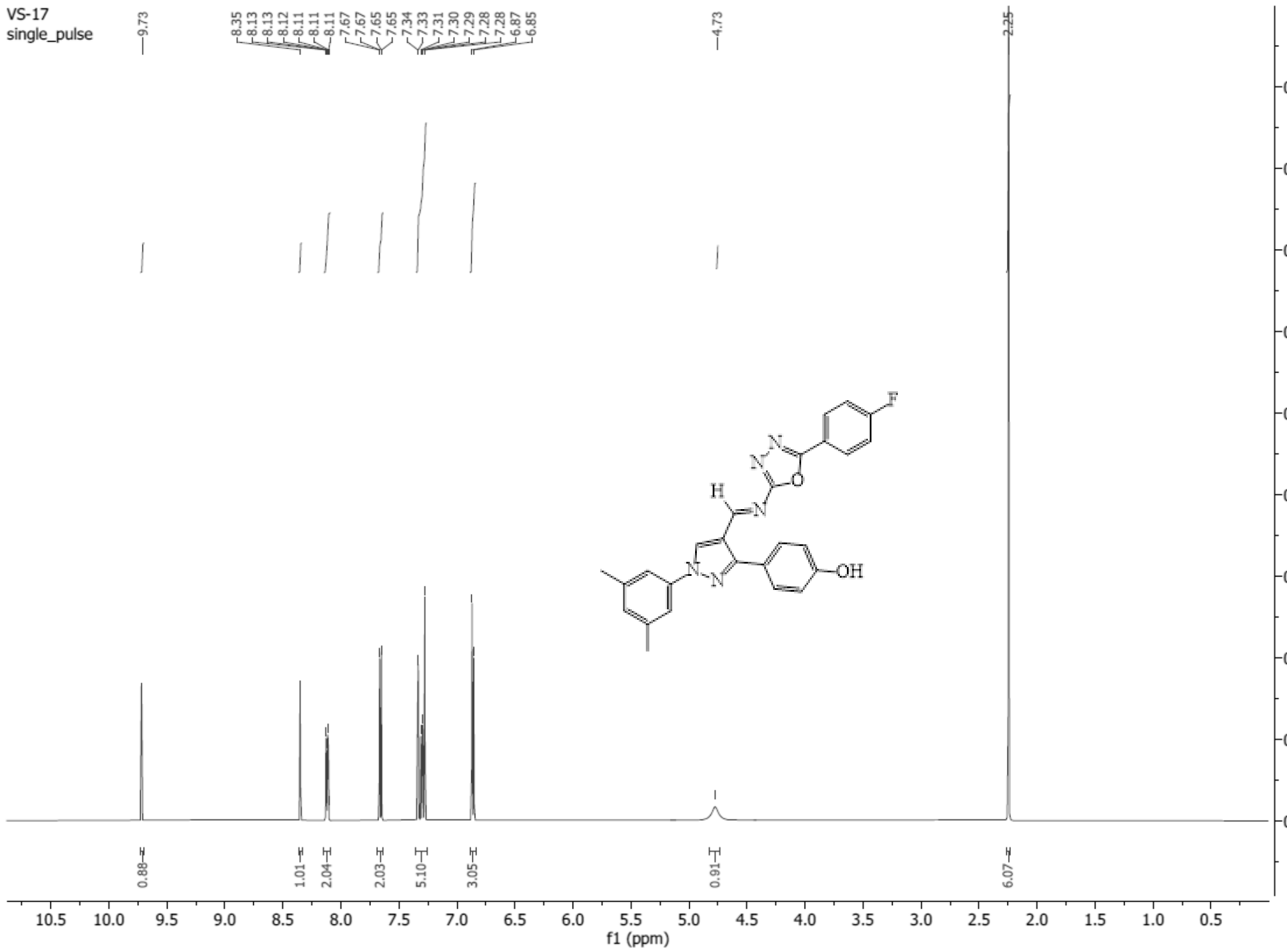

Spectrum 17: Proton spectrum of **VS-17**

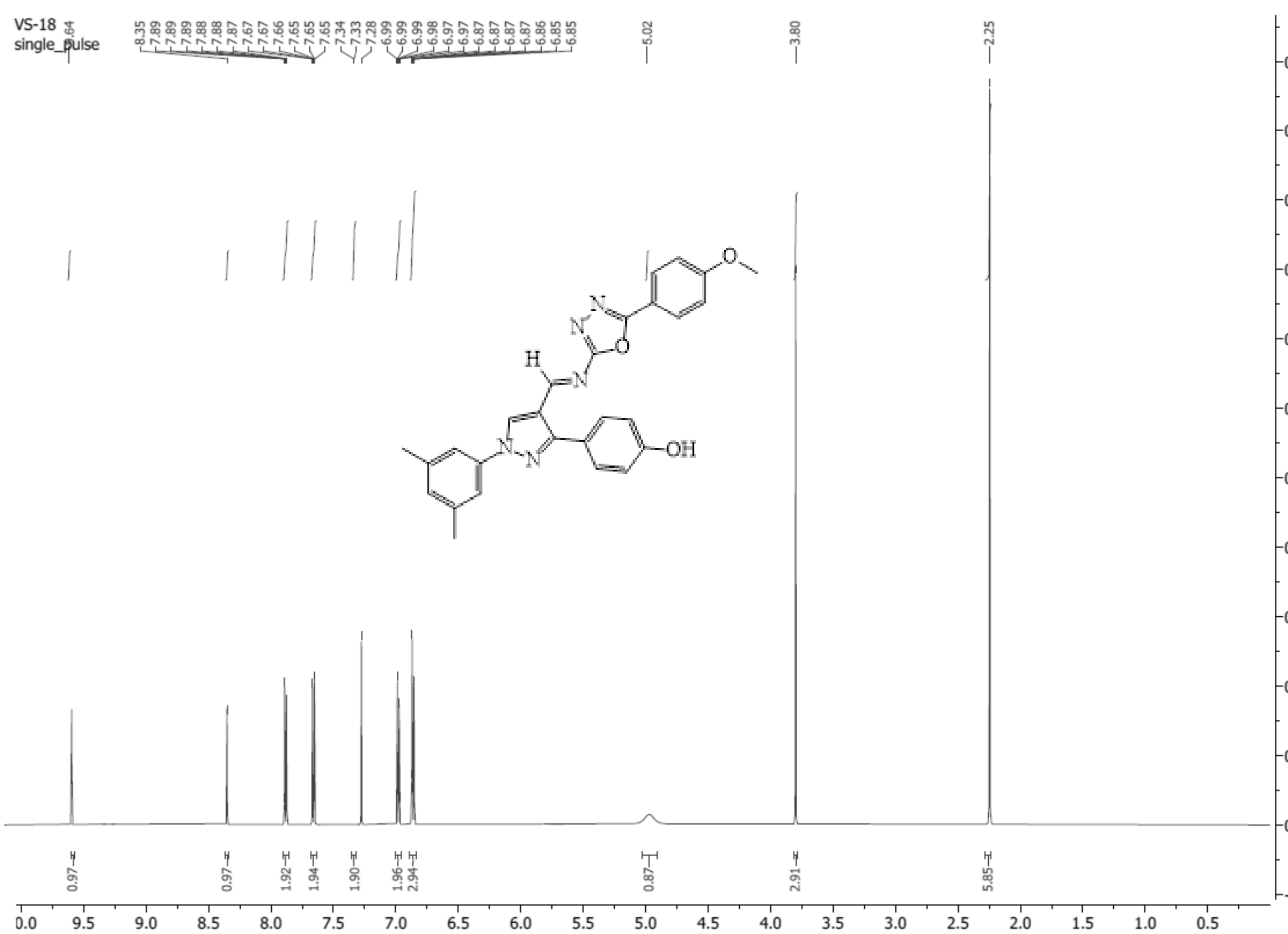

Spectrum 18: Proton spectrum of **VS-18**

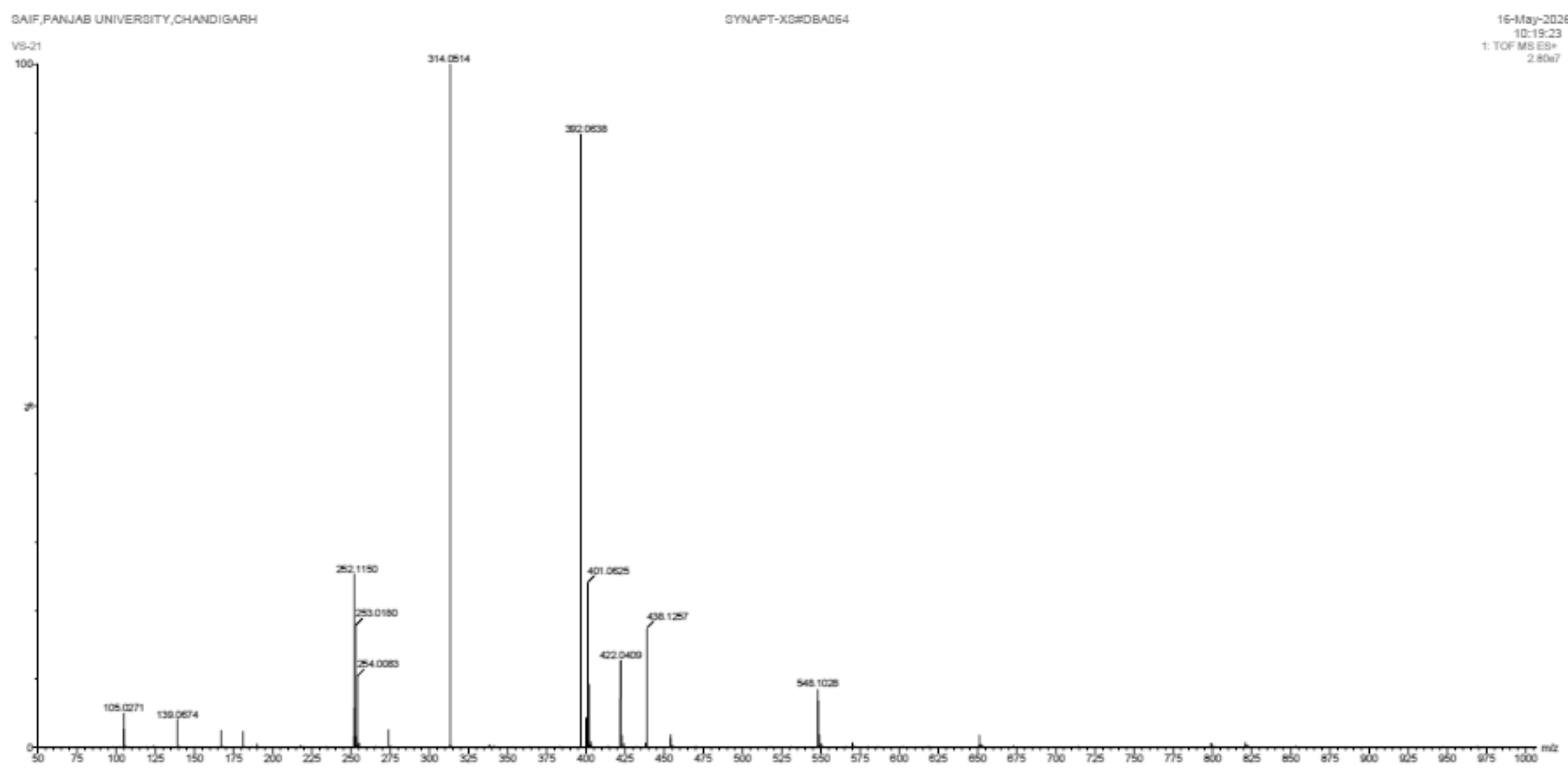

Spectrum 19: Mass Spectra of **VS-01**

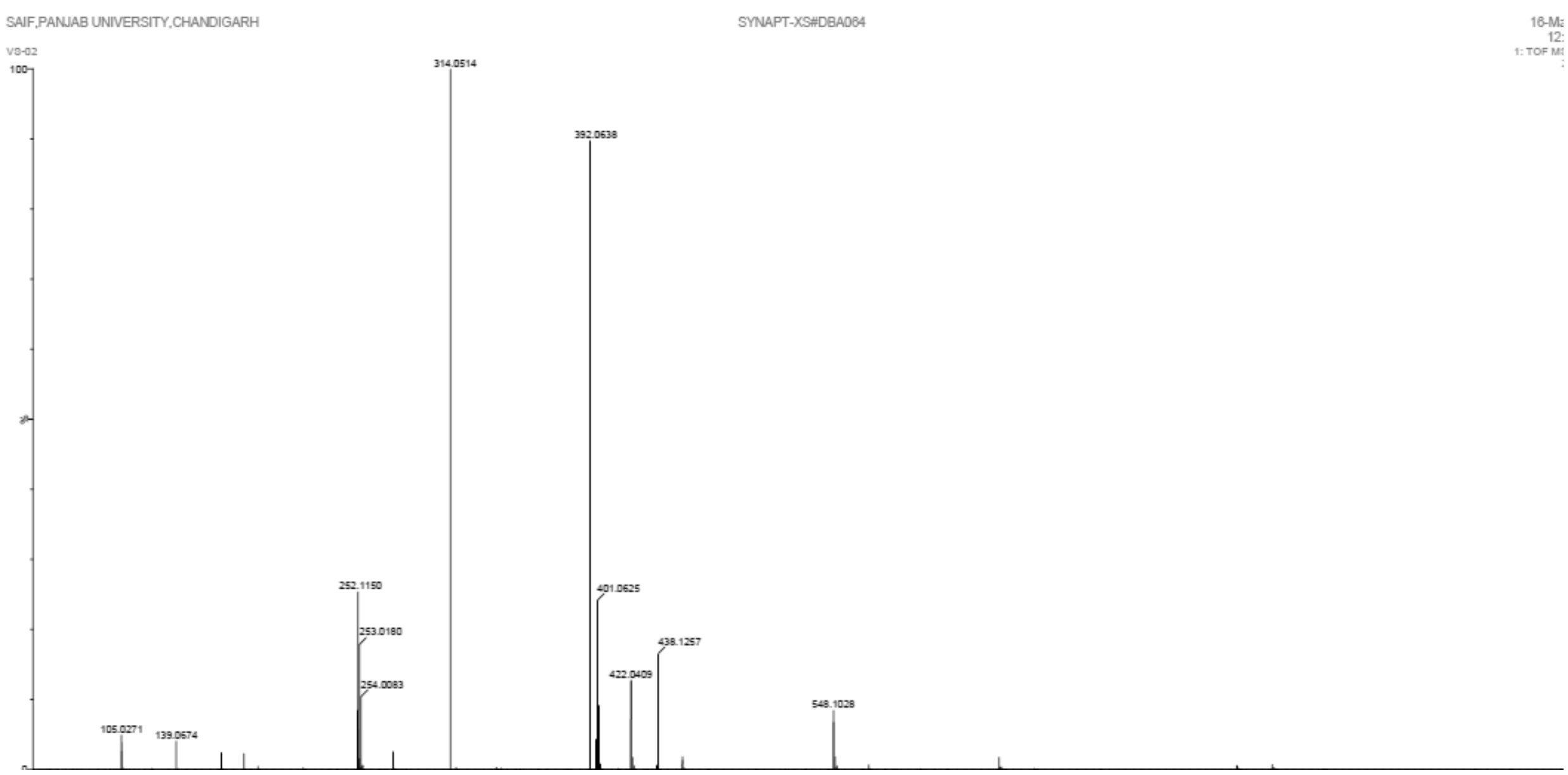

Spectrum 20: Mass Spectra of **VS-02**

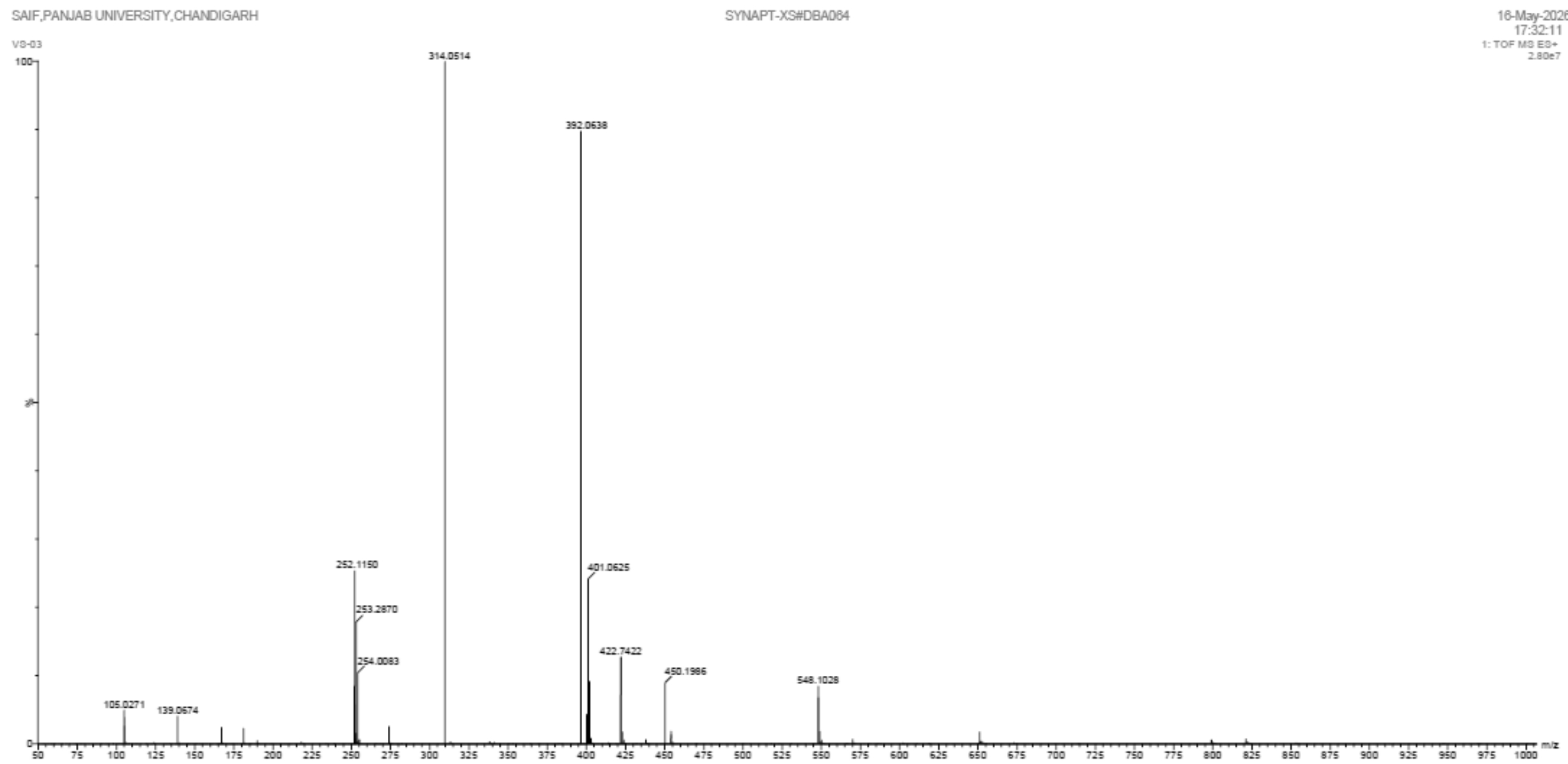

Spectrum 21: Mass Spectra of **VS-03**

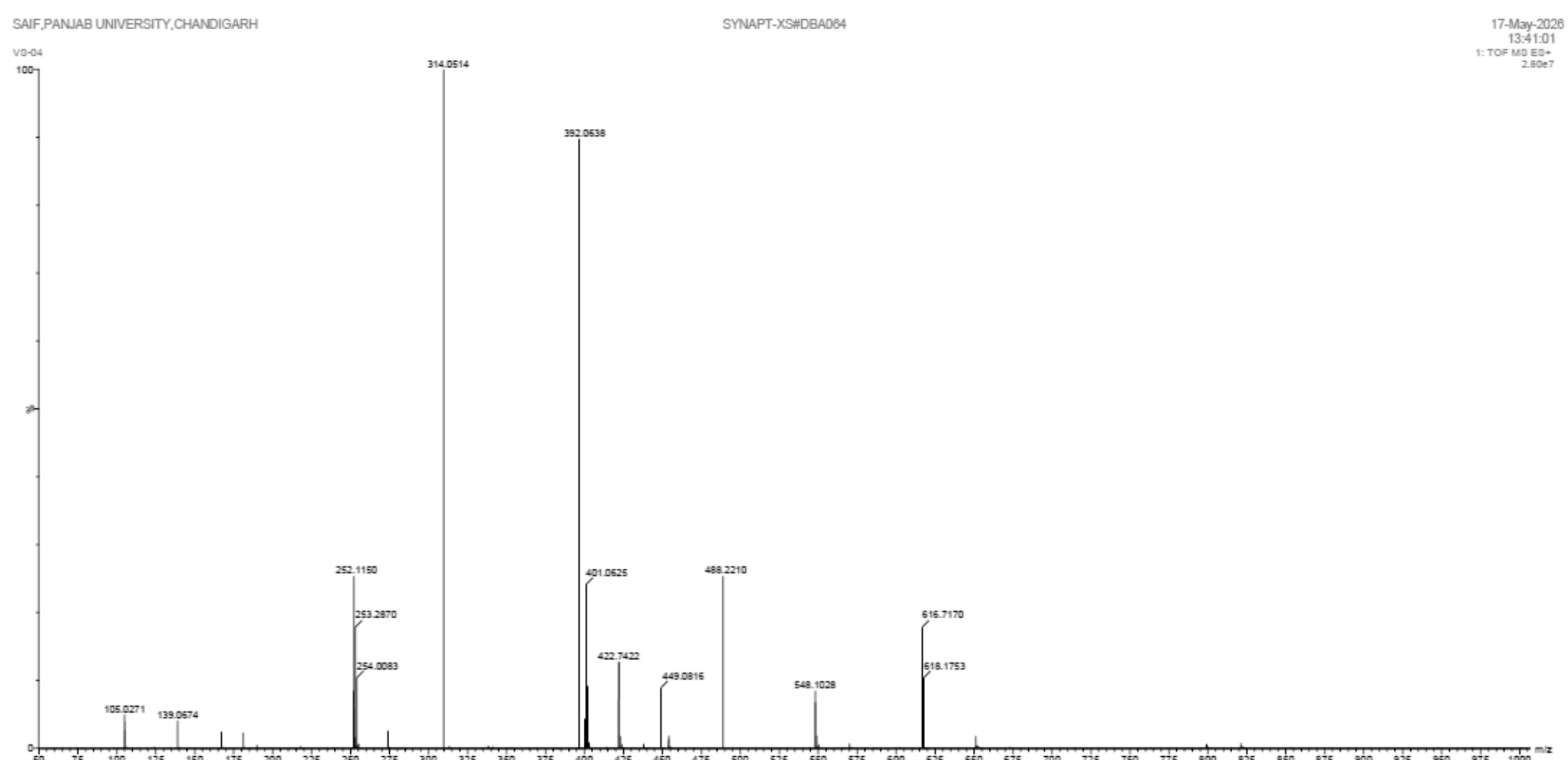

Spectrum 22: Mass Spectra of **VS-04**

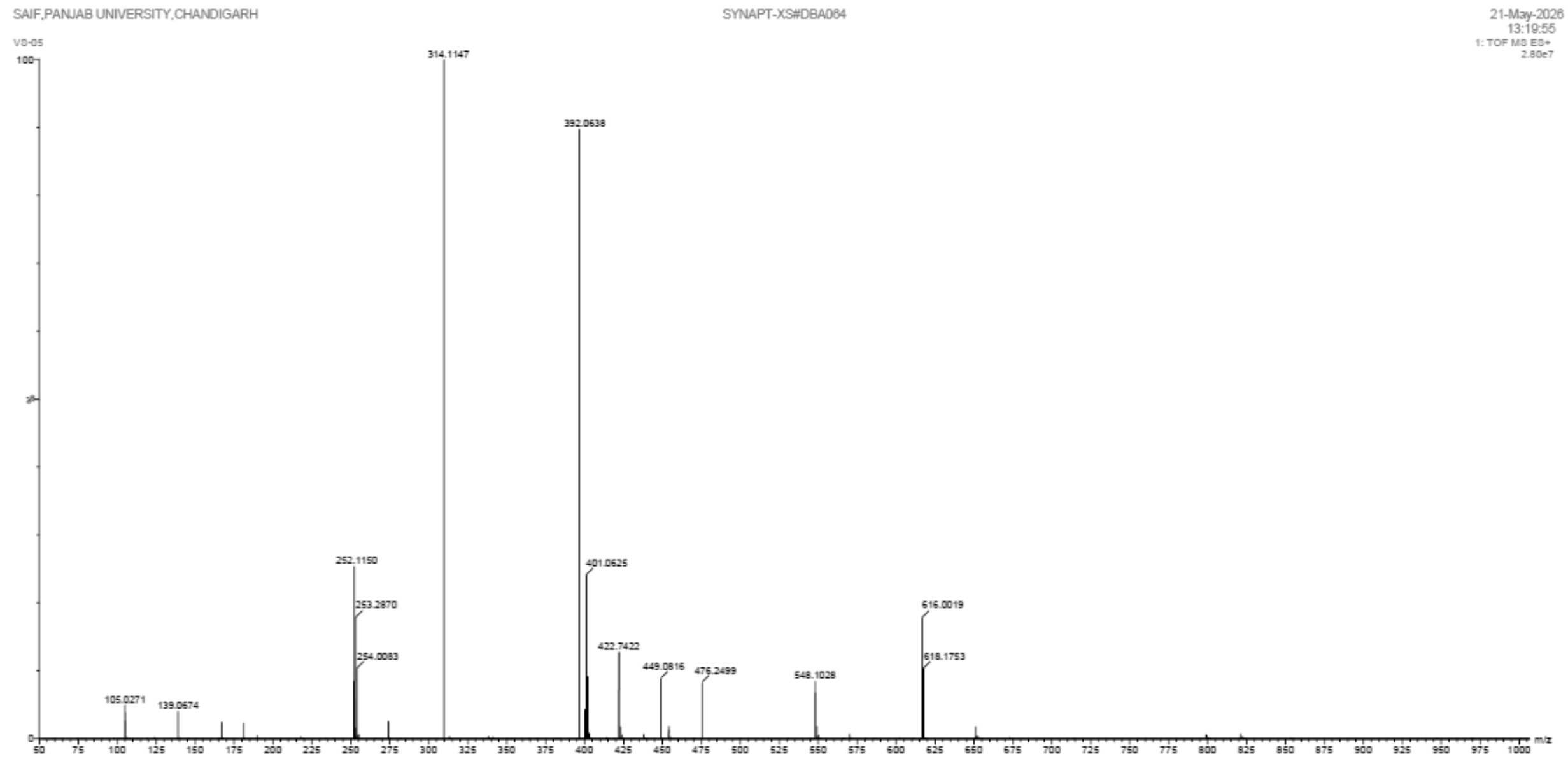

Spectrum 23: Mass Spectra of **VS-05**

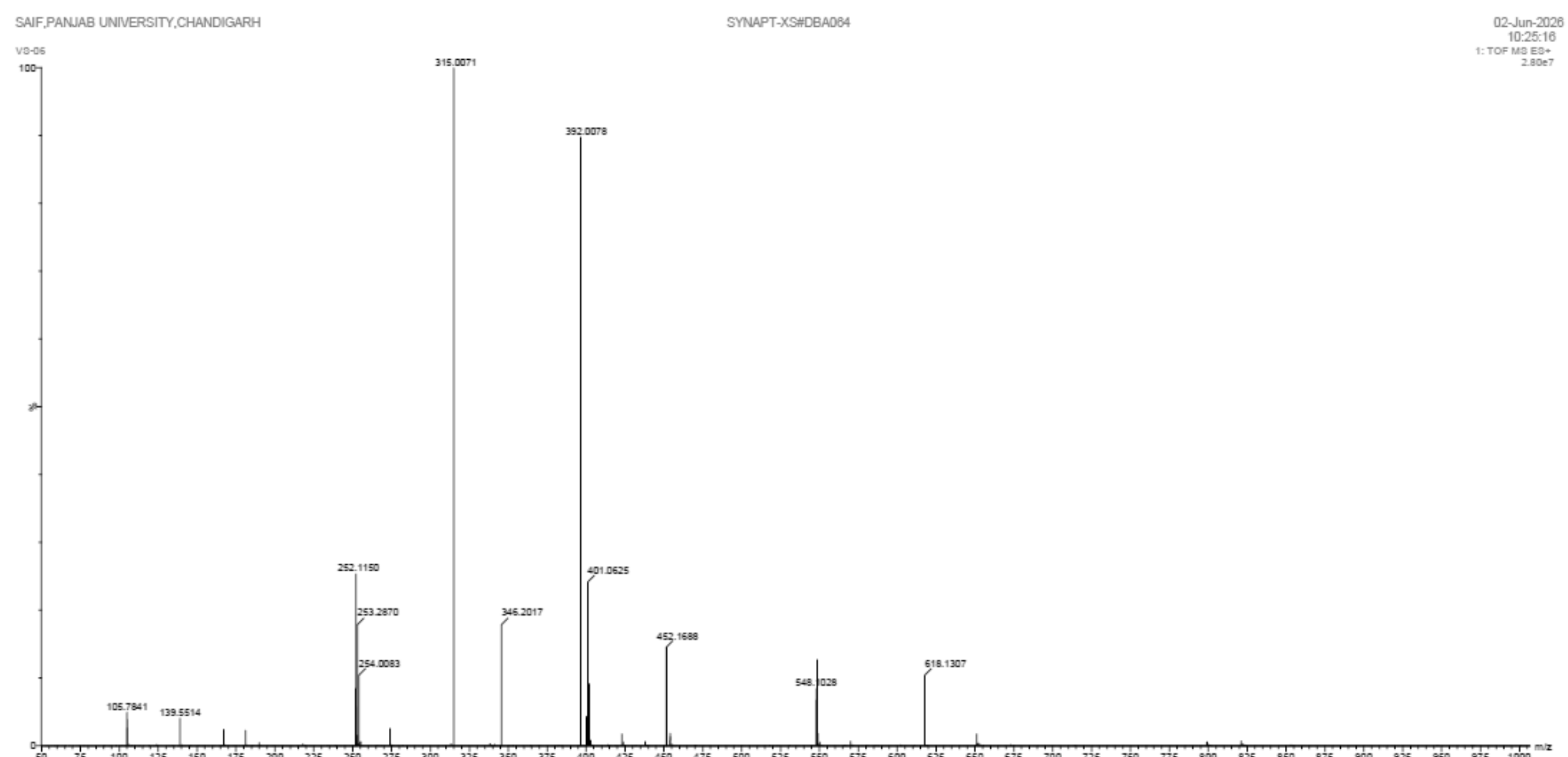

Spectrum 24: Mass Spectra of **VS-06**

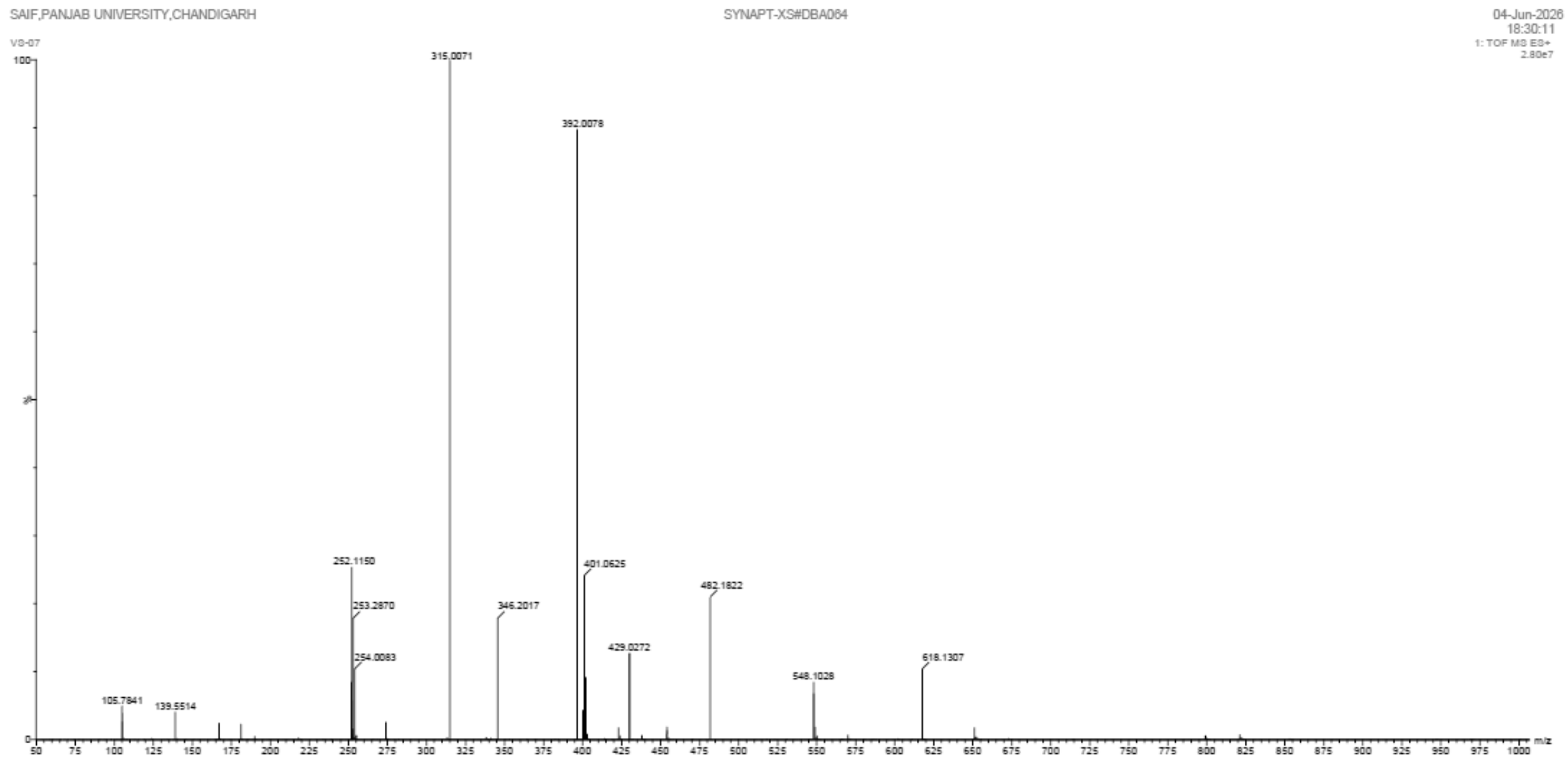

Spectrum 25: Mass Spectra of **VS-07**

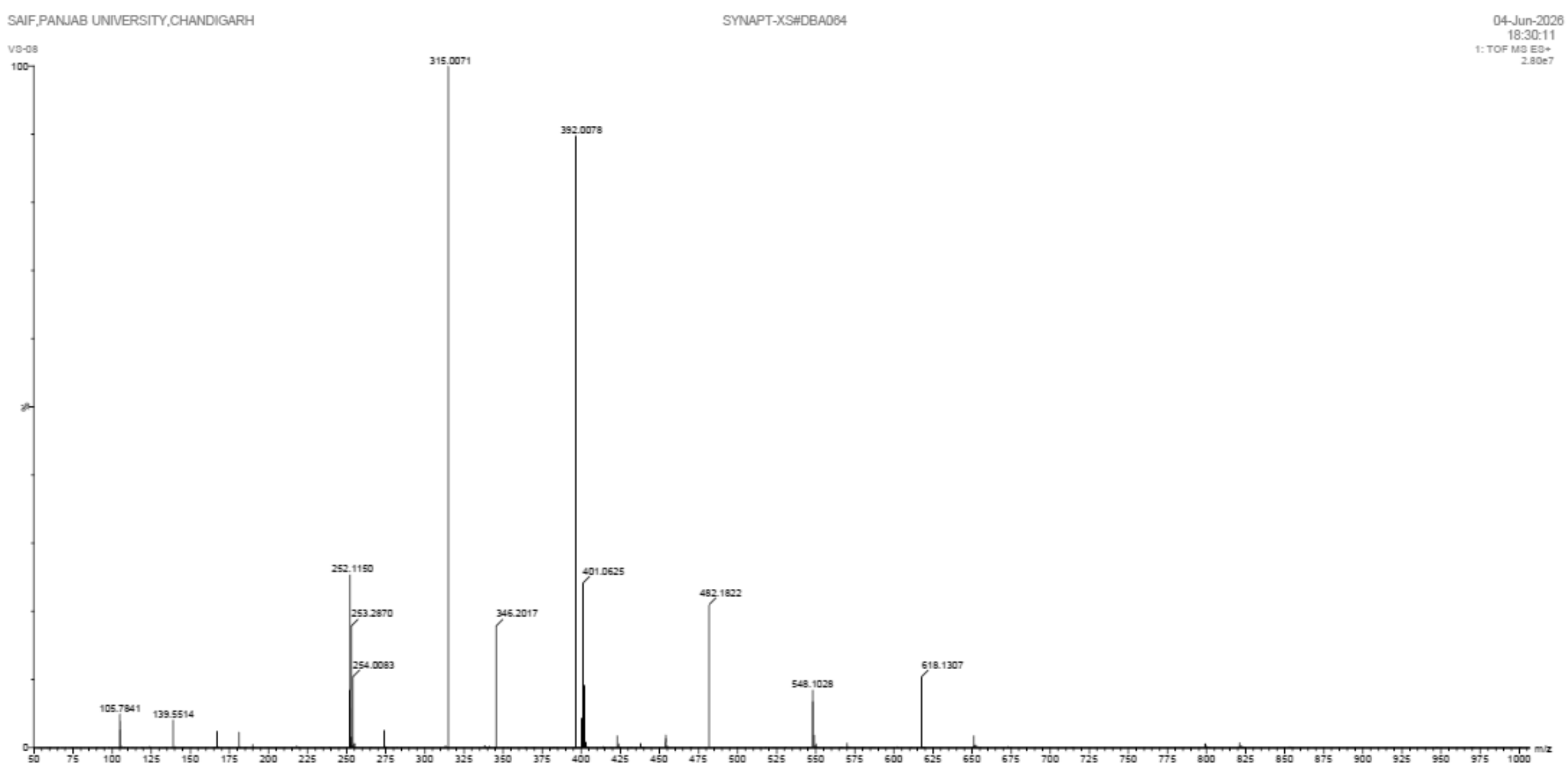

Spectrum 26: Mass Spectra of **VS-08**

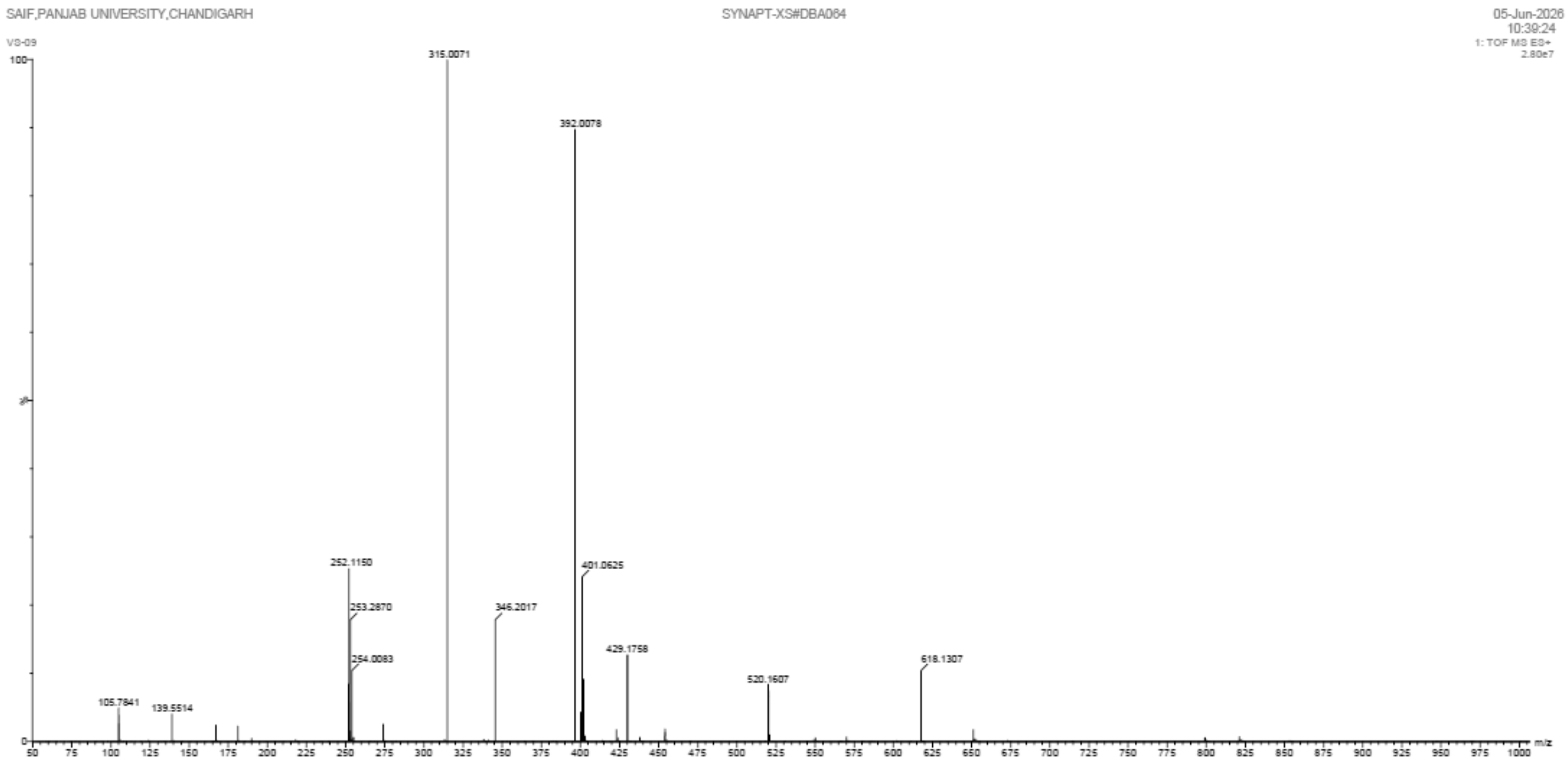

Spectrum 27: Mass Spectra of **VS-09**

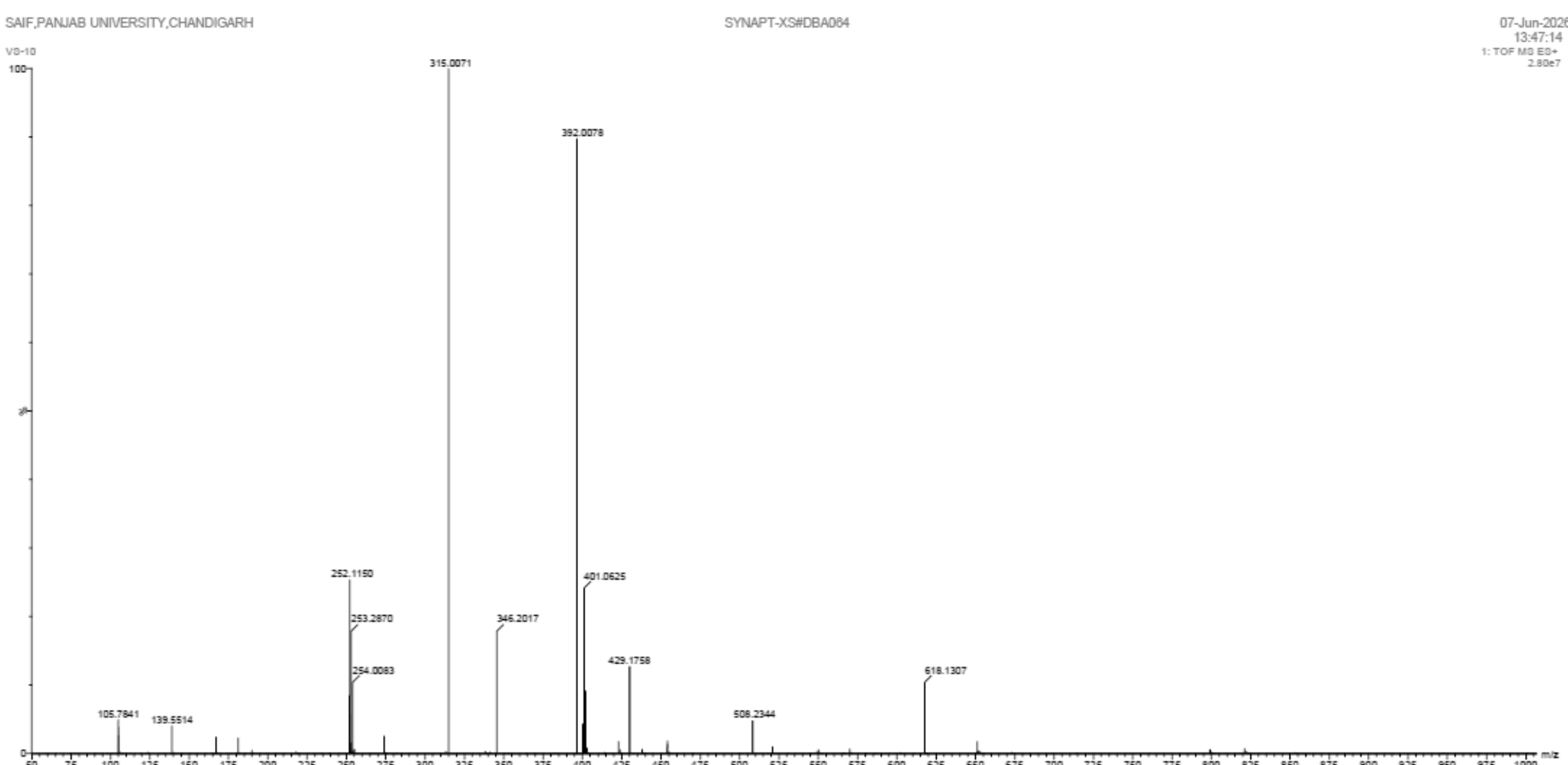

Spectrum 28: Mass Spectra of **VS-10**

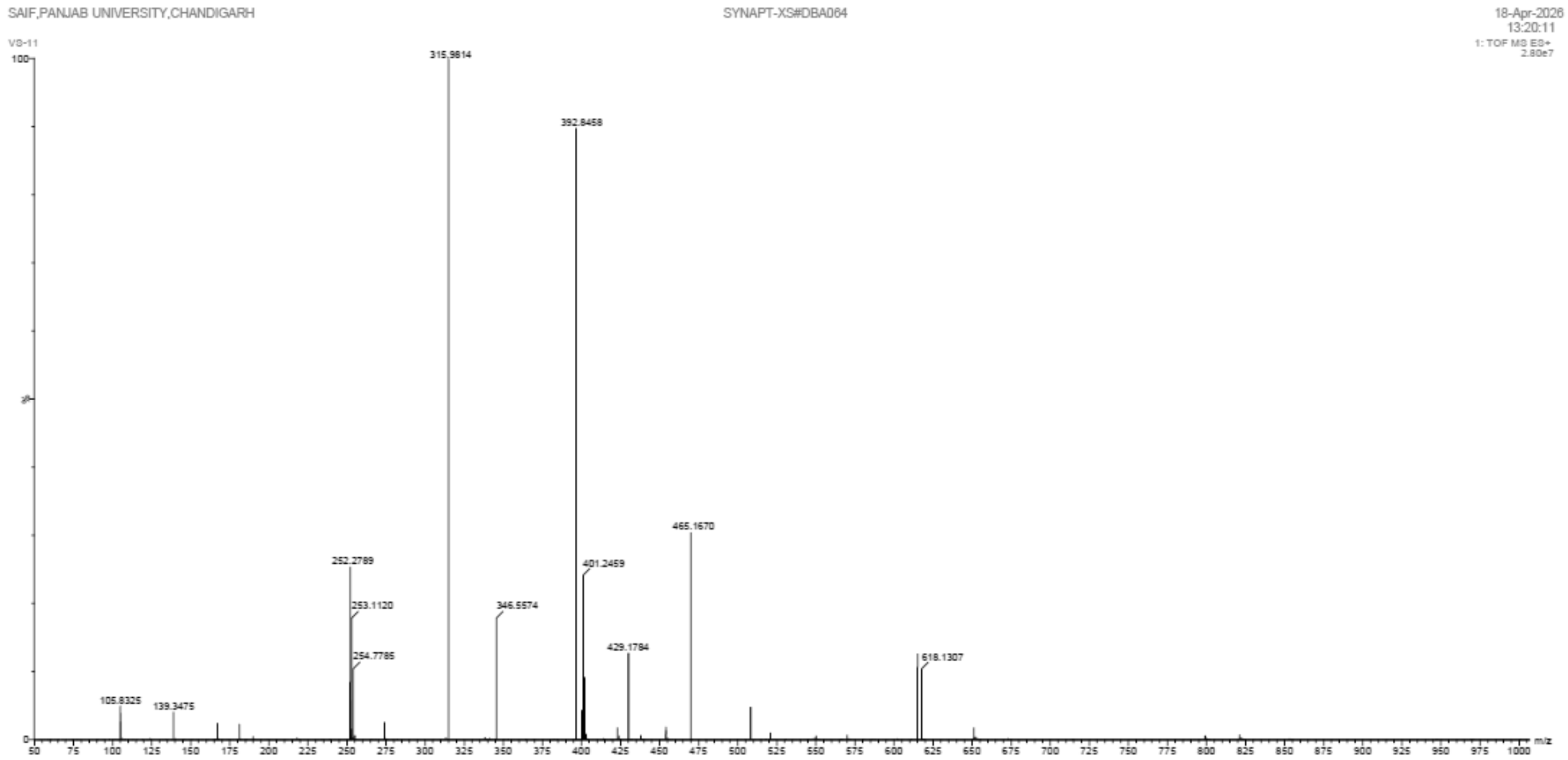

Spectrum 29: Mass Spectra of **VS-11**

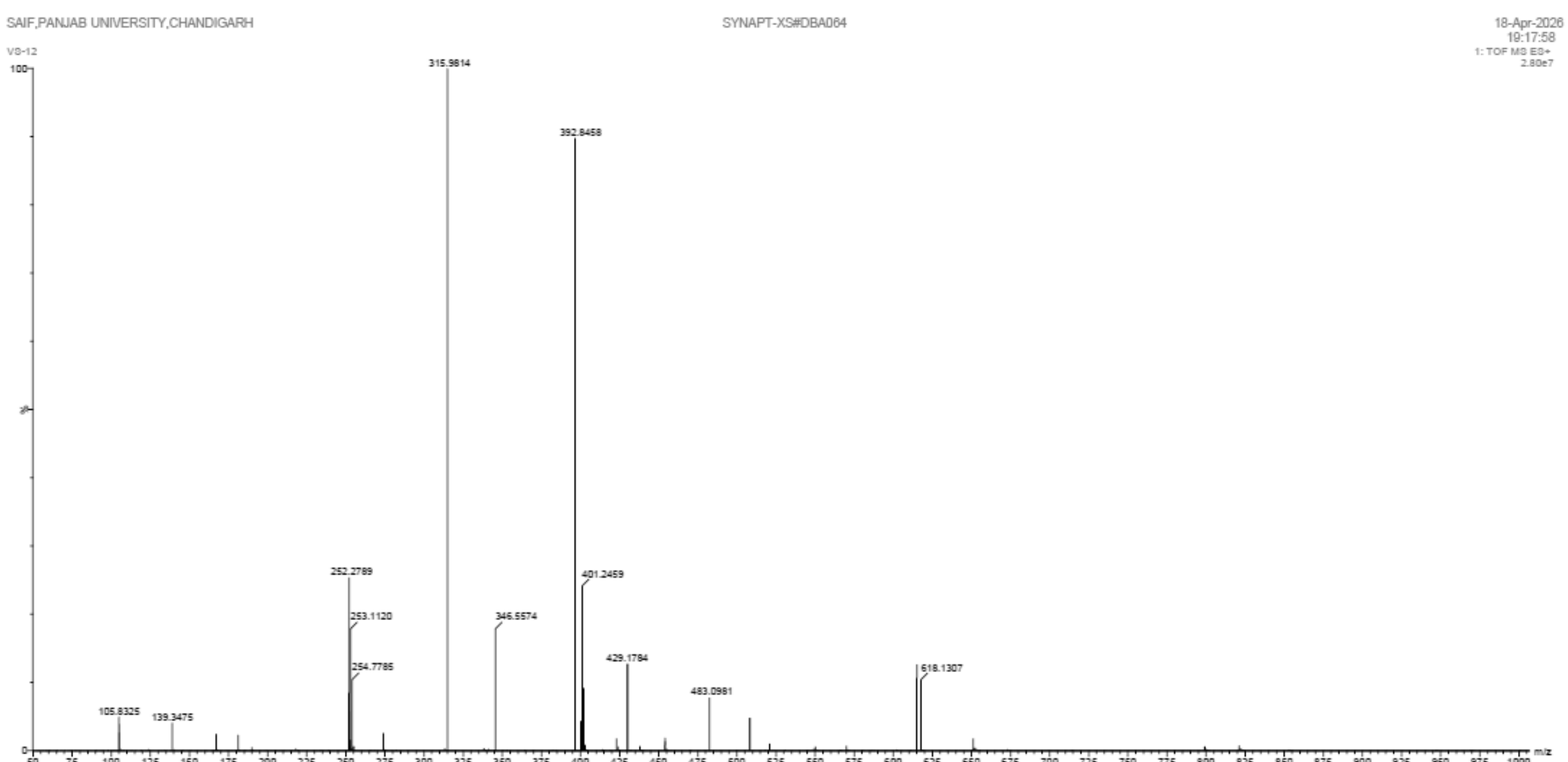

Spectrum 30: Mass Spectra of **VS-12**

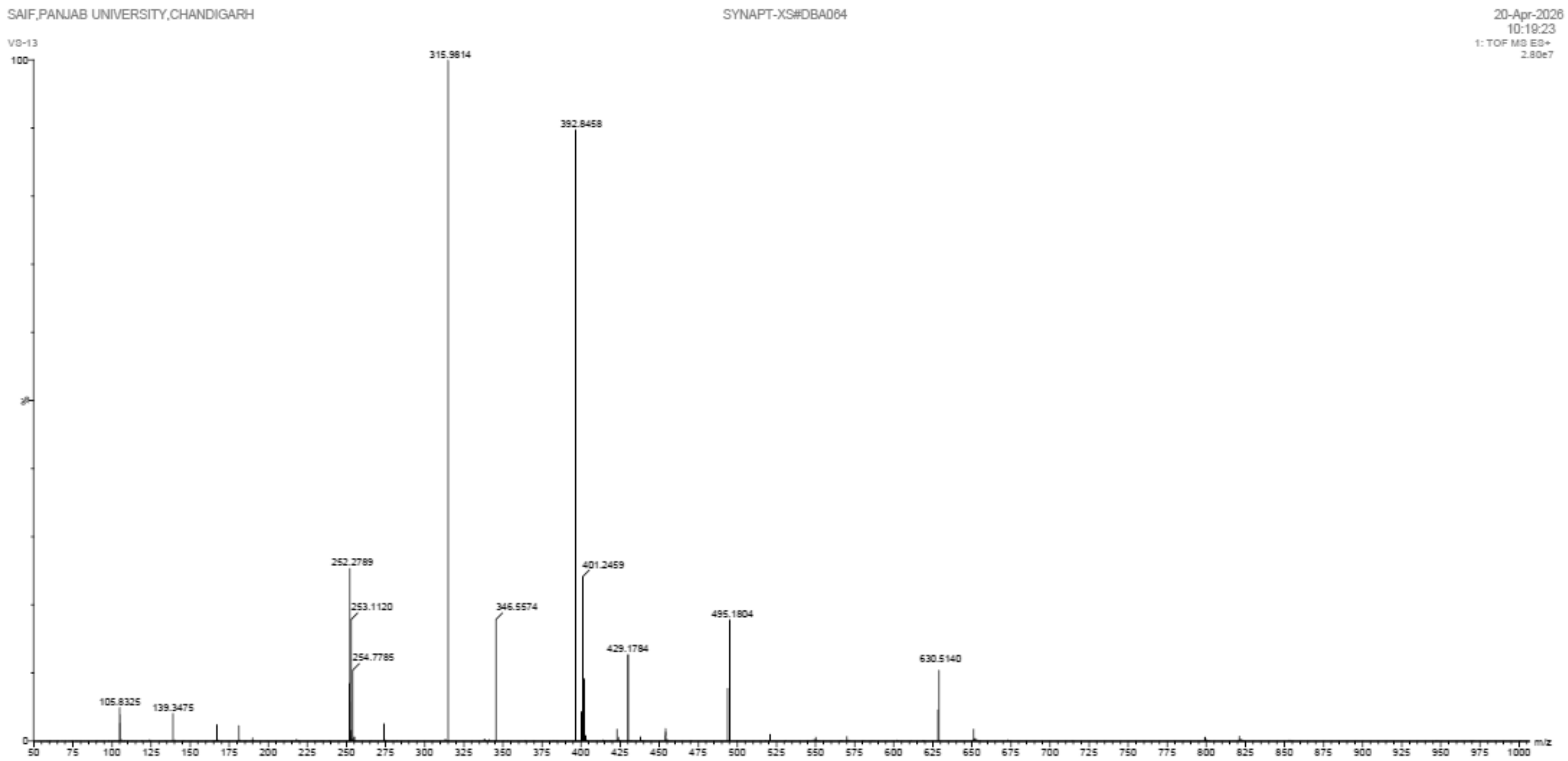

Spectrum 31: Mass Spectra of **VS-13**

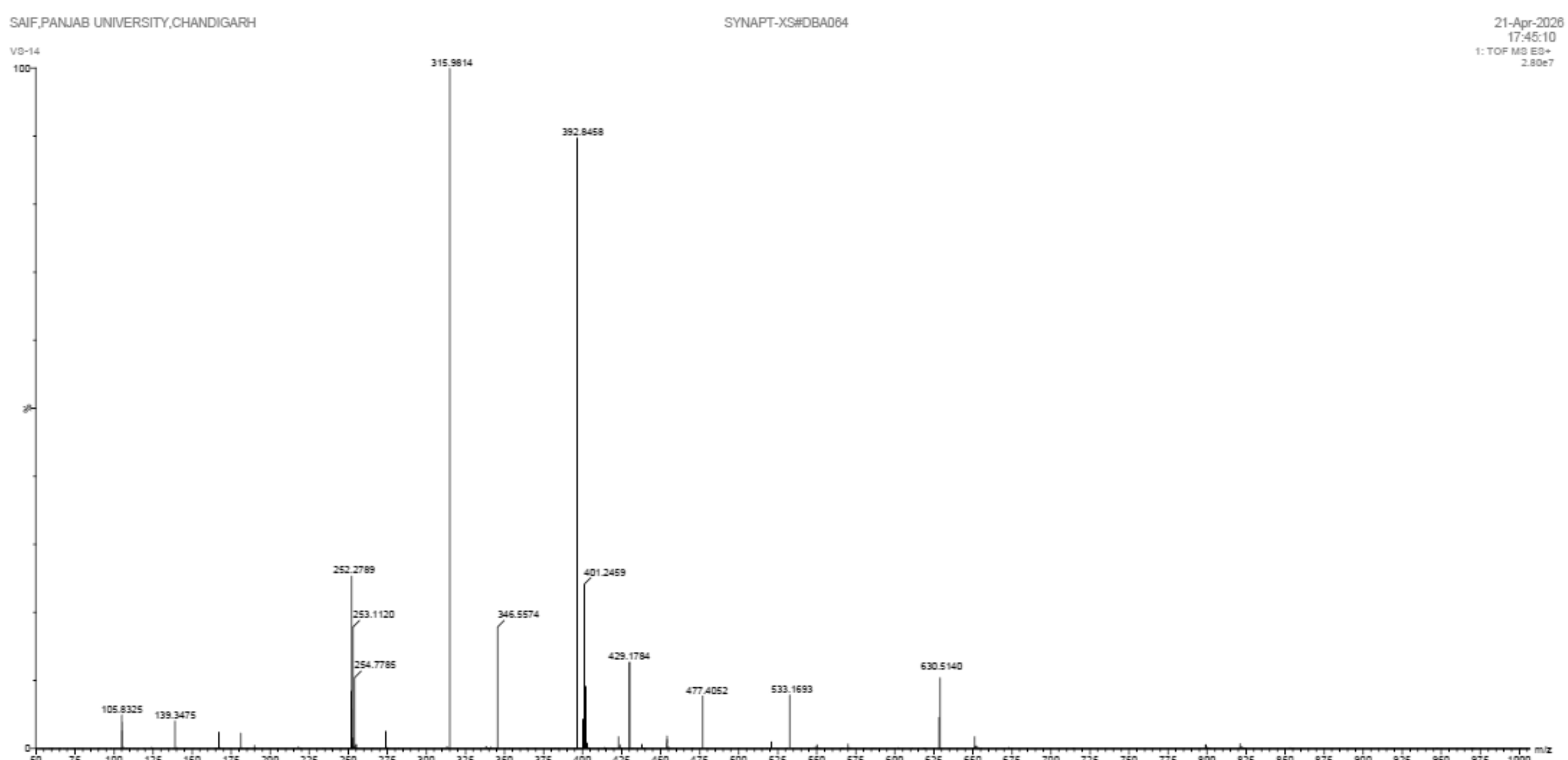

Spectrum 32: Mass Spectra of **VS-14**

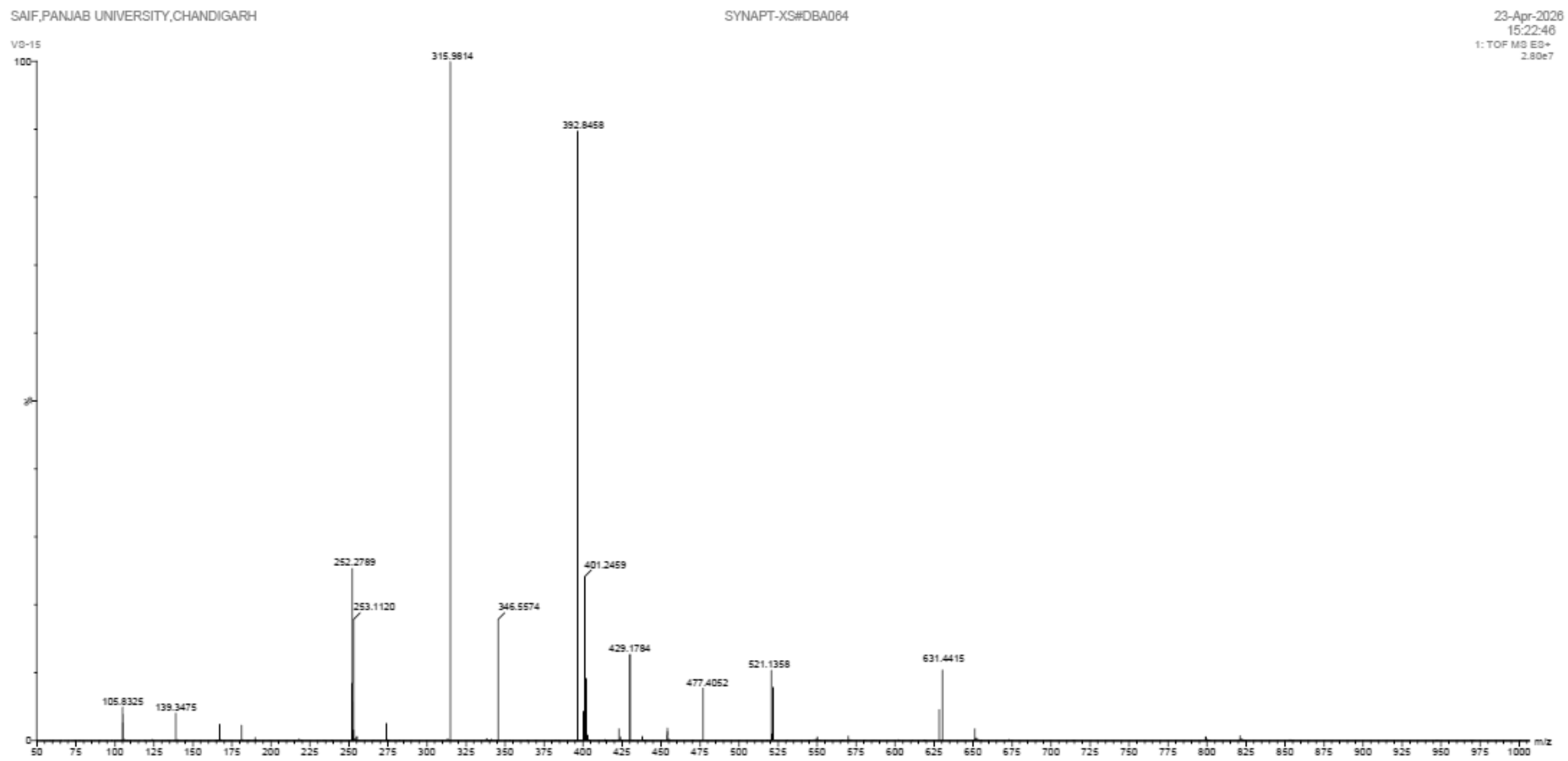

Spectrum 33: Mass Spectra of **VS-15**

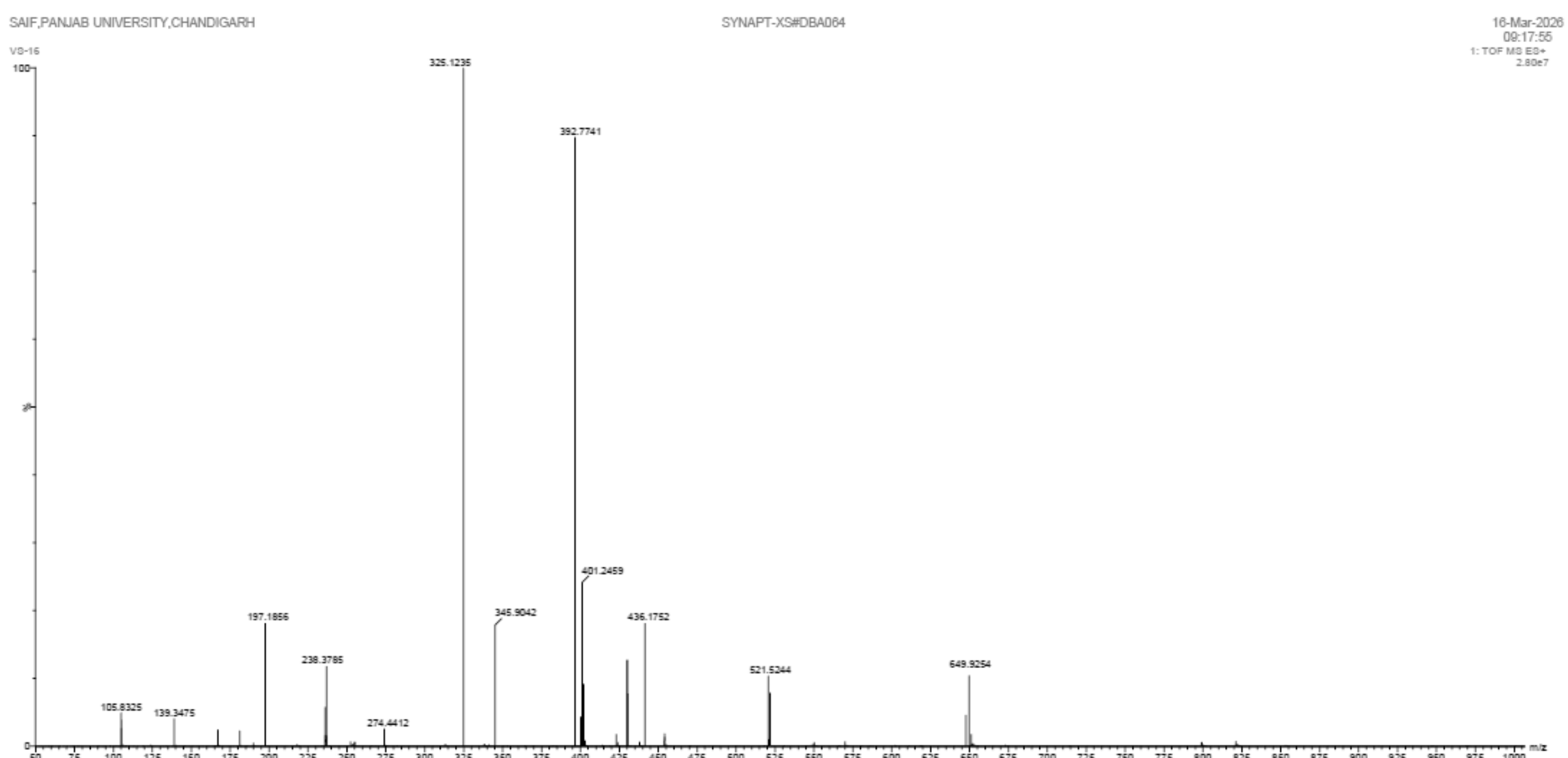

Spectrum 34: Mass Spectra of **VS-16**

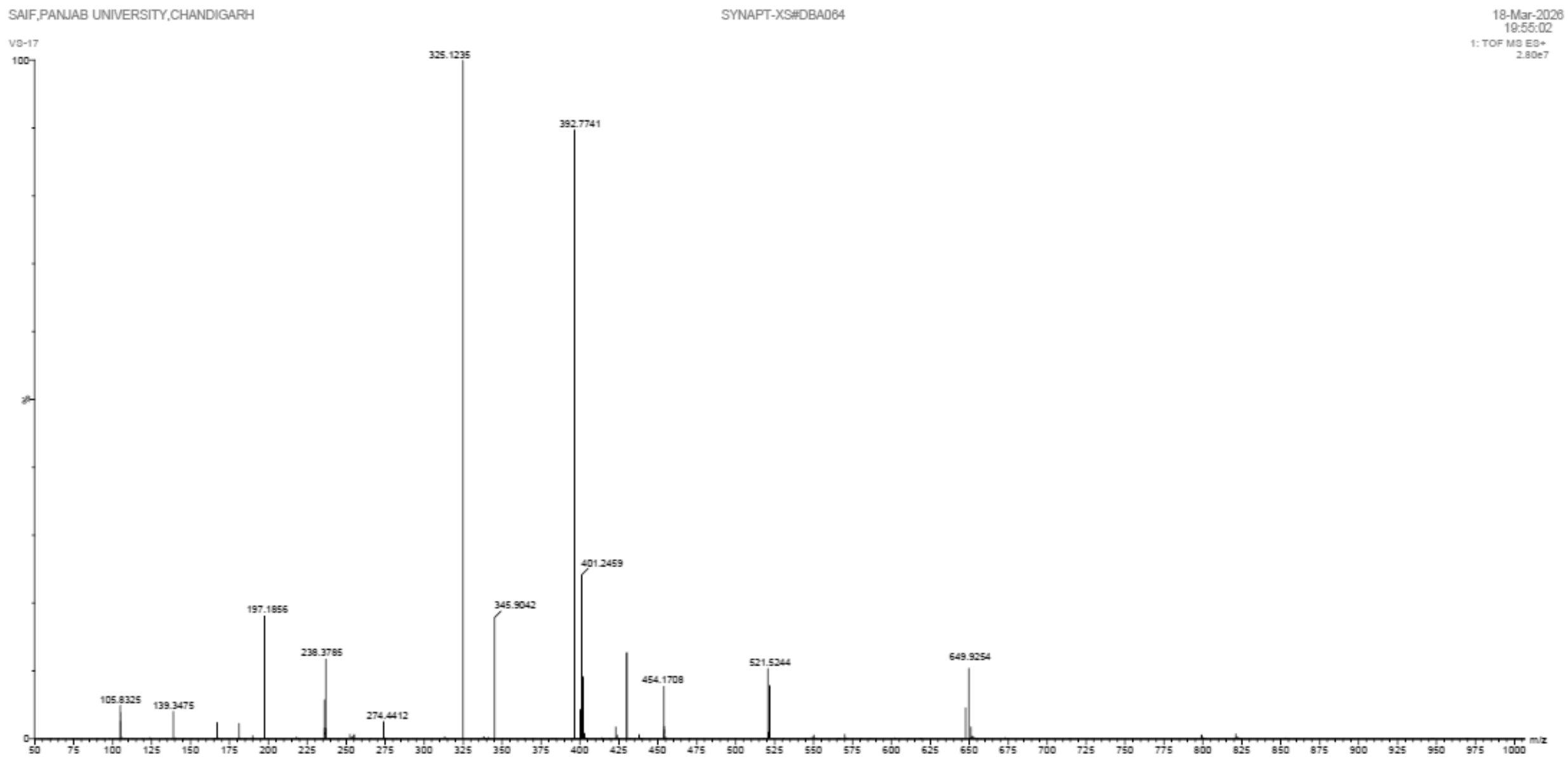

Spectrum 35: Mass Spectra of **VS-17**

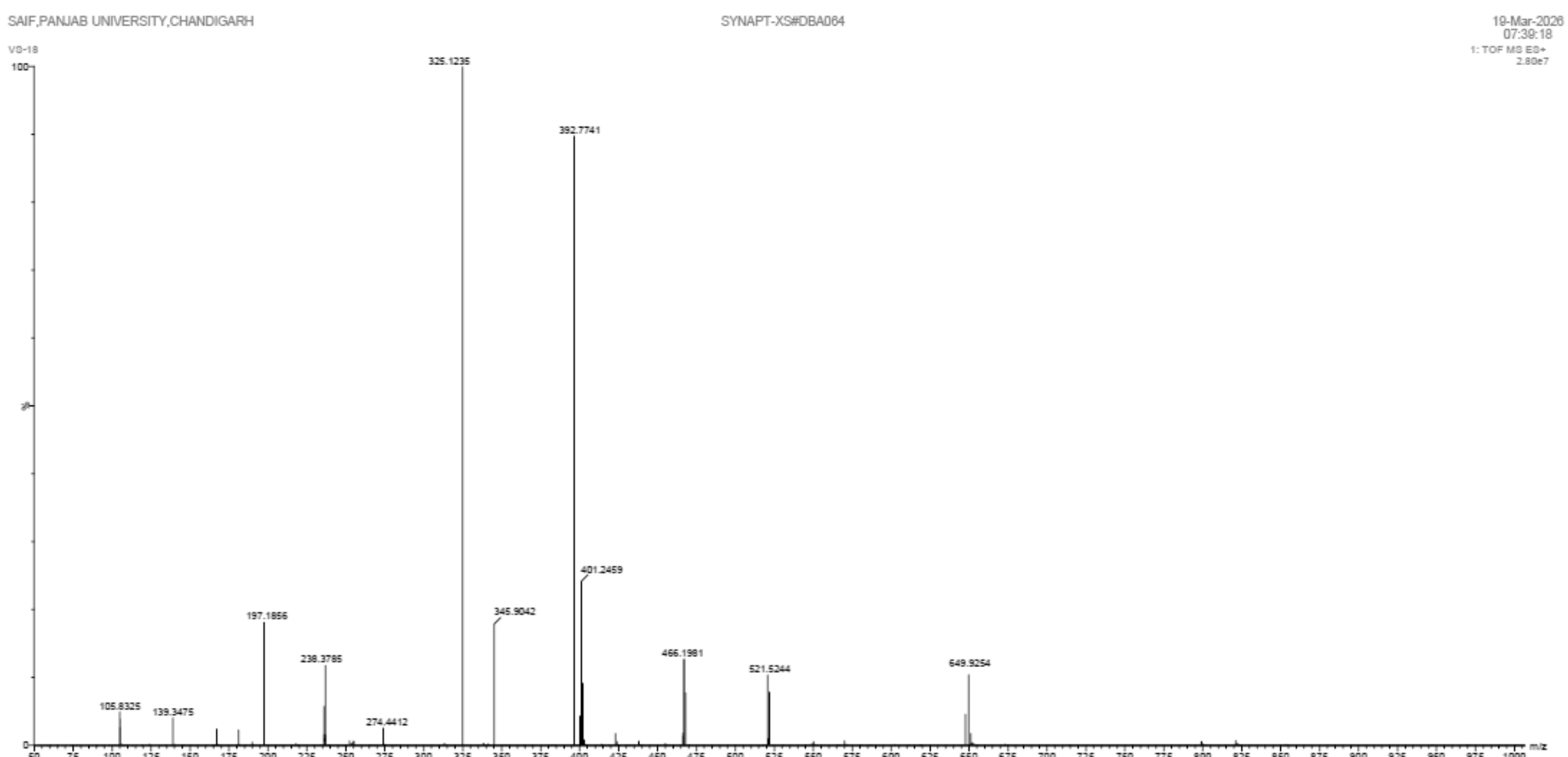

Spectrum 36: Mass Spectra of **VS-18**

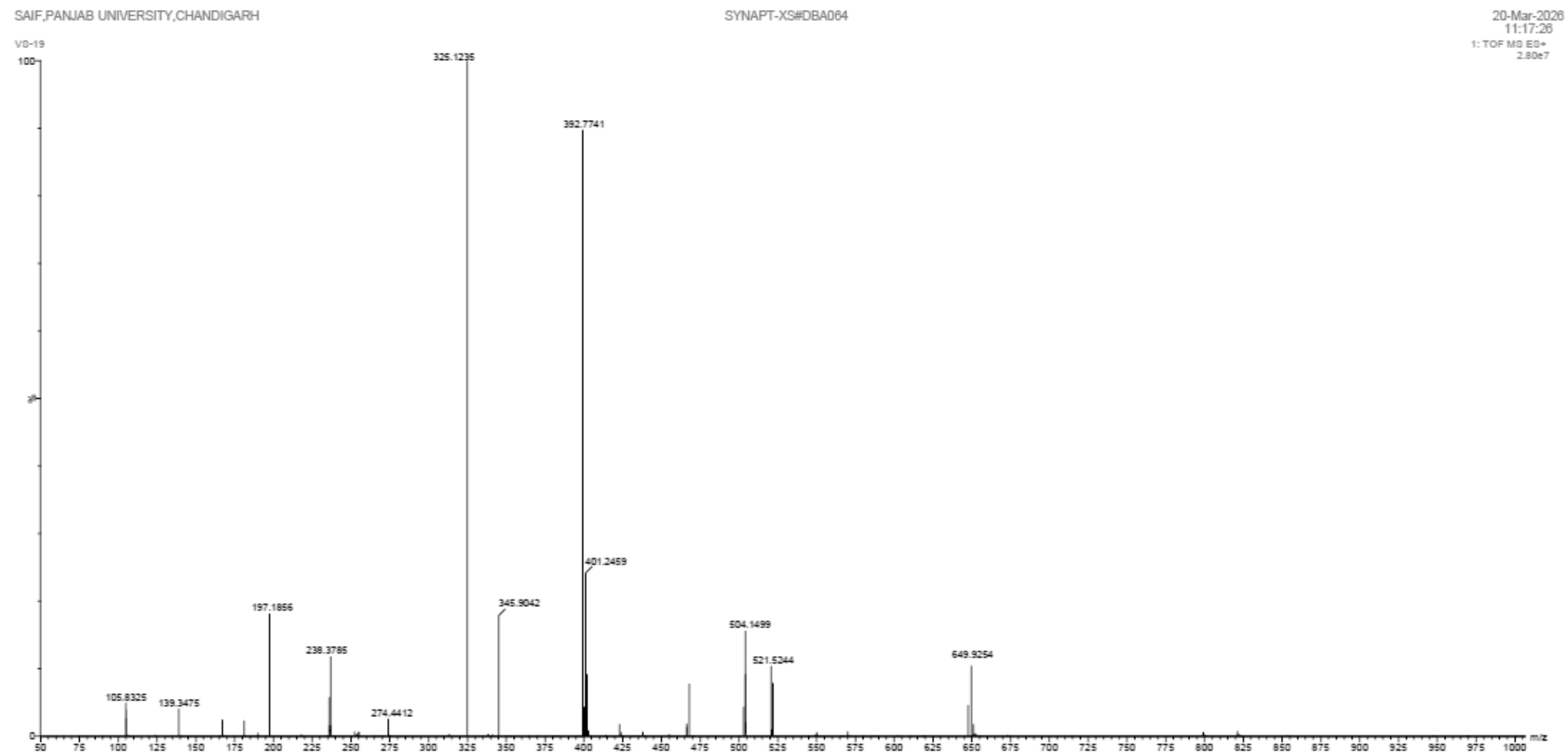

Spectrum 37: Mass Spectra of **VS-19**

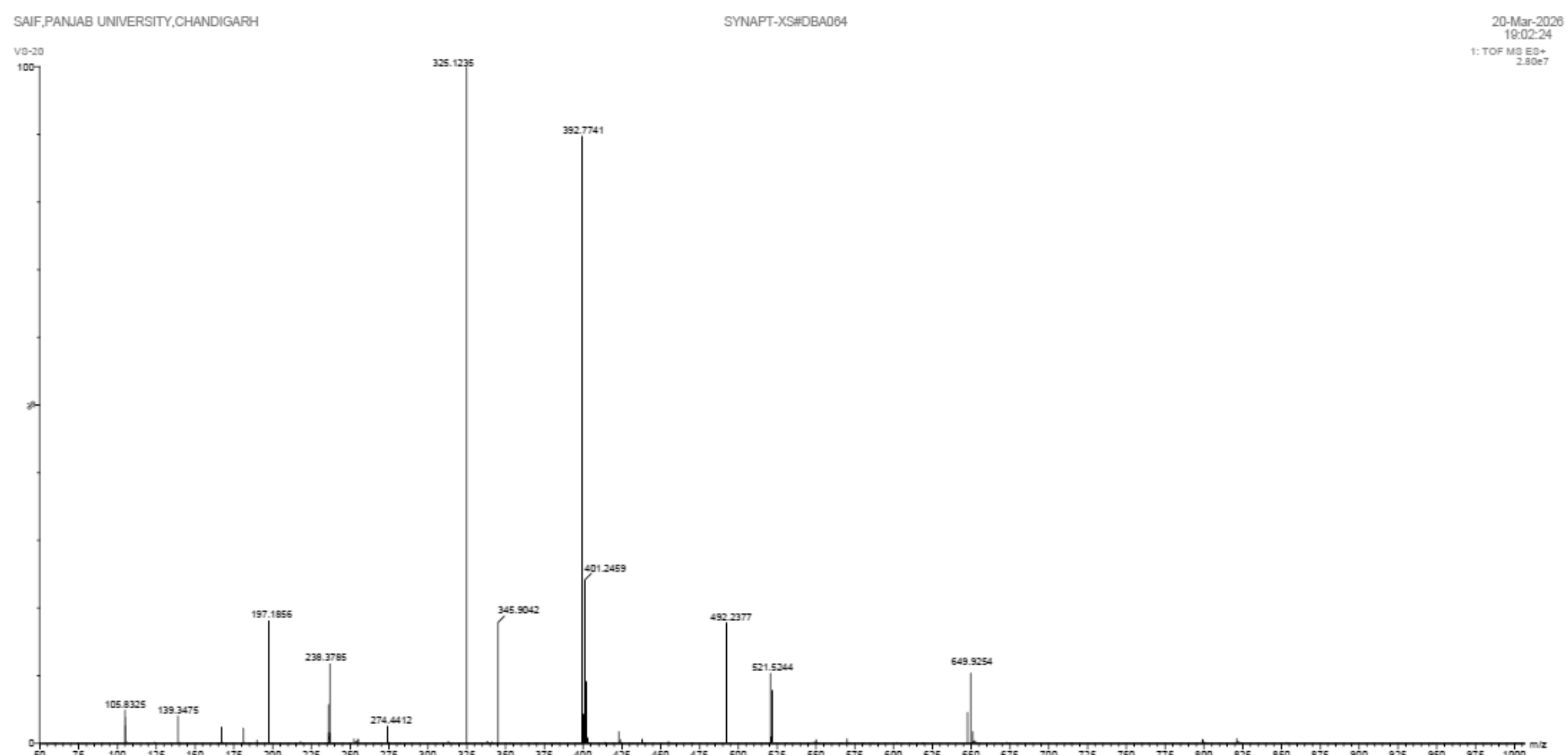

Spectrum 38: Mass Spectra of **VS-20**

Spectrum 39: ^{13}C NMR Spectra of **VS-01**

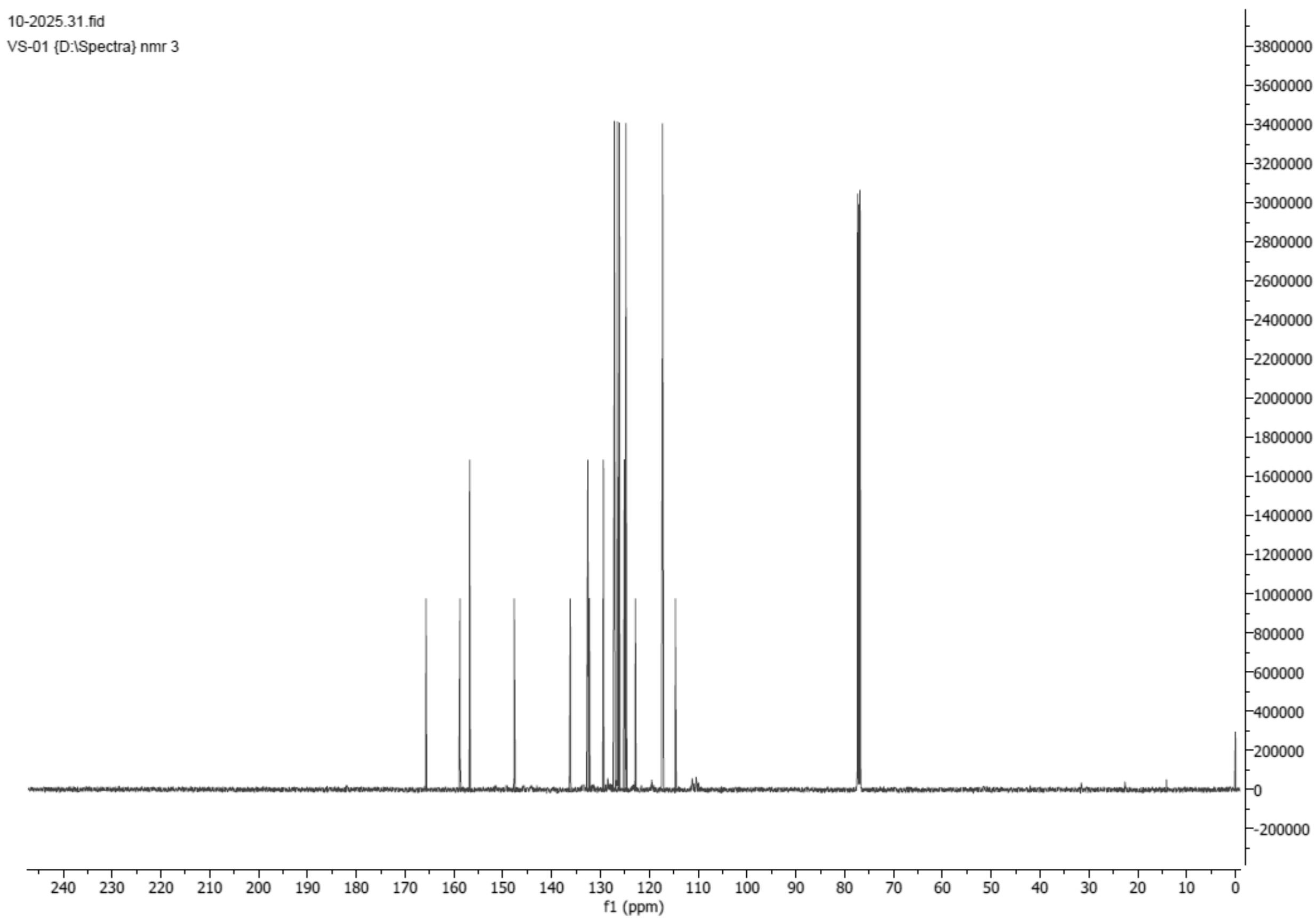

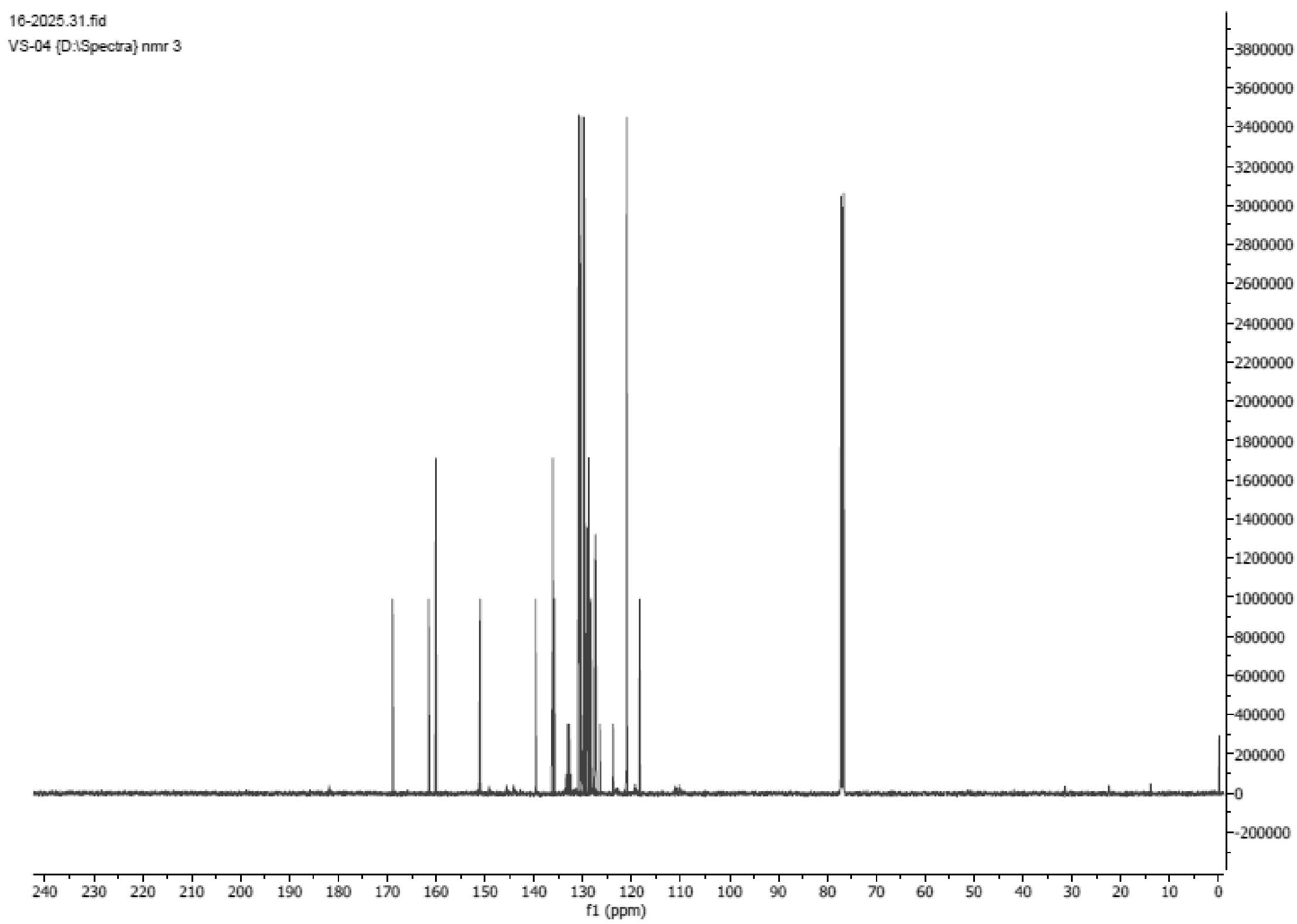

Spectrum 40: ^{13}C NMR Spectra of **VS-04**

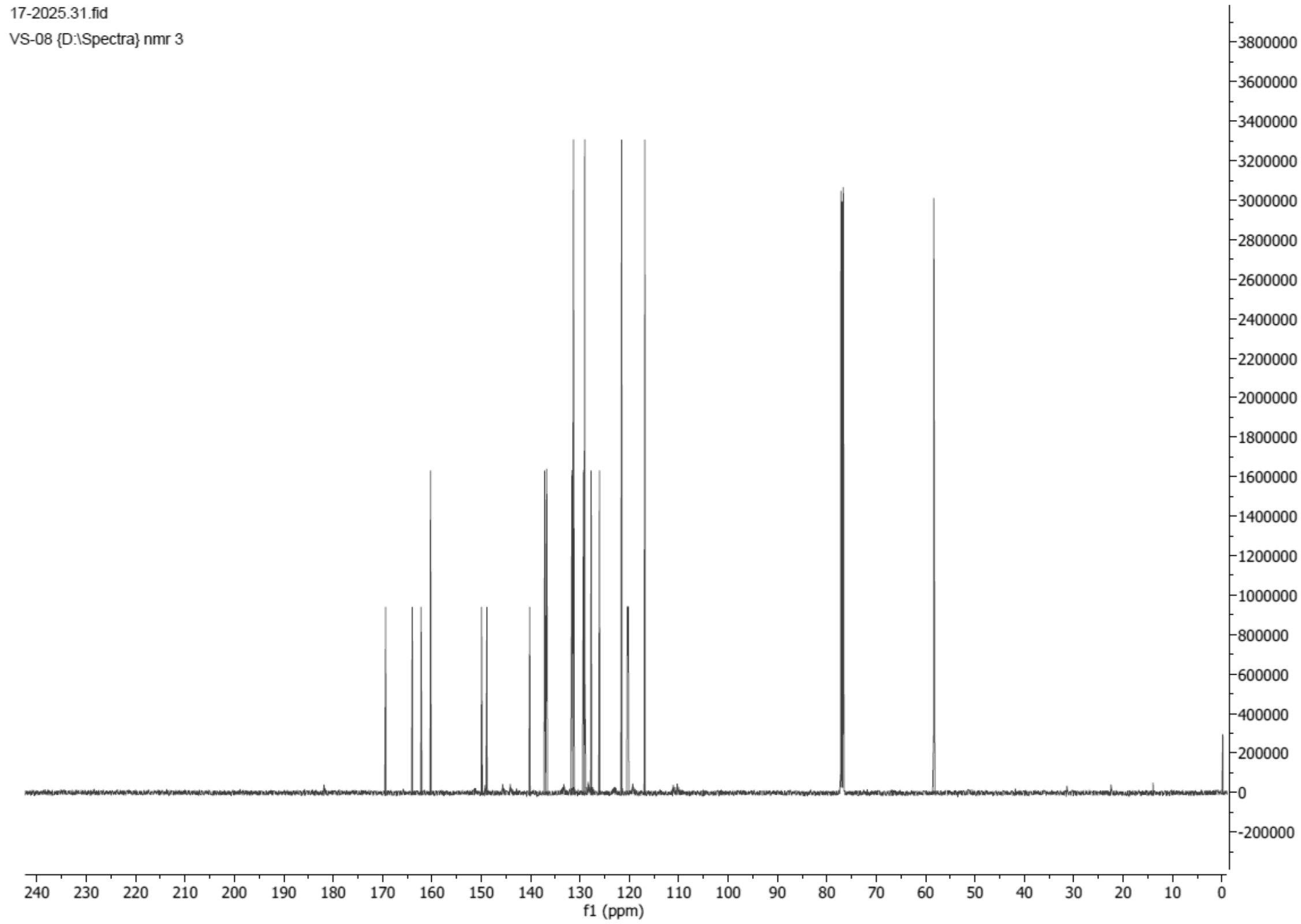

Spectrum 41: ^{13}C NMR Spectra of **VS-07**

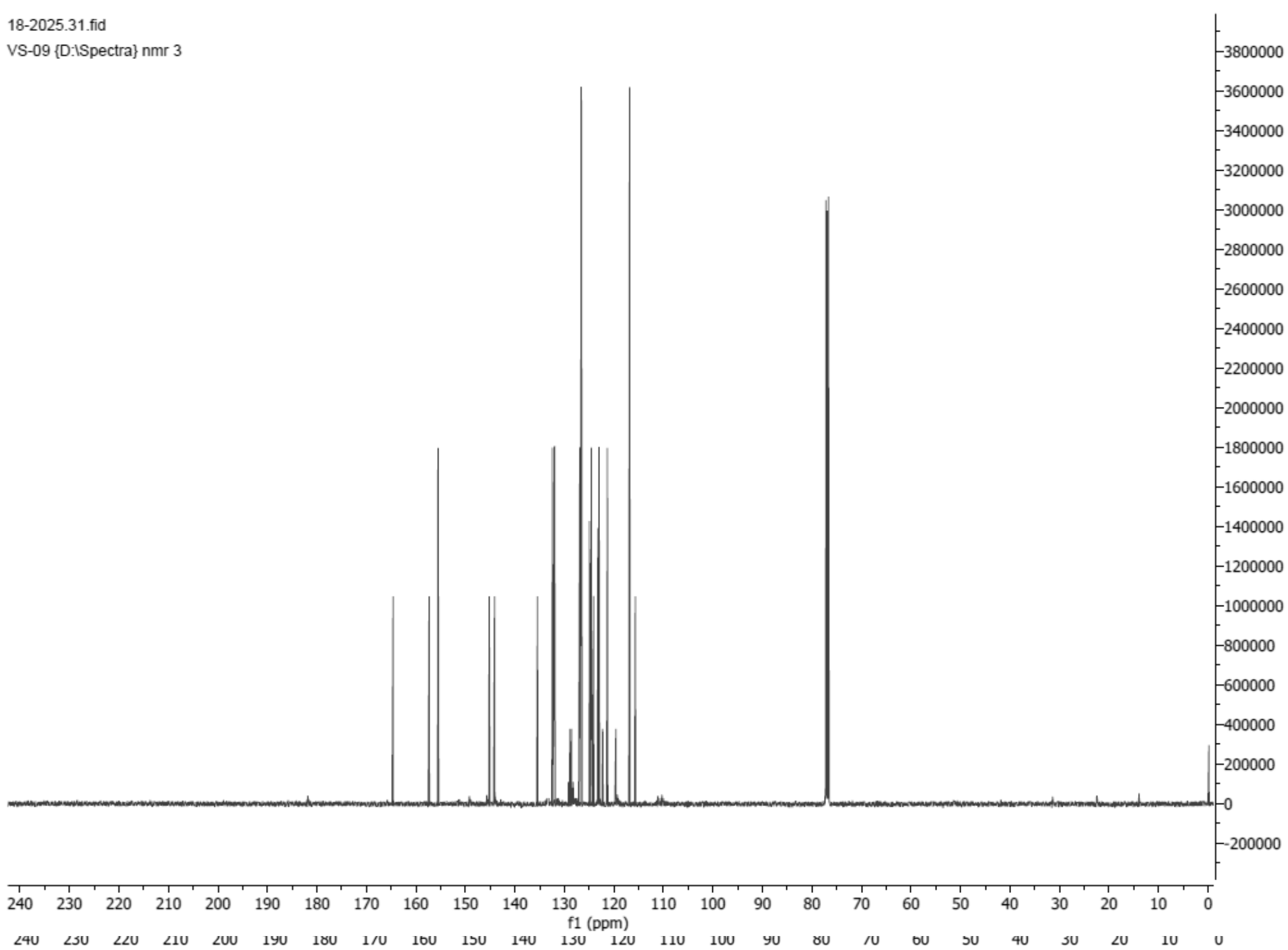

Spectrum 42: ^{13}C NMR Spectra of **VS-08**

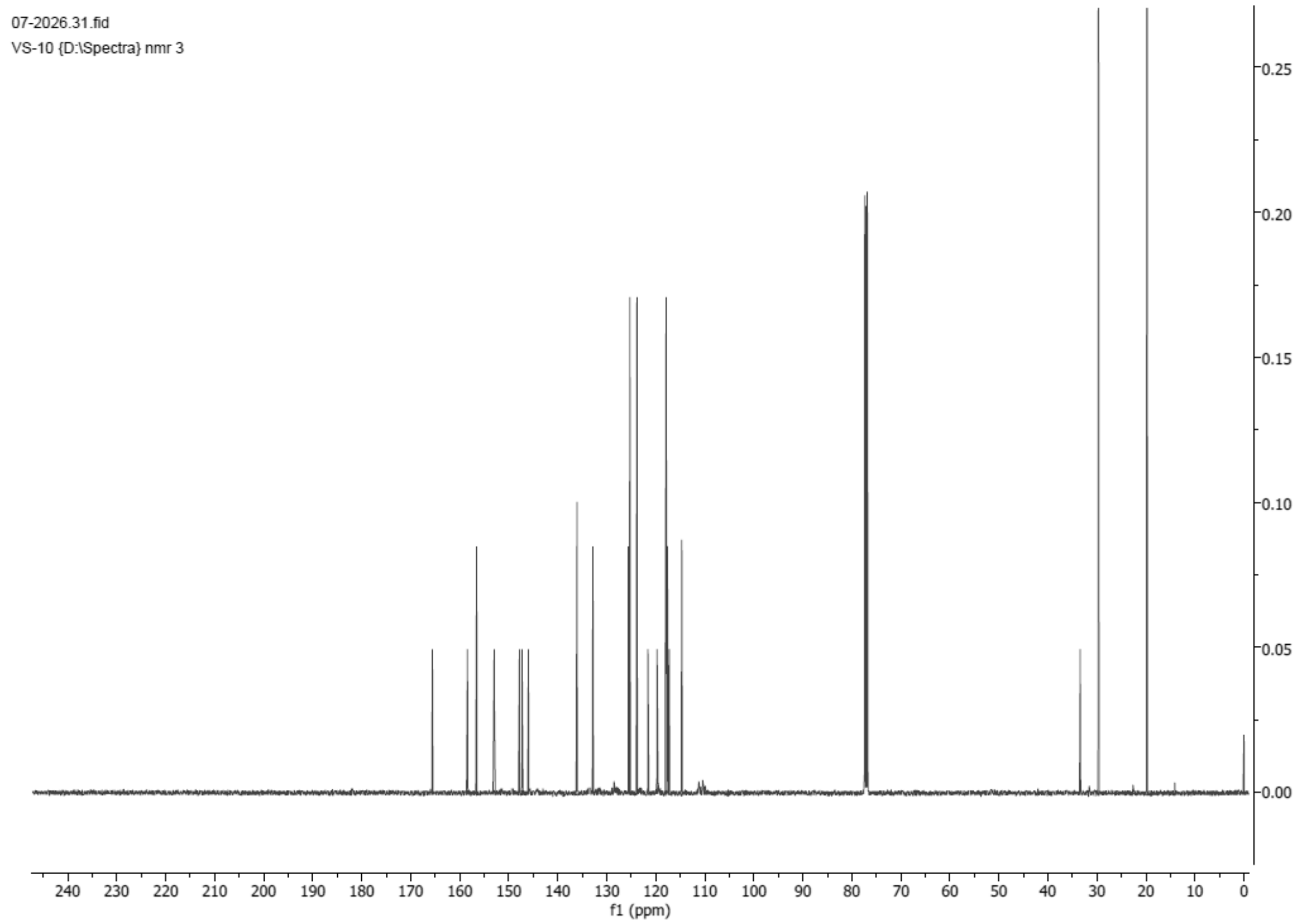

Spectrum 43: ^{13}C NMR Spectra of **VS-09**

Spectrum 44: ^{13}C NMR Spectra of **VS-10**

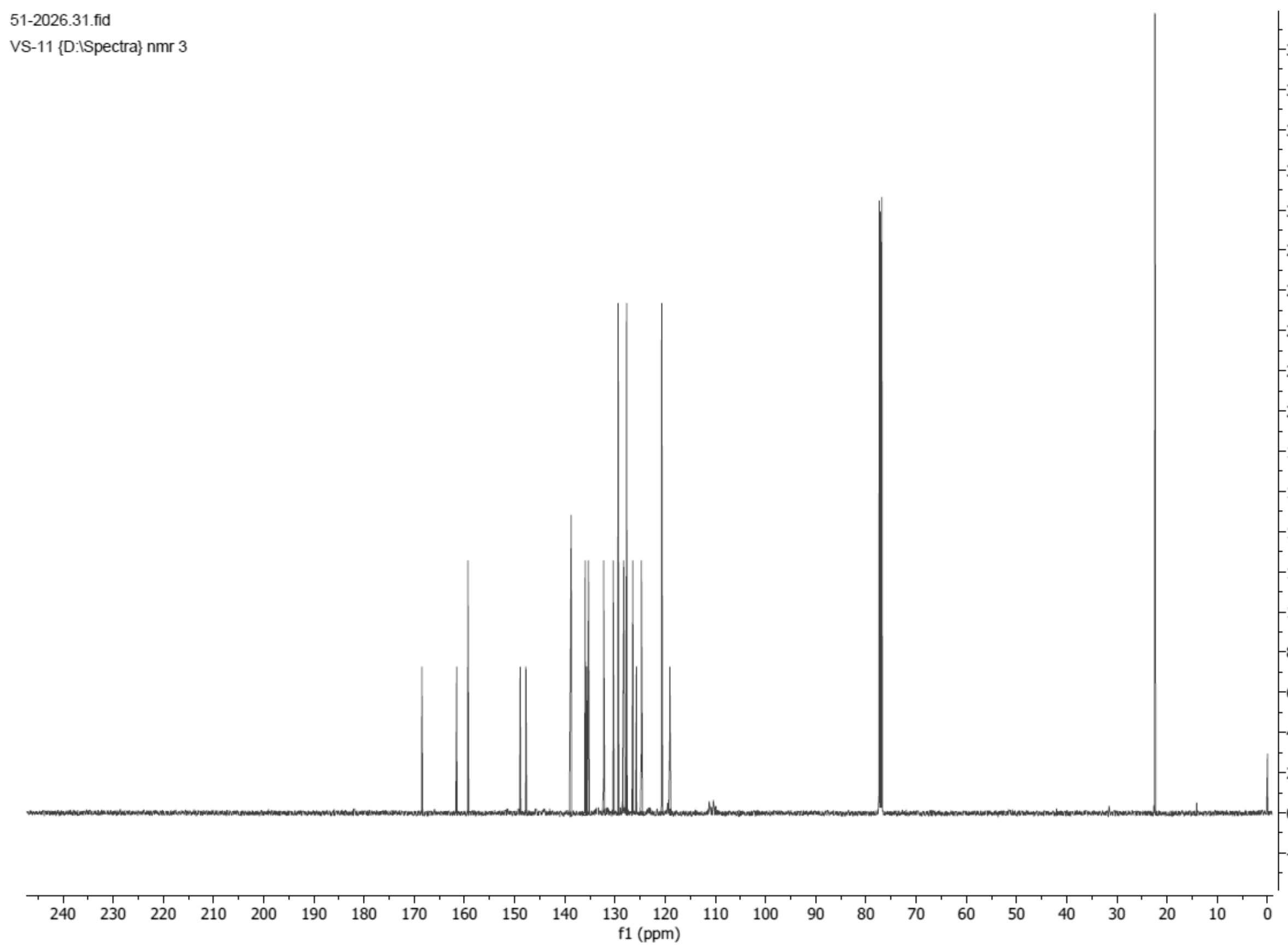

Spectrum 45: ^{13}C NMR Spectra of **VS-11**

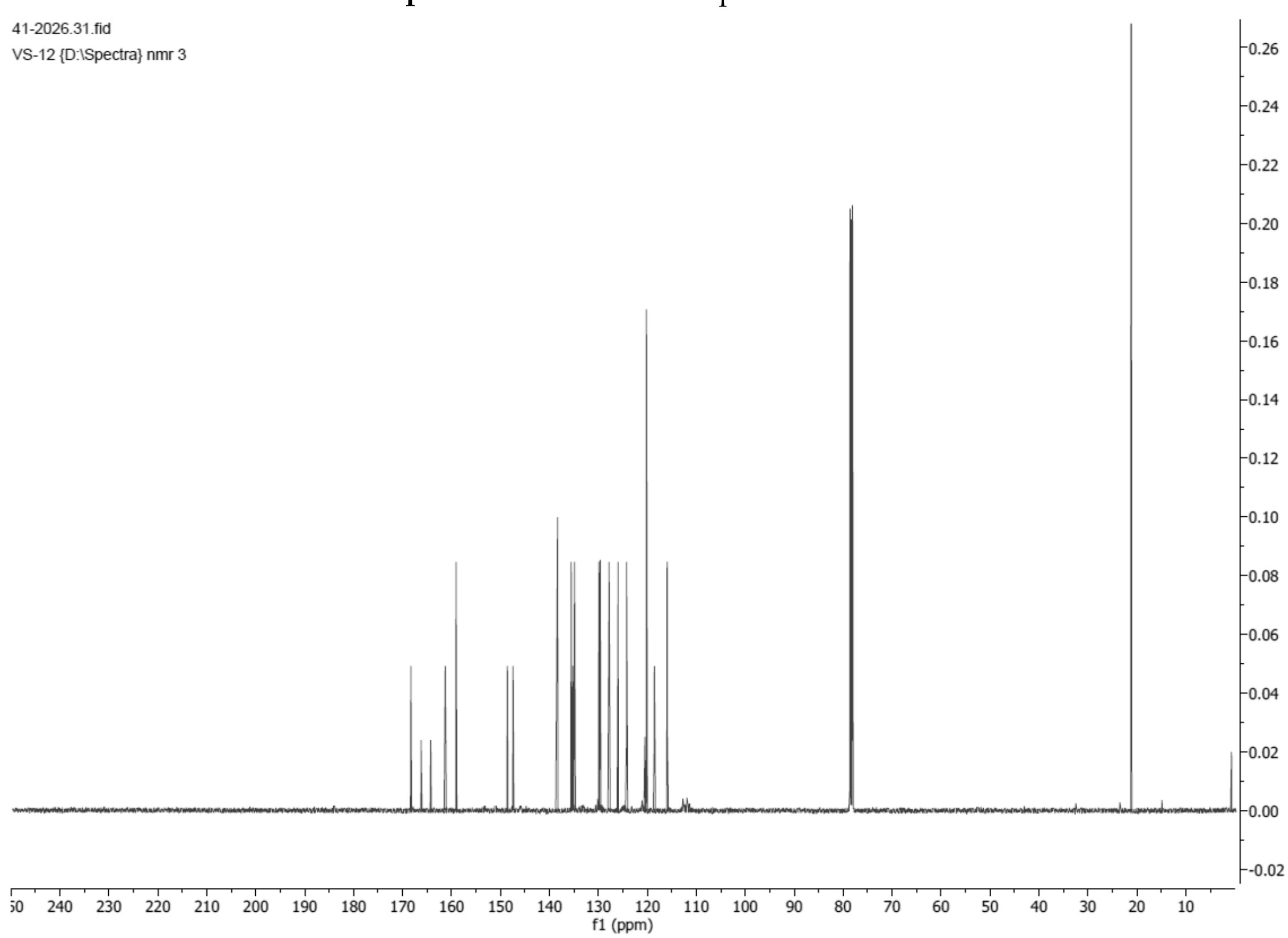

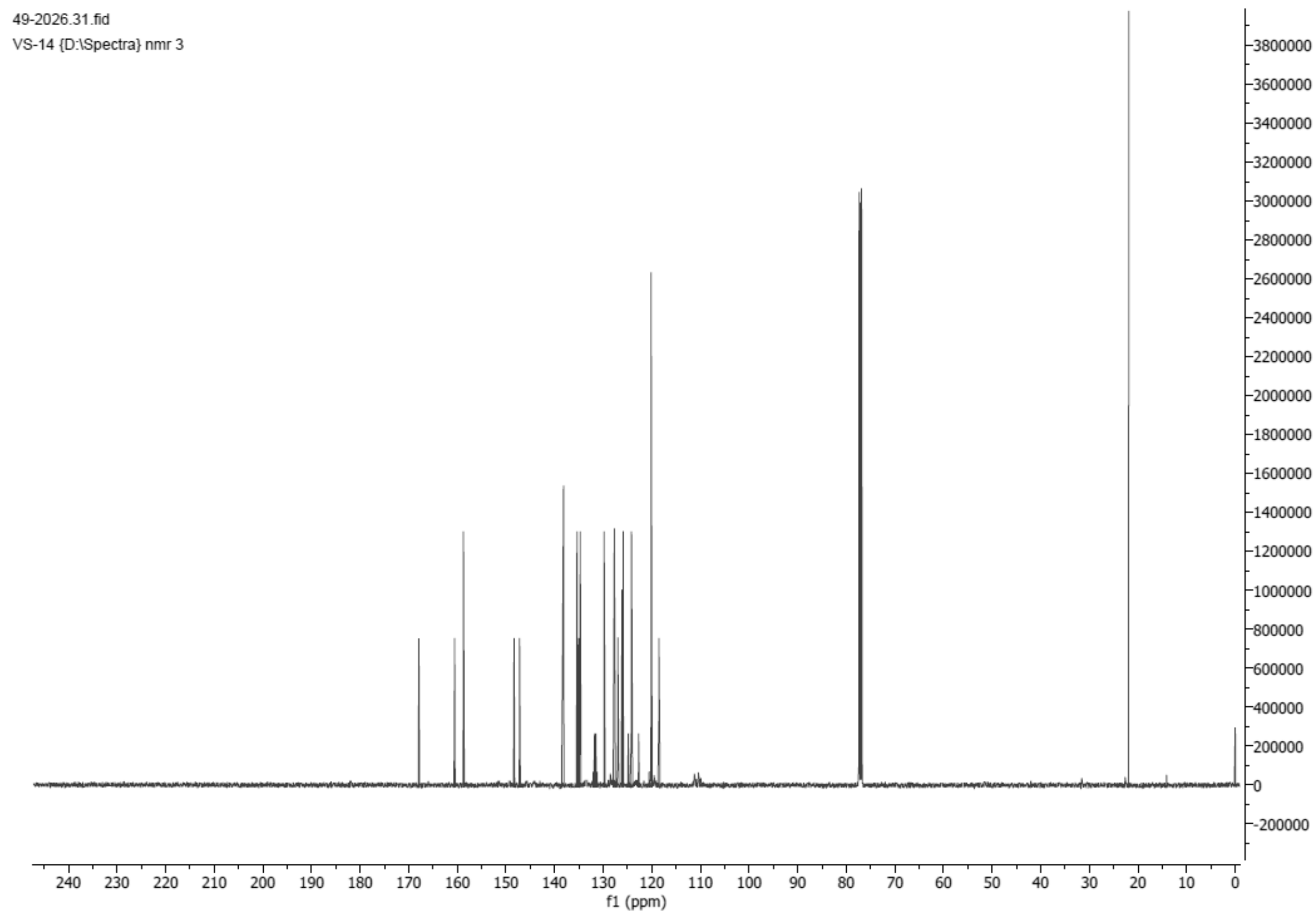

Spectrum 46: ^{13}C NMR Spectra of **VS-12**
Spectrum 47: ^{13}C NMR Spectra of **VS-13**
Spectrum 48: ^{13}C NMR Spectra of **VS-14**

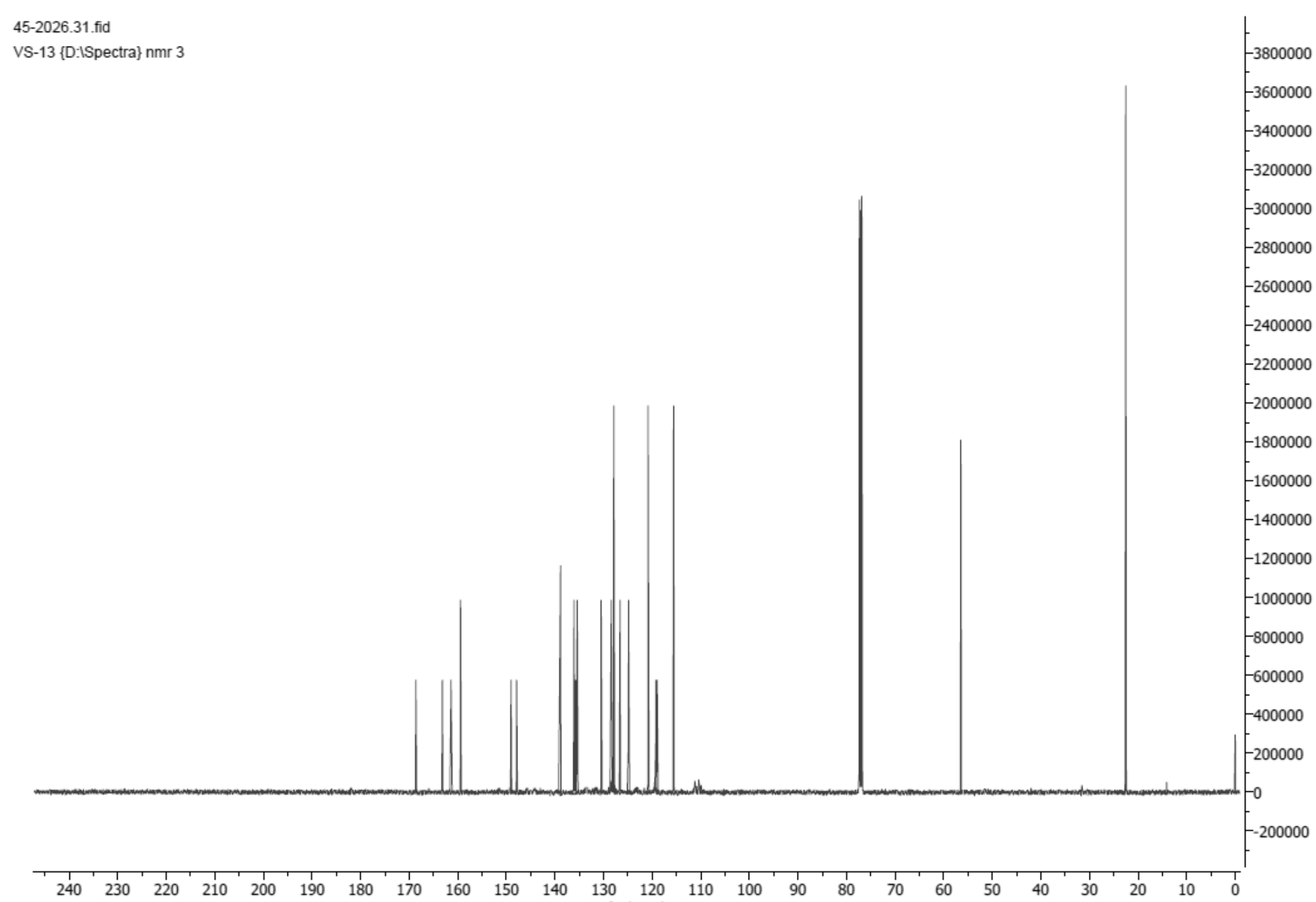

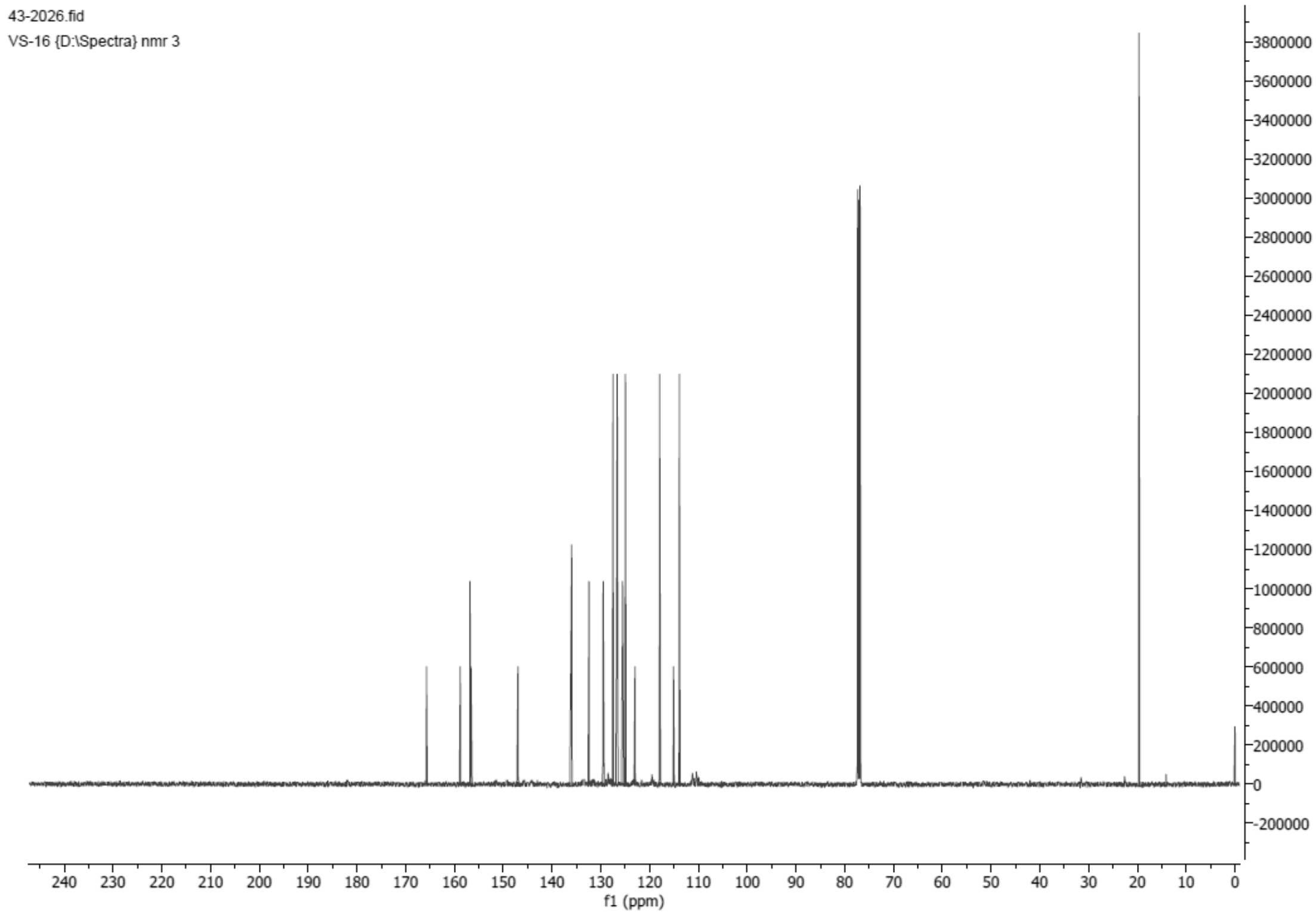

Spectrum 49: ^{13}C NMR Spectra of **VS-15**

Spectrum 50: ^{13}C NMR Spectra of **VS-16**

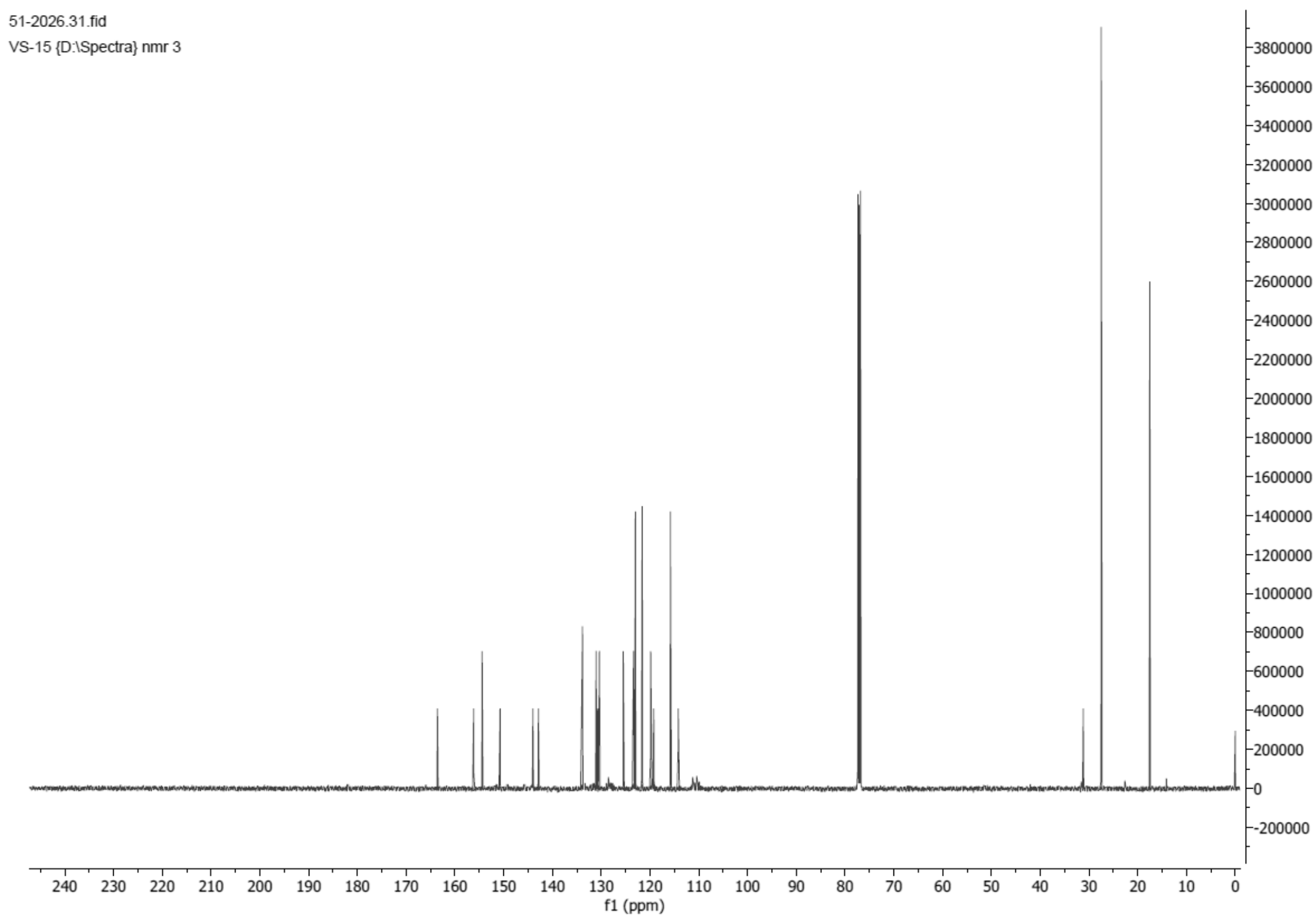

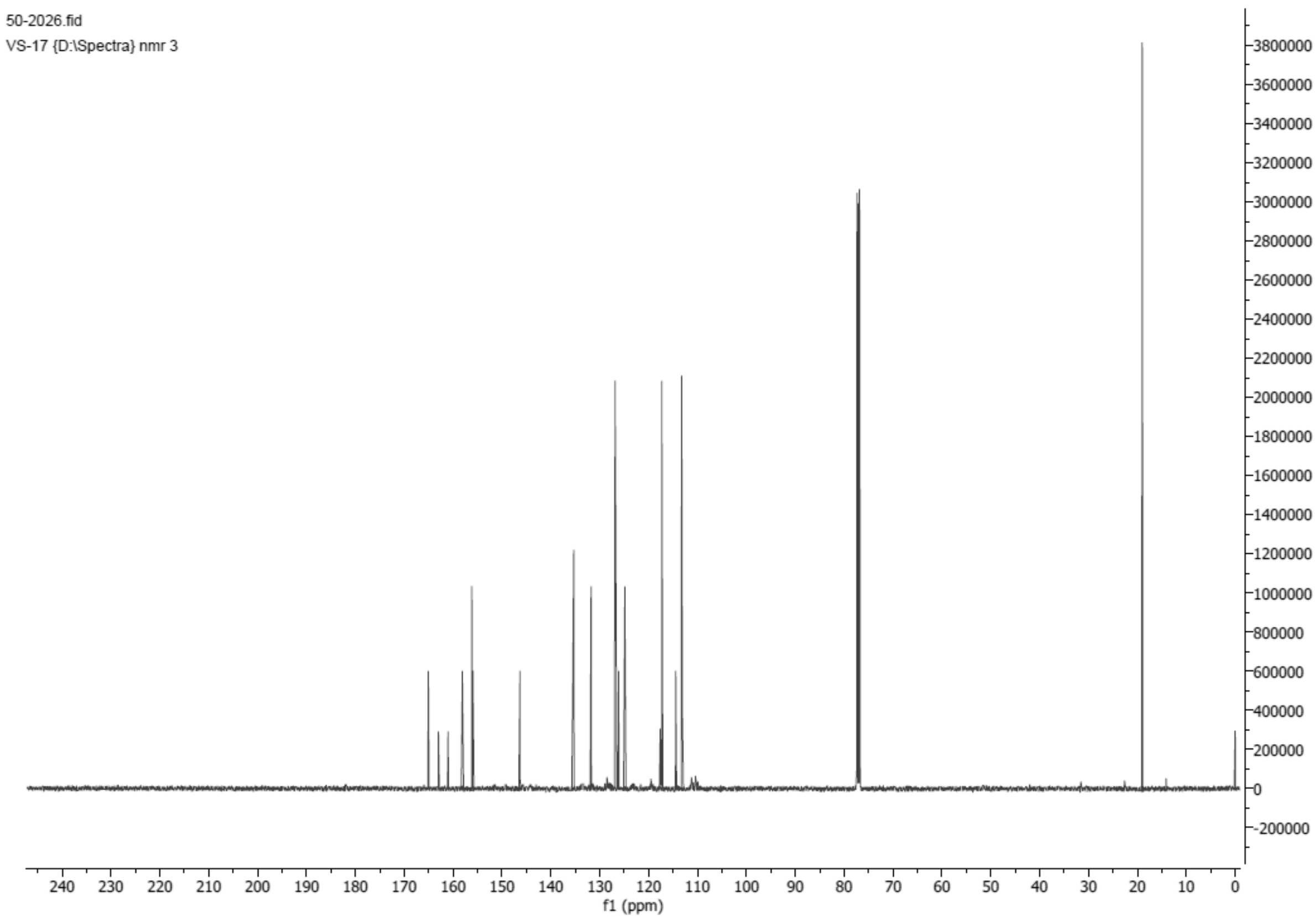

Spectrum 51: ^{13}C NMR Spectra of **VS-17**

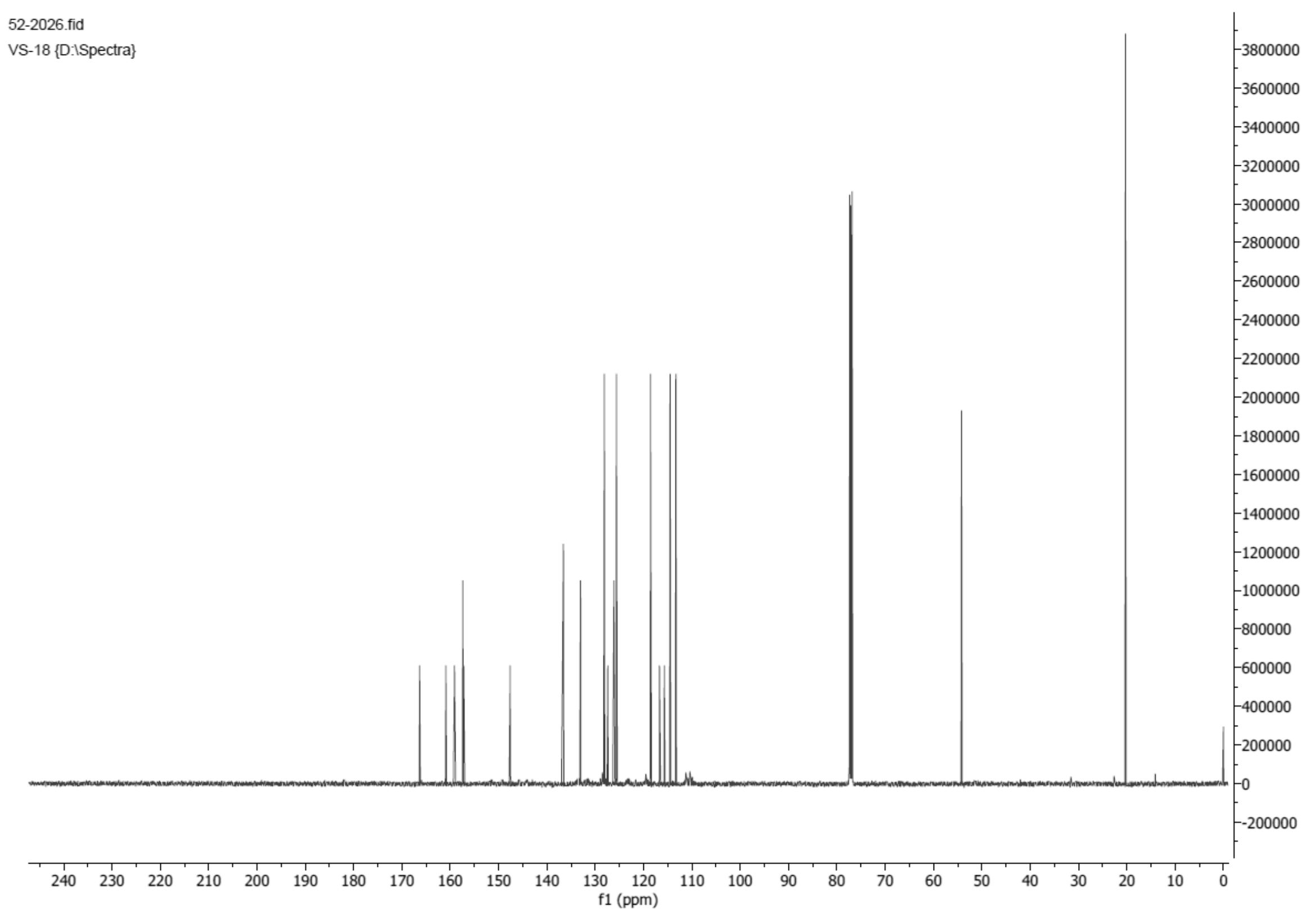

Spectrum 52: ^{13}C NMR Spectra of **VS-18**

Spectrum 53: ^{13}C NMR Spectra of **VS-19**

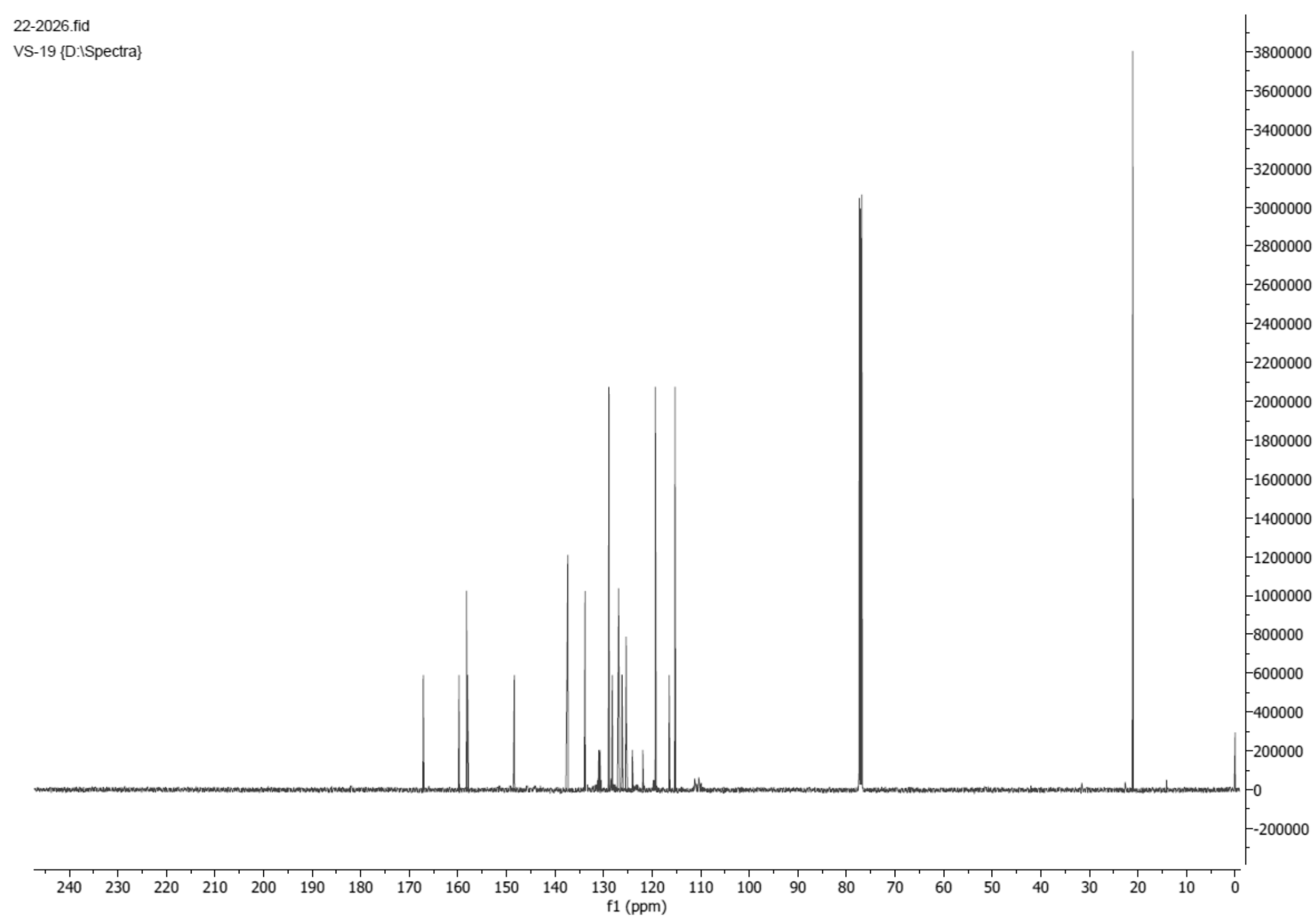

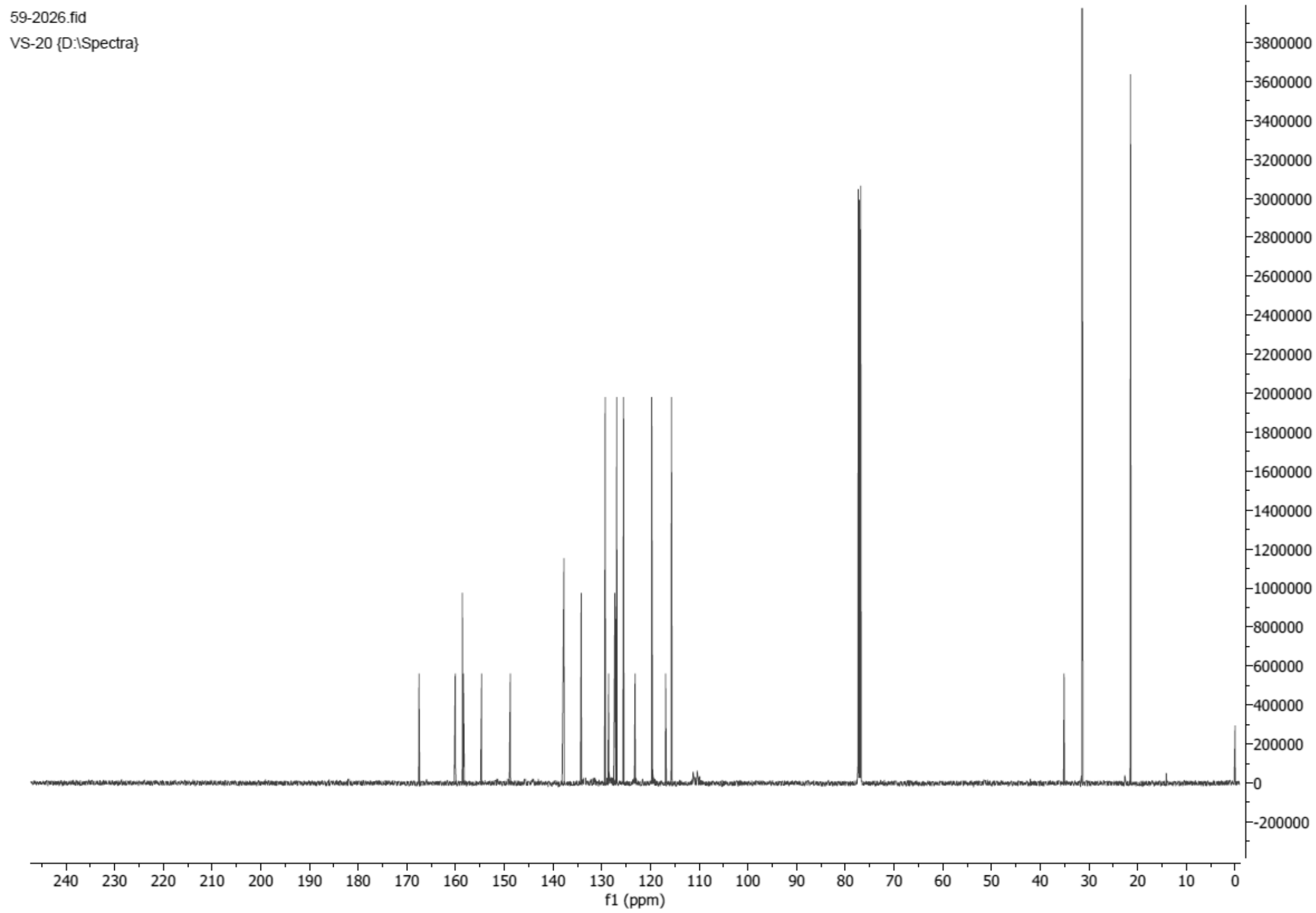

Spectrum 54: ^{13}C NMR Spectra of **VS-20**

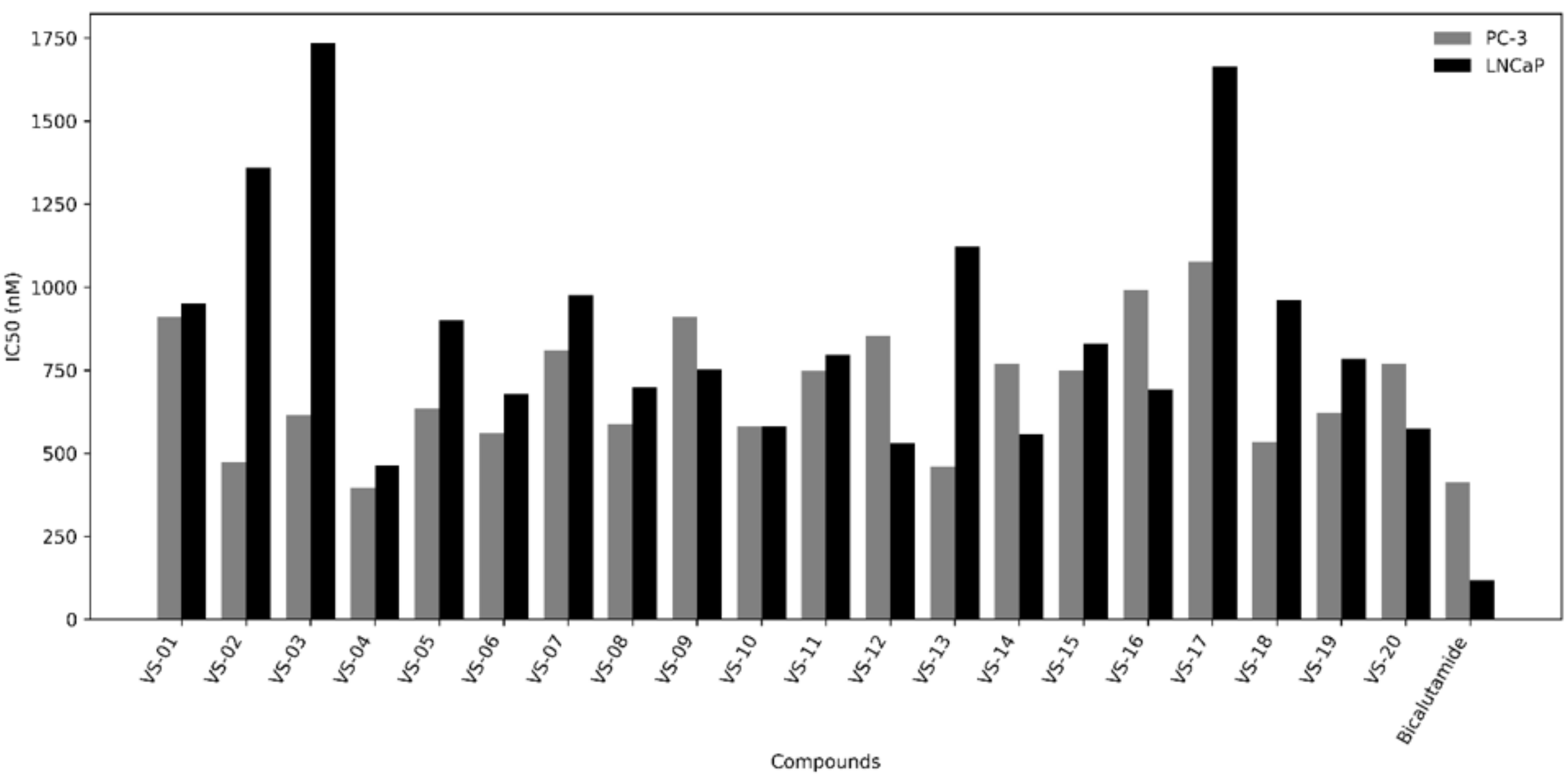

Statistical significance at 1000 nM

Values are p-values versus the corresponding untreated control; **** indicates p < 0.0001.

Compound	PC-3	LNCaP
VS-01	p=2.09e-07 ****	p=1.33e-06 ****
VS-02	p=2.5e-08 ****	p=3.85e-06 ****
VS-03	p=1.32e-07 ****	p=7.37e-06 ****
VS-04	p=3.87e-07 ****	p=3.89e-07 ****
VS-05	p=1.14e-07 ****	p=2.87e-07 ****
VS-06	p=6.67e-08 ****	p=2.93e-07 ****
VS-07	p=2.45e-07 ****	p=8.03e-07 ****
VS-08	p=1.64e-07 ****	p=2.9e-07 ****
VS-09	p=3.31e-08 ****	p=1.31e-07 ****
VS-10	p=2.23e-07 ****	p=2.71e-07 ****
VS-11	p=1.94e-07 ****	p=9.34e-07 ****

Compound	PC-3	LNCaP
VS-12	p=3.74e-07 ****	not provided
VS-13	p=8.89e-08 ****	p=2.14e-06 ****
VS-14	p=7.98e-07 ****	p=1.37e-07 ****
VS-15	p=8.04e-07 ****	p=9.07e-07 ****
VS-16	p=1.18e-06 ****	p=5.87e-07 ****
VS-17	p=1.61e-06 ****	p=6.38e-06 ****
VS-18	p=2.25e-07 ****	p=1.4e-06 ****
VS-19	p=2.78e-07 ****	p=5.47e-06 ****
VS-20	p=4.8e-07 ****	p=3.42e-07 ****
Bicalutamide	p=4.14e-09 ****	p=8.57e-08 ****

Figure S4: Comparative antiproliferative activity of the synthesized oxadiazole–pyrazole derivatives (VS-01–VS-20) against PC-3 and LNCaP prostate cancer cells, as determined by the MTT assay. Gray and black bars represent PC-3 and LNCaP cells, respectively. IC_{50} values are expressed as nM. VS-04 exhibited the greatest antiproliferative potency across both cell lines and was selected as the lead compound for subsequent mechanistic investigations.
